# Identification of a moxidectin dose for 4- to 11-year-old children to support registration and potential use for onchocerciasis elimination: results of an open-label pharmacokinetic and safety study

**DOI:** 10.1186/s13071-025-06891-z

**Published:** 2025-07-24

**Authors:** Nicholas O. Opoku, Felix Doe, Mannel E. Agbogah, Rukaya Laryea, Shelter K. Gordor, Bismark S. Donkor, Edwin Anyomitse, Dickson Kugali, Mariëlle van Zutphen-van Geffen, Danielle J. Navarro, Craig R. Rayner, Kashyap Patel, Annette C. Kuesel, Melinda Lowe, Sally Kinrade

**Affiliations:** 1 Department of Epidemiology and Biostatistics School of Public Health, University of Health and Allied Sciences, Hohoe, Ghana; 2 Volta Regional Hospital, Hohoe, Ghana; 3 Certara, Princeton, NJ USA; 4 Monash Institute of Pharmaceutical Sciences, Monash University, Parkville VIC, Australia; 5 UNICEF/UNDP/World Bank/WHO Special Programme for Research and Training in Tropical Diseases (WHO/TDR), World Health Organization, Geneva, Switzerland; 6 Medicines Development for Global Health (MGDH), Melbourne, Australia

**Keywords:** Onchocerciasis, Moxidectin, Paediatric use, Doses for 4-to 11-year-old children, Ghana

## Abstract

**Background:**

The WHO and onchocerciasis-endemic countries target elimination of the transmission of *Onchocerca volvulus*, the parasite causing onchocerciasis, primarily through ivermectin mass drug administration (MDA). In Africa, alternative treatment strategies are required to achieve or accelerate elimination. One of these is the MDA of moxidectin, an anthelmintic drug for which an 8 mg dose to individuals aged 12 years and older has received regulatory approval. This study was conducted to identify dose levels for children aged 4 to 11 years.

**Methods:**

An open-label, non-comparative, single-dose study in an onchocerciasis-endemic area of Ghana was designed to evaluate moxidectin pharmacokinetics and safety in four groups comprising nine individuals each: those aged 12–17 years, after administration of 8 mg; those aged 8–11 years after administration of 8 mg; those aged 8–11 years after administration of 6 mg; and those aged 4–7 years after administration of 4 mg moxidectin. Evaluations to identify adverse events included physical examination, vital signs, haematology, clinical chemistry and 12-lead electrocardiograms. Moxidectin plasma concentrations were measured at 1, 2, 4, 8, 24 and 72 h and at 1, 2, 4 and 12 weeks post-dose. The maximum concentration (C_max_; ng/ml) and area under the concentration–time curve from dosing to infinity (AUC_0-∞_; ng h/ml) were obtained through non-compartmental analysis.

**Results:**

The 38 adverse events in 24 participants were illnesses typically seen in the study population without treatment (primarily malaria) and were considered not moxidectin-related. The arithmetic mean (± standard deviation) C_max_ and AUC_0-∞_ in all groups (range 84.6 ± 18.2 to 118 ± 26.5 ng/ml and 2070 ± 587 to 3910 ± 1070 ng h/ml, respectively) were lower than in those in healthy adults administered 36 mg moxidectin (range 252 ± 50.3 to 296 ± 47 ng/ml and 10835 ± 2587 to 14972 ± 2233 ng h/ml, respectively), the highest and well-tolerated dose given to humans. Mean C_max_ and AUC_0-∞_ in groups 1–3 (range 84.6 ± 18.2 to 118 ± 26.5 ng/ml and 2920 ± 2120 to 3910 ± 1070 ng h/ml, respectively) exceeded those in *O. volvulus*-infected adults administered 8 mg (63.1 ± 20.0 ng/ml and 2738 ± 1606 ng h/ml, respectively). In group 4, mean C_max_ (89.4 ± 24.1 ng/ml) exceeded that in *O. volvulus*-infected adults administered 8 mg moxidectin (63.1 ± 20.0 ng/ml). Mean AUC_0-∞_ (2070 ± 587 ng h/ml) exceeded that in *O. volvulus*-infected adults administered 4 mg moxidectin (1169 ± 488 ng h/ml), a dose shown to be almost as efficacious as 8 mg.

**Conclusions:**

Considering the safety data, exposures and potential MDA operational aspects, 8 mg for children aged between 8 and 11 years and 4 mg for children aged between 4 and 7 years were selected for further single-dose studies.

**Graphical abstract:**

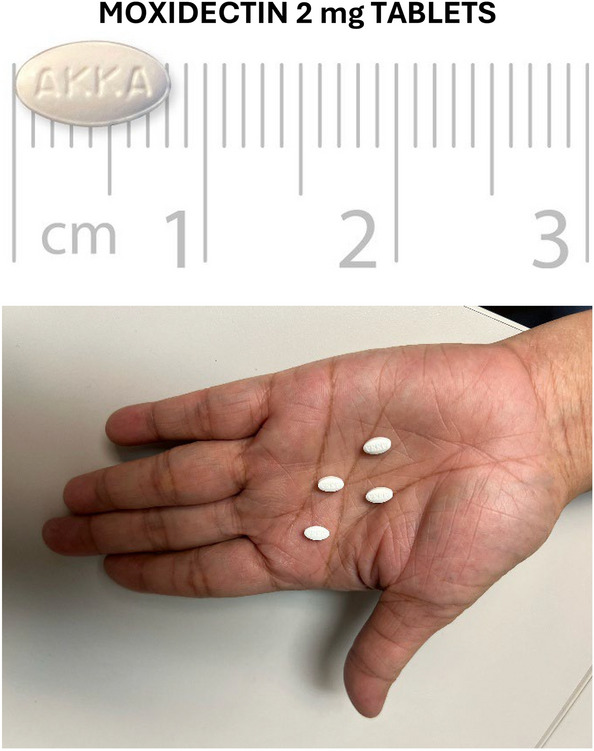

**Supplementary Information:**

The online version contains supplementary material available at 10.1186/s13071-025-06891-z.

## Background

Onchocerciasis, a *Simulium*-borne parasitic disease caused by *Onchocerca volvulus* is targeted for elimination (interruption of parasite transmission). The WHO and endemic countries aim for WHO-verified elimination in 12 countries and for the mass drug administration (MDA) of ivermectin, the principal strategy, to be discontinued in at least one focus in 34 countries by 2030 [1]. Considerable progress has been made, notably in the six Central and South American countries where around 0.56 million people had been at risk [2–7] and in the endemic areas in Sudan and Yemen where around 2.1 million people live [8].

The challenges for elimination are greatest in sub-Saharan Africa. These include, but are not limited to, the presence of more than 250 million people in areas where the parasite can be transmitted and the large size and hyperendemicity of many endemic areas [8–12]. The African Programme for Onchocerciasis Control (APOC; 1995–2015 [13]) concluded that in many areas an alternative treatment strategy (ATS) is required. One ATS identified is the mass administration of moxidectin [1, 14, 15].

The development of moxidectin for filarial diseases was initiated in the mid-1990s by the UNICEF/UNDP/World Bank/WHO Special Programme for Research and Training in Tropical Diseases (WHO/TDR) in consultation with and with the support of the Onchocerciasis Control Programme in West Africa (OCP; 1974–2002) and APOC [16–27]. Following analysis of the results of the Phase﻿ 3 study in *O. volvulus*-infected individuals, external advisory committees (one advising WHO/TDR; one advising APOC; the APOC Technical Consultative Committee) recommended that WHO/TDR identify an organisation to register moxidectin with a stringent regulatory authority and ensure moxidectin provision to countries wanting to use it within their onchocerciasis elimination strategies. In 2014, WHO licensed all data at its disposal to the Australian not-for-profit pharmaceutical company Medicines Development for Global Health (MDGH). Leveraging the prospect of a Priority Review Voucher, MDGH raised the funds necessary to address all requirements for a New Drug Application (NDA) to the US Food and Drug Administration (FDA), including an additional Phase 1 pharmacokinetic (PK) and cardiac safety study [20]. In 2018, an 8 mg dose of moxidectin was approved by the US FDA for treatment of *O. volvulus*-infected individuals aged ≥ 12 years [28]. The approved formulation is a 2 mg tablet.

Three additional clinical studies will provide further data for WHO and countries to decide on inclusion of moxidectin in onchocerciasis elimination strategies:

- (i) A paediatric safety and PK study (MDGH-MOX-1006) in Ghana to provide data to identify a moxidectin dose for the treatment of 4- to 11-year-old children for further safety evaluation and to support decisions on regulatory approval for this paediatric population (https://www.clinicaltrials.gov/study/NCT03962062, PACTR201907565746388; for protocol see: https://mox4oncho-multimox.net/resources).
- (ii) A recently completed randomised, double-blind, single-dose study (MDGH-MOX-3002) in ≥ 4-year-old individuals with ≥ 0 skin microfilariae density (microfilariae/mg skin [SmfD]). The study obtained safety data in 5550 adults, 2289 adolescents (age group: 12–17 years) and 187 children (age group: 4–11 years) in the Democratic Republic of the Congo (DRC) randomised 4:1 to moxidectin:ivermectin, and in 3240 adults, 890 adolescents and 840 children in Côte d’Ivoire randomised 4:1 to moxidectin:ivermectin with concomitant administration of 400 mg albendazole (https://www.clinicaltrials.gov/study/NCT04311671, PACTR202003567524647; for protocol see: https://mox4oncho-multimox.net/resources).
- (iii) A randomised, double-blind, ivermectin-controlled study (MDGH-MOX-3001) assessing the effect of three annual or five biannual administrations of a single dose of moxidectin or ivermectin in individuals aged ≥ 12 years with ≥ 10 SmfD is ongoing in DRC (https://www.clinicaltrials.gov/study/NCT0387s6262, PACTR202004639229710; for protocol see: https://mox4oncho-multimox.net/resources),

We report here the safety and PK data obtained in the paediatric study that resulted in 4- to 7-year-old and 8- to 11-year-old children receiving a moxidectin dose of 4 mg and 8 mg, respectively, in study MDGH-MOX-3002. The choice of the lower age limit was based on a recommendation of the APOC Technical Consultative Committee for inclusion of 4-year-old children in onchocerciasis control with moxidectin [29].

## Methods

### Trial registration

The trial was registered on Clinicaltrials.gov (ID NCT03962062) on 22 May 2019 and on the Pan African Clinical Trials Registry (ID PACTR201907565746388) on 15 July 2019.

### Regulatory agency and ethics committee approval

The protocol, information documents with informed assent and consent forms for potential participants and their parents/guardians as well as study conduct were approved by the Research Ethics Committee of the University of Health and Allied Sciences School of Public Health (UHAS), the Ethics Review Committee of the Ghana Health Service, the Ghana Food and Drugs Authority (GFDA) and the WHO Ethics Review Committee.

The UHAS Research Centre (UHAS RC) physicians, nurses, laboratory and data entry/management staff who conducted the study underwent Good Clinical Practice training by the GFDA as per GFDA requirements.

### Study design, objectives and sample size

This was a prospective, adaptive, open-label, single-dose PK and safety study of moxidectin in 4- to 17-year-old individuals. Participants were enrolled in age-based cohorts: Cohort I, adolescents aged 12 to 17 years; Cohort II, children aged 8 to 11 years; Cohort III, children aged 4 to 7 years.

The primary objective was to identify a moxidectin dose for 4- to 11-year-old children which provides comparable exposure distributions to those identified in adults and adolescents receiving the US FDA-approved dose.

The secondary objective was to evaluate the safety and PK of a single moxidectin dose in children and adolescents aged 4 to 17 years. While a single dose of 8 mg moxidectin is approved by the US FDA for the treatment of individuals aged ≥ 12 years, PK data for adolescents were not available at the time. These data were needed to optimise the population pharmacokinetic (popPK) model to achieve the primary objective.

Nine participants per cohort and moxidectin dose was considered to be an adequate sample size for full characterisation of moxidectin PK based on the PK data from the Phase 1 and 2 studies [19–23, 25].

### Eligibility criteria

To be included in the study, 4- to 17-year-old individuals living in an area designated by WHO as onchocerciasis endemic had to have provided informed assent together with parental/guardian consent and be able to stay at the UHAS RC. Girls of childbearing potential had to have committed to using contraception (included in local family planning guidelines with < 1% failure rate when correctly used) from pre-treatment (day 0) until approximately 6 months post-treatment. *Onchocerca volvulus* infection was not an inclusion criterion given no data supporting the assumption that *O. volvulus* infection impacts moxidectin PK and the lack of reliable diagnostics.

Exclusion criteria were chosen primarily to: (i) minimise risk, including history of serious medical or psychiatric condition, known or suspected concurrent clinically significant disease, infections, clinically-relevant laboratory abnormalities, known or suspected hypersensitivity to macrocyclic lactones or moxidectin tablet excipients, inability to swallow the tablets (flat oval, 8.0 × 4.5 × 3.0 mm), weight below specified limits, pregnancy or breastfeeding; (ii) minimise confounding of adverse event (AE) assessment, such as known or suspected concurrent clinically-significant disease, treatment with other investigational products, anti-helminthic drugs or vaccination within defined time periods before dosing; (iii) ensure that all planned assessments could be done. Details on the exclusion criteria are available in Additional File 1: Section 1.

### Ethical considerations

#### Selection of communities for recruitment

Communities for recruitment (Wii, Jagri-Do, Azua) were selected among those in the Kpassa subdistrict of the Nkwanta North district of Ghana where the principal investigator (PI; author NOO) had already conducted studies, including the moxidectin Phase 3 study. Thus, parents/guardians themselves, or people they and potential participants could talk to, were likely to have first-hand experience with research study participation, study procedures, moxidectin treatment and the UHAS RC (at the time of the Phase 3 study, the Onchocerciasis Chemotherapy Research Centre). Compared with individuals in a ‘research naïve’ village, these parents/guardians and potential participants therefore had a better basis for understanding the information about the study.

#### Community engagement, provision of information about the study and informed assent with parental/guardian consent

After the study had been presented to the Municipal Assembly (which conducts government business at the local level), the chiefs, elders and/or opinion leaders were informed about the study and the plans to obtain informed assent and consent. Upon their agreement, interested community members were informed about the study in community meetings. During the community meetings, each community selected a ‘community coordinator’ to serve as a link between study participants and their parents/guardians and the study team and proposed individual(s) as impartial witness(es) to the discussions between potential study participants and their parents/guardians and the study team about the study.

Study presentation and discussion in the community meetings were followed by discussions with parents/guardians and their child(ren) who approached the study team. This discussion took place in the presence of a literate independent witness chosen by the community. In these discussions, the study was presented using information documents in the local language and written in view of the different levels of understanding to be expected in the 4- to 17-year age range.

Informed assent was obtained from children aged ≥ 7 years. As per the commentary to the Council for International Organizations of Medical Sciences (CIOMS) Guideline 17 [30], children aged 4 to 6 years were observed by their parents/guardians and the study team member for expression of ‘deliberate objection’ while being told about the study. Parents/guardians provided informed consent only after the child had provided assent (or shown no expression of ‘deliberate objection’). Details about how the information process took into account the local culture and the fact that potential participants as well as their parents/guardians are, from a research ethics perspective, vulnerable populations are provided in Additional File 1: Section 2. The information documents are available in Additional File 1: Sections 3–6.

The results of the study as well as the fact that the results support evaluating the safety of moxidectin in a large number of children aged 4 to 11 years were shared in community meetings. At least one parent/guardian of each participant attended the meeting, as did interested participants and other community members.

#### Study implementation

The UHAS RC on the campus of the Volta Regional Hospital was at the time of the study approximately a 5-h drive from the Kpassa subdistrict. To minimise burden on potential participants and their parent(s)/guardian(s), all eligibility criteria not requiring medical or laboratory examinations were evaluated in the village (subsequently referred to as ‘pre-screening’). Potential participants who passed pre-screening were brought in groups to the UHAS RC, each accompanied by one parent/guardian. Unless a child/adolescent was found to be ineligible during the UHAS RC-based screening, they stayed with the accompanying parent/guardian until 7 days after moxidectin administration and were brought back to the UHAS RC for follow-up 14 and 28 days as well as 12 and 24 weeks after moxidectin administration on day 0. The accompanying parent/guardian was present during each study procedure. The results of the examinations were shared with the participant and accompanying parent/guardian as they became available.

The UHAS RC includes wards with a nurses’ station. During the night, two nurses were present in the ward and one physician was on call. The UHAS RC also includes an indoor dining/recreational area and an outdoor recreational area. Recreational activities offered included television, as well as local board and card games. The floor plan, including the full capacity and the reduced capacity for study conduct during the SARS-CoV-2/COVID-19 public health emergency, is shown in Additional File 1: Section 7.

A teacher hired for this study assembled school-age children by age group and taught them based on knowledge gaps identified in the group.

Parent/guardian health care needs becoming evident during their stay at the UHAS RC were addressed by UHAS RC staff, if possible, or else through referral to the Volta Regional Hospital.

In each community, a community coordinator (CC) organised study participants for all study activities at the community level, supported communication between participants/their parents and or guardians and study staff and facilitated, whenever needed, transport to the closest health facility. The CCs had been chosen by each community for previous studies and thus proven their ability to meet community, participant and investigator expectations. The CCs underwent study-specific training at the UHAS RC and were provided with everything required to fulfil their role (e.g. mobile phone units, writing materials, rain boots and coats, torch lights).

### Study evaluations

Screening evaluations included medical and medication history, physical examination, vital signs, body weight, height, upper arm circumference, 12-lead electrocardiograms (ECGs), haematology (haemoglobin, haematocrit, red blood cell count and morphology, white blood cell and differential white blood cell count, platelet count), clinical chemistry (aspartate-aminotransferase, alanine-aminotransferase, gamma-glutamyltransferase, amylase, creatine kinase, total protein, albumin, direct and total bilirubin, sodium, potassium, chloride, bicarbonate, phosphorus, blood urea nitrogen, creatinine), screening for co-infections (HIV, hepatitis B, and hepatitis C and others as clinically indicated [e.g. malaria]) and a serum pregnancy test for girls of child-bearing potential.

Post-treatment evaluations included targeted physical examination (i.e. informed by concurrent conditions, signs, symptoms and AEs), vital signs, haematology and clinical chemistry and 12-lead ECGs at the times required by the protocol (Additional File 1: Section 8) or as clinically indicated.

### Dose selection and administration

**Fig. 1:**
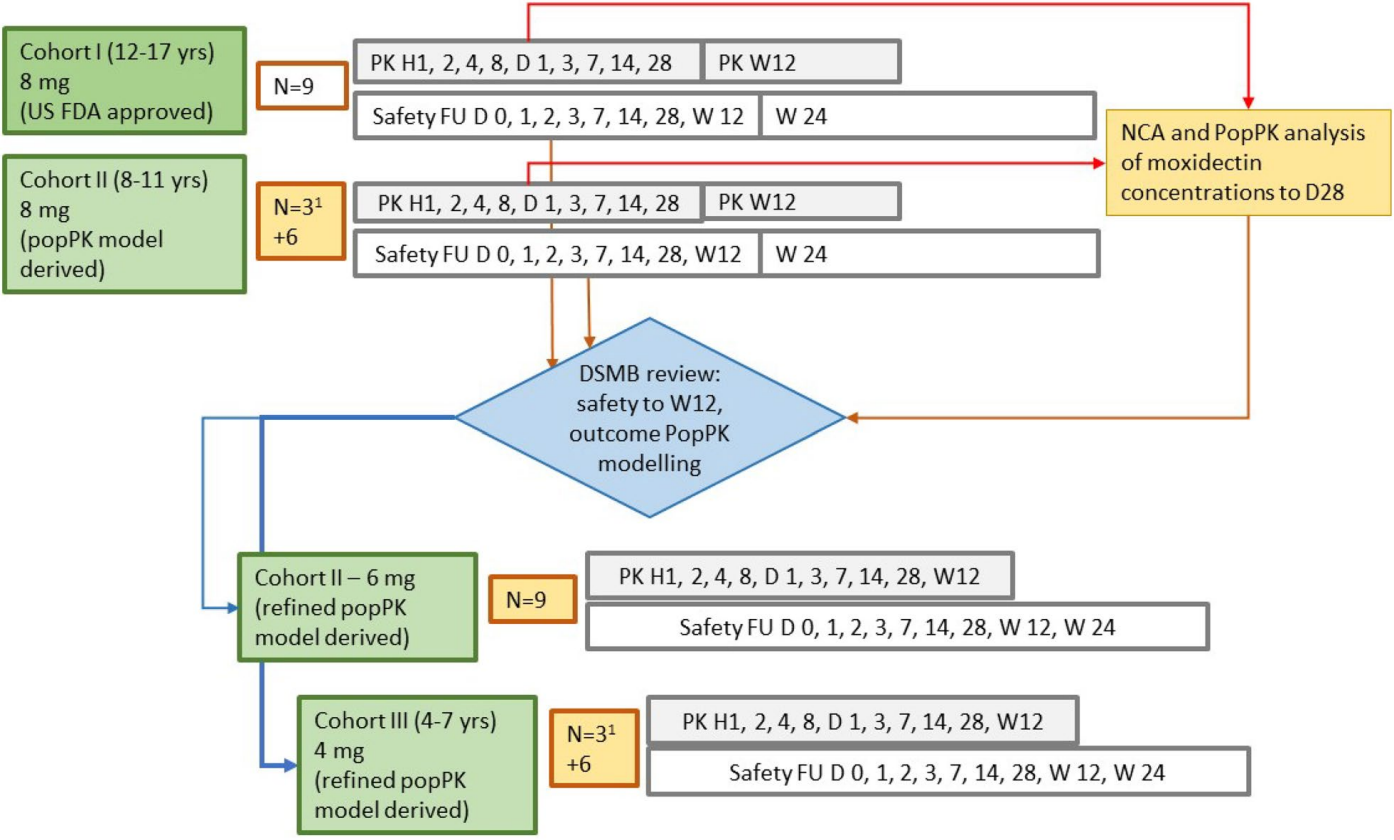
Overview of treatment, follow-up and basis for dose selection. ^1^Cohort II–8 mg and Cohort III were treated in 2 groups: treatment of a sentinel group of 3 children was followed by treatment of the remaining 6 children after the principal investigator had assessed the safety in the sentinel group up to and including day 3 as not raising any concerns. D, Day; DSMB, Data Safety Monitoring Board; H, hour; NCA, non-compartmental analysis; PK, pharmacokinetics; PopPK, population pharmacokinetics; safety FU, safety follow-up evaluations including targeted physical examinations, vital signs, 12-lead electrocardiograms (ECGs), adverse event assessment; W, week

Figure 1 provides an overview of the treatment, follow-up and dose selection.

Cohort I received the 8 mg dose approved by the US FDA for ≥ 12-year-old individuals.

An 8 mg dose was selected for Cohort II prior to study start based on popPK modelling and simulation of moxidectin concentration–time profiles in 8- to 11-year-old children receiving different doses of moxidectin and comparison of the exposure parameters (area under the curve [AUC] and maximum plasma concentration [C_max_]) with exposures in healthy male volunteers who had received single doses of up to 36 mg and in *O. volvulus*-infected adult women and men who had received an 8 mg dose of moxidectin. The popPK model had been built using the data from three single-dose Phase 1 studies in healthy adults [20, 21, 31] and the single-dose Phase 2 study in *O. volvulus*-infected participants [25]. Further information is provided in Additional File 1: Section 9. The simulations predicted that the AUC_0-∞_ (AUC from before dosing to infinity) in 8- to 11-year-old children (and adolescents) after an 8 mg dose would be in the range observed in adults receiving this dose. For C_max_, the upper limit of the predicted range was higher than that in adults but well below that in healthy males who had taken 36 mg moxidectin [20].

Analysis of the moxidectin concentrations in PK samples from Cohorts I and II to day 28 post-dose showed that the model overestimated apparent clearance (CL/F) in adolescents and children. Further analysis showed that this was attributable to their body weight being lower than that of adults. The model was updated to improve the way the effect of body weight was taken into account: fixed theoretical exponents (0.75 for clearance, 1.0 for volume terms) were replaced with allometric exponents on CL/F, the central volume (V_c_/F) and peripheral distribution (CL_d2_/F, V_p2_/F) estimated from the data. This corrected the overestimate of CL/F and resulted in a good fit of model-predicted and observed data. The updated model predicted that a 6 mg dose administered to 8- to 11-year-old children and a 4 mg dose administered to 4- to 7-year-old children would provide exposures within the range found in adults and adolescents after an 8 mg dose. Further details are provided in Additional File 1: Section 9.

The results of the analysis of the moxidectin concentration data and the safety data in Cohorts I and II up to 28 days and at least 12 weeks post-treatment, respectively, were reviewed by the Data Safety Monitoring Board, which recommended treatment of a second Cohort II with 6 mg and of Cohort III with 4 mg moxidectin.

After an overnight (> 10 h) fast, participants swallowed the required number of 2 mg moxidectin tablets with 250 ml water under supervision by the pharmacist and nurses, who checked that all tablets had been swallowed. Breakfast was provided 2 h later after the 2-h blood sample had been obtained. Date and time of moxidectin administration were recorded in the database.

### Evaluation of safety

Any unfavourable and unintended signs and symptoms which appeared or increased in severity after moxidectin administration, whether identified during study evaluations or based on information from the participant or their parent/guardian, were entered as an AE into the database. The PI was requested to attempt to establish a diagnosis based on signs and symptoms and document this diagnosis as an AE, rather than the individual signs/symptoms. AE characterisation included start and stop dates by severity (grade 1, mild; grade 2, moderate; grade 3, severe; grade 4, potentially life-threatening; grade 5, death related to an AE), according to the Division of AIDS Table for Grading the Severity of Adult and Pediatric Adverse Events, Version 2.1, July 2017 (https://rsc.niaid.nih.gov/clinical-research-sites/daids-adverse-event-grading-tables) and whether the AE was considered serious (i.e. resulted in death; a life-threatening situation; inpatient hospitalisation or prolongation of existing hospitalisation; persistent or significant disability/incapacity; or in a congenital anomaly/birth defect in the offspring of a woman who had received moxidectin). The probability of a causal relationship to moxidectin was assessed by the PI as unrelated, unlikely, possible, probable or definite according to protocol-specified criteria (Additional File 1: Section 10).

### Moxidectin plasma concentration measurement

A 1.5 ml sample of blood was collected in 2 ml potassium EDTA vacuum tubes during screening, and at 1, 2, 4, 8, 24 and 72 h, 7, 14 and 28 days and 12 weeks after dosing on day 0. The tubes were immediately put on crushed ice and centrifuged not later than 10 min after the blood draw at 2000* g* for 10 min at 4 °C. To minimise the number of times a needle needed to be inserted, a catheter was put in place before the 1 h time point and removed after the 8 h blood draw. The plasma was divided into two 250 µl aliquots and frozen at – 20 °C. One aliquot was sent on dry ice to the analytical laboratory (Frontage Laboratories, Exton, Philadelphia, USA) for moxidectin concentration measurement with the fully validated liquid chromatography with tandem mass spectrometry (LC/MS/MS) method used previously [20]. The second aliquot was retained at UHAS RC and destroyed after confirmation that a back-up sample was not needed. Date and time of sample collection were recorded in the database.

### Data analysis

#### Data management

Data were entered into the database at UHAS RC and, following study team internal source data verification and quality control, monitored both remotely and on-site by MDGH.

#### Non-compartmental analysis

The PK parameter estimates were obtained using non-compartmental methods from 396 moxidectin concentrations (11 concentrations from each of the 36 individuals dosed) and sampling times relative to the time of moxidectin administration. Concentrations < 0.1 ng/ml, the limit of quantitation, (5/9 week 12 samples from Cohort I, 6/9 week 12 samples from Cohort II—8 mg, 7/9 week 12 samples from Cohort II—6 mg, 9/9 week 12 samples from Cohort III) were considered to be zero for the PK parameter calculation. Summary statistics of moxidectin plasma concentrations were calculated only for time points with ≥ 50% of values above the limit of quantitation. Phoenix™ WinNonlin (Version 8.3.5), validated by the Certara software development team (Radnor, PA, USA), was used for these analyses. Further details are provided in Additional File 1: Section 11.

#### Safety analysis

The AE verbatims were coded using the Medical Dictionary for Regulatory Activities (MedDRA) Version 25.0. Descriptive statistics of AEs by system organ class and preferred term, were generated using SAS (Version 9.4 or above; SAS Institute Inc., Cary, NC, USA).

## Results

The study was conducted from 29 March 2021 (date of first assent/consent) to 28 September 2022 (date of last follow-up visit). Among the 66 adolescents/children screened, 36 were eligible, treated and completed the study. Screen failure reasons included clinically relevant laboratory abnormalities (n=11), infections (n=8), known or suspected concurrent clinically significant conditions (n=6), weight below the protocol-specified limit (n=3) and two with siblings already enrolled.

### Baseline characteristics

Table 1 summarises the demographic characteristics of the participants. In Cohort I, 44.4% were girls/women, while in Cohorts II and III, 61.1% and 66.7% were girls, respectively. Body weight ranged from 30.8 to 55.4 kg in Cohort I, from 19.4 to 36.8 kg in Cohort II and from 13.6 to 20.6 kg in Cohort III.

**Table 1:** Demographic characteristics of the participants

Demographic characteristics	Cohort I (12 to 17 years)	Cohort II (8 to 11 years)			Cohort III (4 to 7 years)	Total (*N* = 36)
Dose (number participants dosed)	8 mg (*N* = 9)	8 mg (*N* = 9)	6 mg (*N* = 9)	6 or 8 mg (*N* = 18)	4 mg (*N* = 9)	
Parameter/statistic						
* Age (years)*						
Mean ± SD	13.7 ± 1.66	9.7 ± 0.87	9.1 ± 1.05	9.4 ± 0.98	5.4 ± 1.33	9.5 ± 3.19
Median (Q1; Q3)	14.00 (12.0;14.0)	10.00 (9.0; 10.0)	10.00 (8.0; 10.0)	10.00 (8.0; 10.0)	5.00 (4.0; 7.0)	10.00 (7.5; 11.5)
Min; Max	12; 17	8; 11	8; 10	8; 11	4; 7	4; 17
*Sex, n (%)*						
Male	5 (55.6)	4 (44.4)	3 (33.3)	7 (38.9)	3 (33.3)	15 (41.7)
Female	4 (44.4)	5 (55.6)	6 (66.7)	11 (61.1)	6 (66.7)	21 (58.3)
*Height (cm)*						
Mean ± SD	149.74 ± 10.318	133.11 ± 10.590	130.09 ± 9.307	131.60 ± 9.795	108.81 ± 7.922	130.44 ± 17.381
Median (Q1; Q3)	147.50 (142.7; 152.5)	131.50 (125.0; 141.0)	132.00 (120.2; 139.0)	131.75 (124.6; 139.6)	110.50 (102.0; 113.9)	131.75 (118.6; 142.4)
Min; Max	135.0; 170.0	118.6; 150.5	118.5; 141.5	118.5; 150.5	96.5; 120.0	96.5; 170.0
*Mean upper arm circumference (cm)*						
Mean ± SD	21.78 ± 2.065	18.66 ± 1.591	18.42 ± 0.981	18.54 ± 1.288	15.68 ± 1.347	18.63 ± 2.644
Median (Q1; Q3)	21.1 (20.1; 23.0)	18.6 (17.3; 20.0)	18.5 (17.7; 19.0)	18.6 (17.4; 19.5)	15.4 (15.1; 15.6)	18.6 (17.0; 20.1)
Min; Max	20.0; 25.4	16.7; 21.1	17.3; 20.1	16.7; 21.1	14.1; 18.5	14.1; 25.4
*Weight (kg)*						
Mean ± SD	39.42 ± 8.754	27.13 ± 4.844	25.89 ± 4.567	26.51 ± 4.612	16.90 ± 2.662	27.34 ± 9.766
Median (Q1; Q3)	34.80 (33.8; 41.8)	24.40 (23.6; 29.4)	25.80 (22.6; 30.2)	25.10 (22.8; 30.2)	15.60 (15.0; 19.5)	25.10 (20.5; 31.7)
Min; Max	30.8; 55.4	22.6; 36.8	19.4; 31.4	19.4; 36.8	13.6; 20.6	13.6; 55.4
*Body mass index (kg/m)*^*2*^						
Mean ± SD	17.39 ± 1.745	15.23 ± 1.120	15.19 ± 1.068	15.21 ± 1.062	14.20 ± 0.919	15.50 ± 1.683
Median (Q1; Q3)	17.10 (15.9; 18.6)	15.70 (14.6; 16.1)	15.10 (14.6; 15.8)	15.30 (14.6; 16.1)	14.20 (14.0; 14.9)	15.15 (14.5; 16.3)
Min; Max	15.1; 20.2	13.3; 16.5	13.4; 16.9	13.3; 16.9	12.3; 15.2	12.3; 20.2

*Max* Maximum, *Min* minimum,* Q1, Q2* first and third quartile, *SD* standard deviation

All participants in Cohort I, 8/18 participants in Cohort II and 4/9 participants in Cohort III had a history of health problems. Medical history included gastrointestinal disorders (abdominal pain, diarrhoea, vomiting), nervous system disorders (headache, dizziness), musculoskeletal disorders (arthralgia, backpain, musculoskeletal chest pain and pain in an extremity), skin disorders (pruritus and rash), eye pruritus, cough and nosebleeds, anaemia, hypoacusis, a furuncle and decreased appetite.

### Safety of moxidectin

Tables 2 and 3 provide an overview of AEs and the types of AEs which occurred in at least two participants, respectively.

**Table 2:** Overview of adverse events by seriousness, relationship to moxidectin, severity and outcome

Adverse events	Cohort I–8 mg (12 to 17 years), *N* = 9	Cohort II–8 mg (8 to 11 years), *N* = 9	Cohort II–6 mg (8 to 11 years), *N* = 9	Cohort III–6 mg (4 to 7 years), *N* = 9	Total (*N* = 36)
	*n* (%) m^a^	* n* (%) m^a^	*n* (%) m^a^	* n* (%) m^a^	*n* (%) m^a^
All AEs	4 (44.4) 4	6 (66.7) 10	7 (77.8) 11	7 (77.8) 13	24 (66.7) 38
Serious AEs	0	0	0	0	0
AEs considered to be moxidectin-related	0	0	0	0	0
*AEs by severity*					
Grade 1-mild	3 (33.3) 3	6 (66.7) 9	6 (66.7) 10	6 (66.7) 10	21 (58.3) 32
Grade 2-moderate	1 (11.1) 1	1 (11.1) 1	1 (11.1) 1	2 (22.2) 1	5 (13.9) 4
Grade 3-severe	0 (0.0) 0	0 (0.0) 0	0 (0.0) 0	1 (11.1) 2	1 (2.8) 2
*AE Outcome*					
Recovered/resolved	4 (44.4) 4	6 (66.7) 10	7 (77.8) 11	7 (77.8) 13	24 (66.7) 38

Differences relative to the results posted on Clinicaltrials.gov are due to the fact that the results posted include a ‘false positive investigation result’

*AE* Adverse event, *N* number of participants treated

^a^*n* is the number of participants with at least one AE in the specified category; m is the number of AEs in the specified AE category

**Table 3:** Type of adverse events reported by at least two participants

MedDRA preferred term of AEs in ≥ 2 participants	Cohort I–8 mg (12 to 17 years), *N* = 9	Cohort II–8 mg (8 to 11 years), *N* = 9	Cohort II–6 mg (8 to 11 years), *N* = 9	Cohort III–6 mg (4 to 7 years), *N* = 9	Total (*N* = 36)
	*n* (%) m ^a^	* n* (%) m ^a^	* n* (%) m ^a^	* n* (%) m ^a^	*n* (%) m ^a^
Malaria	1 (11.1) 1	4 (44.4) 6	5 (55.6) 7	4 (44.4) 6	14 (38.9) 20
Upper respiratory tract infection	1 (11.1) 1	1 (11.1) 1	1 (11.1) 1	0	3 (8.3) 3
Conjunctivitis	0	1 (11.1) 1	1 (11.1) 1	0	2 (5.6) 2
Abdominal pain	0	2 (22.2) 2	0	1 (11.1) 1	3 (8.3) 3
Diarrhoea	0	0	2 (22.2) 2	1 (11.1) 1	3 (8.3) 3

*AE* Adverse event, *N* number of participants treated

^a^*n* is the number of participants with at least one AE in the specified category; m is the number of AEs in the specified AE category

No participant experienced a serious AE (SAE). All AEs were assessed as unrelated to moxidectin. Twenty-four participants experienced a total of 38 AEs, all of which resolved.

The highest level of severity reported was grade 3. Both grade 3 AEs occurred in a 4-year-old girl: anaemia was diagnosed at the last scheduled study visit 188 days post-dose, which resulted in a test for intestinal helminths and a subsequent diagnosis of hookworm infection. The child was treated by the study staff with a single dose of 400 mg albendazole and also took iron III polymaltose 5 ml once daily for 19 of the 28 days prescribed. At the follow-up visit 33 days after diagnosis, haemoglobin levels were normal and the hookworm test was negative.

Malaria was the AE diagnosed and reported most frequently in the study. Three 8- to 11-year-old and three 4- to 7-year-old children had more than one malaria episode.

AEs diagnosed in only one participant were limb abscess, anaemia with hookworm infection, tinea versicolour, vomiting, skin laceration and neck pain. Additional File 1: Section 11 shows all AEs by cohort and dose.

### Moxidectin plasma concentrations and PK parameter estimates

**Fig. 2:**
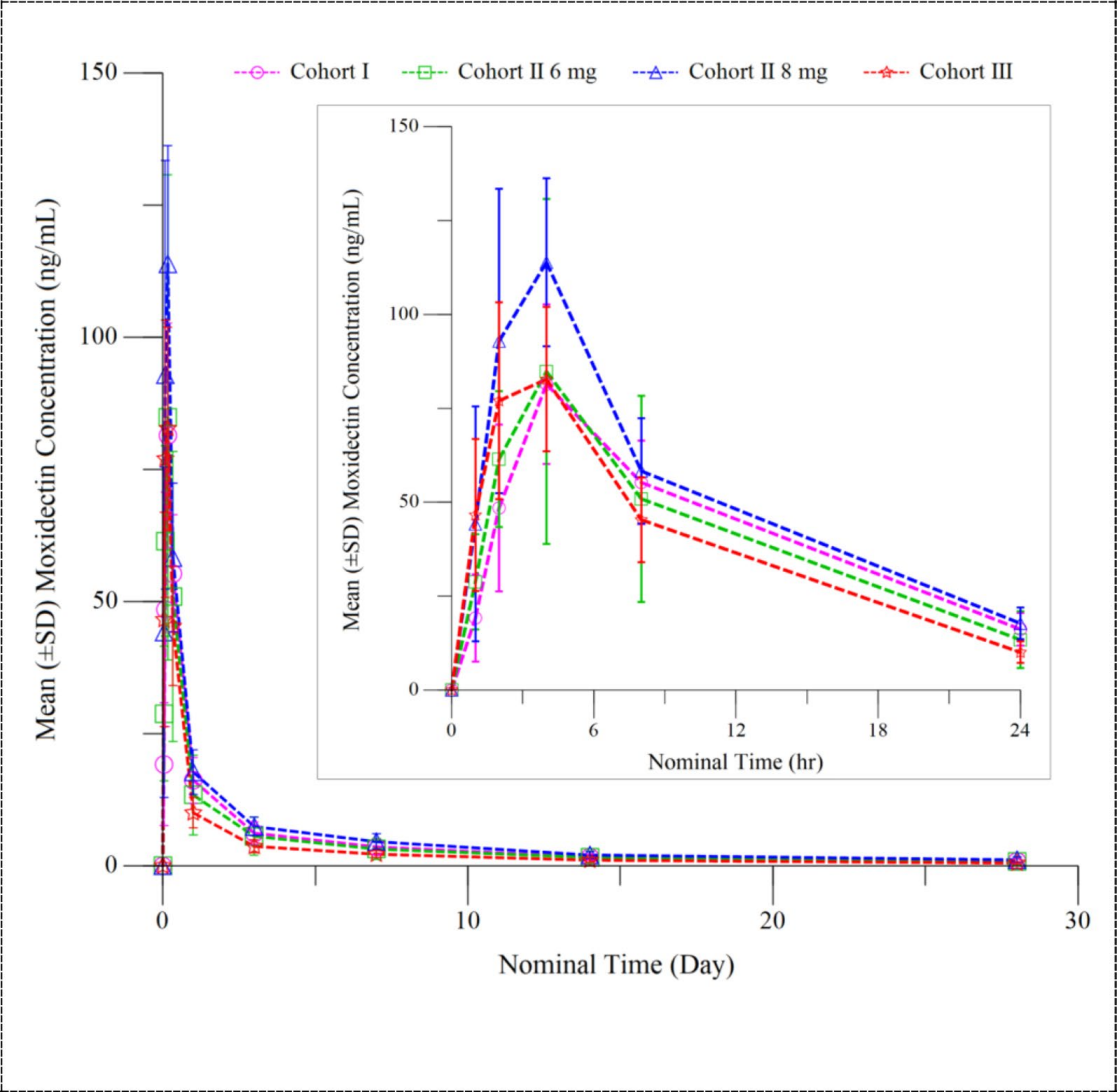
Mean (± SD) moxidectin plasma concentration–time profiles for Cohort I– 12 to 17 years, Cohort II–8 to 11 years and Cohort III– 4 to 7 years. Mean concentrations at 12 weeks post-dose were not calculated because moxidectin concentration was above the limit of quantitation for only 4/9 Cohort I participants, 3/9 Cohort II participants after administration of 8 mg moxidectin and 2/9 Cohort II participants after administration of 6 mg moxidectin. SD, Standard deviation

Figure 2 shows the mean moxidectin plasma concentration time profiles and Table 4 presents the C_max_ and AUC_0-∞_ estimates for Cohorts I to III and for healthy adult males and *O. volvulus*-infected adults. Summary statistics of moxidectin plasma concentrations and of all other PK parameters are provided in Additional File 1: Section 11

**Table 4:** Maximum moxidectin plasma concentration and area under the moxidectin concentration–time curve from pre-treatment to infinity

Study population	Dose (n)	C_max_ (ng/ml) AM ± SD (range)	AUC_0-∞_ (ng h/ml) AM ± SD (range)
Cohort I—12 to 17 years	8 mg (9)	84.6 ± 18.2 (67.0—129)	3230 ± 868^e^ (2490—4780)^e^
Cohort II—8 to 11 years	8 mg (9)	118 ± 26.5 (70.7—149)	3910 ± 1070 (2280—5760)
Cohort II—8 to 11 years	6 mg (9)	89.0 ± 43.4 (59.1—187)	2920 ± 2120 (1220—8270)
Cohort III—4 to 7 years	4 mg (9)	89.4 ± 24.1 (55.9—131)	2070 ± 587 (1330—2980)
*O. volvulus*-infected adults^a^	2 mg (33)	16.6 ± 4.1 (10.6—24.8)	690 ± 301 (331—1723)
*O. volvulus*-infected adults^a^	4 mg (34)	34.3 ± 6.6 (21.9—50.3)	1169 ± 488 (485—2827)
*O. volvulus*-infected adults^a^	8 mg (31)	63.1 ± 20.0 (34.6—114.9)	2738 ± 1606 (1349—9725)
Healthy males^b^	36 mg (5)	289 ± 52 (231.9—331.5)	10,835 ± 2587 (7130—13,116)
Healthy males^c^	36 mg (5)	296 ± 47 (230.9—342.1)	14,972 ± 2233 (13,099—17,760)
Healthy males^a, d^	36 mg (10)	252 ± 50.3 (184—348)	NA

Data reported in publications were recalculated based on raw data to obtain comparable units or obtained from study reports, as required

*AM* Arithmetic mean,* AUC*_*0-∞*_ area under the concentration–time curve from time zero (pre-dosing) to infinity post-dosing,* C*_*max*_ maximum observed plasma concentration, *SD* standard deviation

^a^Tablet formulation fasted [25], additional information on C_max_ and AUC_0-∞_ relative to efficacy measures [17] is available in Additional File 1: Section 12

^b^Liquid formulation fasted [19]

^c^Liquid formulation fed [19]

^d^NA AUC_0-∞_ not available. AM and range for AUC_0-Day22_ for 7/10 volunteers treated was 7350 ± 1795 and 4602—10,197 [20]

^e^*n* = 8

In all cohorts, the median time to maximum plasma concentration (T_max_) was 4.0 h (range 1.9 to 4.1 h, with the exception of one adolescent who achieved C_max_ only 8 h after dosing). These values are comparable to the T_max_ reported for *O. volvulus*-infected adults who received a single dose of 2, 4 or 8 mg moxidectin (2.3–4.1 h, range 1.2–4.2 h, [25]). The arithmetic mean (AM) (± standard deviation) elimination half-life ranged from 17.3 ± 10.29 days in Cohort I to 8.5 ± 1.8 days in Cohort III compared to a range of 17.7 ± 8.6 to 23.2 ± 21.9 days reported for *O. volvulus*-infected adults [25]. In all Cohorts, mean C_max_ was above that in *O. volvulus*-infected adults receiving 8 mg moxidectin (Table 4). The mean AUC_0-∞_ in Cohorts I, II-8 mg and II-6 mg was above that in *O. volvulus*-infected adults receiving 8 mg. In contrast, the mean AUC_0-∞_ in Cohort III was below that of *O. volvulus*-infected adults after an 8 mg dose but above that of *O. volvulus*-infected adults after receiving a 4 mg dose (Table 4).

## Discussion

This study identified no safety concerns in 8- to 11-year-old children after a 6 mg or 8 mg dose of moxidectin (*n* = 9 children for each dose), or in 4- to 7-year-old children after a  4 mg dose of moxidectin (*n* = 9). None of the observed AEs were considered to be moxidectin-related, and all AEs reflected events typically seen in the study population without treatment (Tables 2, Table 3).

The primary objective of this study was to identify a dose for 4- to 11-year-old children which provides exposures comparable to those identified in adults and adolescents receiving an 8 mg dose that resulted in an efficacy and safety profile in *O. volvulus*-infected adults and adolescents which supported US FDA approval [17, 18, 26, 27]. Both, mean C_max_ and AUC_0-∞_ were higher in adolescents than in adults (Table 4), making exposure of adolescents the objective-relevant reference value. Mean C_max_ was higher in 8- to 11-year-old children after the 8 mg dose and the 6 mg dose as well as in 4- to 7-year-old children after the 4 mg dose than in adolescents receiving an 8 mg dose. C_max_ ranges overlapped. Compared to the AUC_0-∞_ in adolescents, mean AUC_0-∞_ was higher in 8- to 11-year-old children after the 8 mg dose but lower in 8- to 11-year-old children after the 6 mg dose and 4- to 7-year-old children after the 4 mg dose, with ranges overlapping between all four cohorts. The apparent age or cohort dependence of AUC is attributed to the impact of weight on moxidectin clearance. That impact had not been evident for the weight range of adults for whom moxidectin concentration data had been obtained previously. This impact emerged during the popPK model analysis of the moxidectin plasma concentrations from Cohorts I and II–8 mg and was further supported by inclusion of the data from Cohorts II—6 mg and III into the analysis (Additional File 1: Section 9).

The weight dependency of exposure suggests that doses for 4- to 11- year-old children resulting in exposures in the range of adolescents after an 8 mg dose could only be achieved with weight-based dosing. However, weight-based dosing is not practical during MDA. Provision of scales is a major cost-driver, and reliable calibration and use of scales cannot be guaranteed in the remote locations where MDA is needed. A case in point is the MDA of ivermectin: ivermectin was initially dosed by weight but this was changed to dosing by height [32, 33]. Therefore, the exposures in the paediatric cohorts were compared with other exposures across the wide therapeutic index demonstrated for moxidectin to inform the choice of doses for treatment of 4- to 11-year-old children in study MDGH-MOX-3002.

From a safety perspective, for both C_max_ and AUC_0-∞_, the upper range in 8- to 11-year-old children after an 8 mg dose and in 7-year-old children after a 4 mg dose was well below the lower range in adult healthy males after a 36 mg moxidectin dose (Table 4). Given that a dose of 36 mg, the highest administered to humans, was well tolerated [19, 20], this comparison indicates that doses of 8 mg and 4 mg for 8- to 11- and 4- to 7-year-old children, respectively, are unlikely to result in adverse reactions to moxidectin. Consideration also needs to be given to AEs in *O. volvulus*-infected individuals attributable to moxidectin’s microfilaricidal effect, i.e. the Mazzotti reactions which reflect the body’s immunological reaction to dying and dead microfilariae. Experience with the microfilaricidal drug diethylcarbamazine has shown that the severity of some Mazzotti reactions is a function of the pre-treatment microfilaridermia and thus the number of microfilariae killed [34, 35]. The Phase 2 study showed that a single dose of 8 mg moxidectin resulted in nearly complete elimination of microfilariae from the skin 8 days after treatment (mean reduction 99.3% [17], minimum reduction among participants with at least 5 microfilariae/mg skin pre-treatment 78%; Additional File 1: Section 12). Consequently, any higher microfilaricidal activity that might be associated with higher exposure in children than in adults or adolescents would increase the number of microfilariae killed only marginally. Thus, a worse safety profile in *O. volvulus*-infected 8- to 11-year-old children after an 8 mg dose and in 4- to 7-year-old children after a 4 mg dose than in *O. volvulus*-infected adults and adolescents after an 8 mg dose is unlikely.

From an efficacy perspective, the AUC_0-∞_ in 4- to 7-year-old children after a 4 mg dose was below that of *O. volvulus*-infected adults after an 8 mg dose. It was, however, above the AUC_0-∞_ in *O. volvulus*-infected adults after a 4 mg dose (Table 4). After a 4 mg dose, the mean SmfD reduction in *O. volvulus*-infected adults was between 98% and 100% of the pre-treatment SmfD from 8 days to 12 months after treatment and the SmfD were significantly lower than that in ivermectin-treated adults from 8 days to 18 months after treatment (*p* between 0.0035 and < 0.0001). Even after a 2 mg dose, the mean SmfD reduction was between 94% and 100% of pre-treatment SmfD from 8 days to 12 months after treatment, with the SmfD being significantly lower than that after ivermectin treatment from 8 days to 12 months after treatment (*p* between 0.003 and < 0.0001) [17]. This indicates that 4- to 7-year-old children receiving 4 mg moxidectin will benefit from moxidectin’s efficacy, both absolutely and compared to ivermectin treatment.

Based on these comparisons, 4 mg for 4- to 7-year-old children and 8 mg for 8- to 11-year-old children were selected to obtain additional safety data in study MDGH-MOX-3002. In November 2024, the Ghana Food and Drugs Authority approved MDGH’s Marketing Authorisation Application (product registry page on https://fdaghana.gov.gh/) which included these doses [36, 37].

A 6 mg dose for 8- to 11-year-old children was not selected based on the safety and exposure data obtained after an 8 mg dose and in consideration of operational and cost aspects for potential MDA with moxidectin. A two dose-level regimen (4 mg for 4- to 7-year-old children, 8 mg from age 8 years onward) is easier to implement reliably than a three dose-level regimen. It might also reduce the number of tablets per dose if higher strength moxidectin tablets become available.

MDGH is considering the development of a higher strength tablet for country onchocerciasis elimination programmes. A reduction in the number of tablets required per dose will both increase the supply capacity and reduce the cost of manufacturing and transport to and within countries. Pharmaceutical companies donating medicines for the control and elimination of tropical diseases [38] are for-profit companies and may benefit from tax deductions to reduce the cost of donations [39]. In contrast, MDGH is a not-for-profit pharmaceutical company. MDGH is working with other stakeholders currently supporting the elimination of onchocerciasis [40] and control and elimination of other neglected tropical diseases, including through the donation of medicines [41], to develop a sustainable financing mechanism. The objective is to ensure that at a minimum moxidectin can be provided to countries at the cost for manufacture and transport to a single port of entry per country.

## Conclusions

The safety data obtained in this study and the comparison of exposures with those of healthy volunteers after 36 mg moxidectin and of *O. volvulus*-infected adults after 8 mg and 4 mg moxidectin supported the selection of a 4 mg dose for 4- to 7-year-old children and an 8 mg dose for 8- to 11-year-old children. The recently completed study MDGH-MOX-3002 and future implementation research studies will provide large-scale safety data which will confirm the dose selection or drive evaluation of height-based dosing.

## Supplementary Information

Additional File 1: Sections 1-13

## Data Availability

Individual participant data will be made available by MDGH to researchers based on the outcome of MDGH review of requests for the data. Requests should include the objectives, data analysis plan and plans to obtain applicable Ethics Committee approvals and involve the investigators (UHAS, MDGH and TDR co-authors on this manuscript) as well as commitments to not share the data with anybody not named in the request and to open access publication of the results.

## Abbreviations

AE: Adverse event
AM: Arithmetic mean
AUC: Area under the concentration–time curve
AUC_0-∞_: Area under the concentration–time curve from before dosing to infinity
CIOMS: Council for International Organizations of Medical Sciences
CL/F: Apparent clearance
CL_d2_/F: Apparent inter-compartmental clearance between the central and second peripheral compartments
C_max_: Maximum plasma concentration
DRC: Democratic Republic of the Congo
DSMB: Data and Safety Monitoring Board
ECG: Electrocardiograms
GFDA: Ghana Food and Drugs Authority
MDA: Mass drug administration
MedDRA: Medical Dictionary for Regulatory Activities
PI: Principal Investigator
PK: Pharmacokinetics
popPK: Population pharmacokinetics
RC: Research Center
SAE: Serious adverse event
SmfD: Skin microfilariae density (mf/mg skin)
T_max_: Time of maximum plasma concentration
UHAS: University of Health and Allied Sciences, Ho, Volta Region, Ghana
FDA: US Food and Drug Administration (of the USA)
V_c_/F: Apparent volume of distribution of the central compartment
V_p2_/F: Apparent volume of distribution of the second peripheral compartment
WHO: World Health Organization
