# Supplementary material for: Identification of a moxidectin dose for 4- to 11-year-old children to support registration and potential use for onchocerciasis elimination: results of an open-label pharmacokinetic and safety study
Source: Parasit Vectors. 2025 Jul 24;18:295. doi: 10.1186/s13071-025-06891-z (PMC12291498; doi:10.1186/s13071-025-06891-z)
Supplement: Supplementary file 1 — Additional File 1: Sections 1-13 [file 13071_2025_6891_MOESM1_ESM.pdf]

Supplementary information to:

Opoku et al. Identification of a moxidectin dose for 4-to 11-year-old children to support registration and potential use for onchocerciasis elimination: Results of an open-label pharmacokinetic and safety study in an onchocerciasis endemic area in Ghana

Additional File 1 2

1. Exclusion criteria 2
2. Path to informed assent and parental/guardian consent 3
3. Information document for 12- to 17-year-old adolescents and their parents and guardians 5
4. Information document for parents and guardians of 4- to 11-year-old children 23
5. Information document for 7- to 11-year-old children 40
6. Information document for 4- to 6-year-old children and older children considered not mature enough to provide assent 46
7. Consulting room, Offices and study participant accommodation at the University of Health and Allied Sciences (UHAS) Research Centre 49
8. Overview of timing and evaluations conducted during screening and post-treatment follow-up 50
9. Population pharmacokinetic modelling for dose selection in the study and parameter estimates for the initial, the updated and the final model 51
    - Fig S 1: Individual moxidectin plasma concentrations in each adult in the studies which contributed to the development of the popPK model and in the participants in study MDGH-MOX-1006 52
    - Fig S 2: Schematic of the moxidectin PopPK model 53
    - Table S 1: Parameter estimates for the initial moxidectin PopPK model, the updated model and the final model after incorporation of all data 55
10. Additional information on evaluation of safety of moxidectin and results 57
    - Table S 2: Criteria for assessing the causal relationship of an adverse event (AE) to moxidectin administration 57
    - Table S 3: All adverse events recorded after moxidectin administration by system organ class and preferred term 57
11. Moxidectin plasma concentrations and pharmacokinetic parameters 58
    - Table S 4: Summary statistics of plasma moxidectin concentrations by cohort and time point 58
    - Fig S 3: Dose-normalised mean (± standard deviation) plasma moxidectin concentration-time profiles 59
    - Table S 5: Summary statistics of plasma moxidectin pharmacokinetic parameters 59
    - Table S 6: Abbreviations of plasma moxidectin pharmacokinetic parameters 61
12. Additional information on C_{max} and $AUC_{0-\infty}$ in *O. volvulus* infected adults relative to effect on *O. volvulus* skin microfilariae 62
    - Fig S 4: Measures of moxidectin efficacy vs. C_{max} and $AUC_{0-\infty}$ in *O. volvulus* infected adults with ≥5 mf/mg before administration of 2 mg (blue), 4 mg (red) or 8 mg (green). 63
13. References 64

Supplementary information to:

Opoku et al. Identification of a moxidectin dose for 4-to 11-year-old children to support registration and potential use for onchocerciasis elimination: Results of an open-label pharmacokinetic and safety study in an onchocerciasis endemic area in Ghana

Additional File 1

1. Exclusion criteria

Exclusion criteria were chosen primarily to minimise risk to participants (e.g. due to pre-existing conditions or other baseline characteristics (serious medical or psychiatric condition, known or suspected concurrent clinically significant disease, co-infections, clinical laboratory abnormalities, known or suspected hypersensitivity to macrocyclic lactones or excipients used in the formulation of moxidectin inability to swallow moxidectin tablets, weight below specified limits, pregnancy or breastfeeding), minimise confounding adverse event (AE) assessment (treatment with other investigational products, ant-helminthic drugs, vaccination within defined time periods before dosing) and ensure that all planned assessments could be done.

1. History of serious medical or psychiatric condition which, in the opinion of the investigator, would put the subject at increased risk by participating in the study or jeopardize study outcomes.
2. Known or suspected concurrent clinically significant renal, cardiac, pulmonary, vascular, metabolic (thyroid disorders, adrenal disease), immunological disorders or malignancy, congenital heart disease, chronic lung disease.
3. Co-infections with Hepatitis B, Hepatitis C, or human immunodeficiency virus (HIV), known or suspected malaria or other ongoing viral, bacterial, or plasmodium infection at Screening and/or Baseline; or *Loa loa* co-infection.
4. Clinically relevant laboratory abnormalities at Screening, including:
    - Haemoglobin < 9.5 grams per decilitre (g/dL);
    - Neutrophil (granulocyte) count < 1.5 x 10^9/L;
    - Platelet count < 110 x 10^9/L;
    - Alanine aminotransferase (ALT) > 1.5 times the upper limit of normal range (ULN);
    - Total bilirubin > 1.5 times ULN.
5. Treatment with an investigational product within 28 days, or 5 half-lives, of Baseline, whichever is longer, or with ivermectin or any other anti-helminthic treatments within 28 days of Baseline.
6. Vaccination within 7 days of Baseline.

7. Known or suspected hypersensitivity to macrocyclic lactones or excipients used in the formulation of moxidectin.
8. Weight:
   - Cohort I (12 to 17 years): < 30 kg;
   - Cohort II (8 to 11 years): < 18 kg;
   - Cohort III (4 to 7 years): < 12 kg.
9. For females of child-bearing potential, pregnant or breastfeeding, or planning to become pregnant.
10. Inability to swallow tablets (flat oval, 8.0 millimetres (mm) x 4.5 mm x 3.0 mm).
11. Poor venous access.
12. Unwilling, unlikely or unable to comply with all protocol specified assessments.
13. Previous enrolment in this study, or sibling of another child already enrolled in this study.

2. Path to informed assent and parental/guardian consent

After the study had been presented to the Municipal Assembly (which performs government business at the local level), the chiefs, elders, and/or opinion leaders were informed about the study and the plans to obtain informed assent and consent. Upon their agreement, interested community members were informed in community meetings. During the community meetings, each community selected a ‘community coordinator’ to serve as a link between study participants and their parents/guardians and the study team and proposed individual(s) as impartial witness(es) to the discussions between potential study participants and their parents/guardians and the study team about the study.

Four different information and/or consent/assent documents were used:

1. An information document for adolescents (12- to 17-years) and their parent(s)/guardian(s) including also the assent and consent forms written in language understandable to a 12-year-old child (see **SECTION** 3);
2. A document for parents of children aged 4- to 11-years (see **SECTION** 4), in language similar to that under 1. above including two consent/assent forms:
   i. one for parents/guardians and children <12 years who are providing assent (includes written parent/guardian consent and child assent); and
   ii. one for parents/guardians of children considered too immature to provide assent which included documentation of the assessment of expression of ‘deliberate objection’ by the child;

3. A much simpler information document for children aged 7- to 11-years considered mature enough to provide assent (see **SECTION** 5); and
4. An even simpler information document for children 4- to 6-years or older children considered too immature to provide assent in the joint assessment of the parent(s)/guardian(s) and the investigator (see **SECTION** 6).

Early on in the presentation and discussion with 4- to 6-year-old children, a 2 mg moxidectin placebo was shown and the child and their parent(s)/guardian(s) were asked whether they think the child can swallow such tablets. If a child or their parent(s)/guardian(s) indicated that swallowing would not be possible, there was no further discussion. If swallowing the tablet was not seen as a problem, the child was told about the need to take blood and venous access was assessed. If a child had poor venous access, there was no further discussion. Assessing these two eligibility criteria during the information process was considered ethically acceptable since it did not include an invasive procedure or confidential health information and spared the child and obtaining all other information about the study had no value for a child who was ineligible because they could not swallow the tablets or had poor venous access.

To ascertain to the extent possible that the potential participants and their parent(s)/guardian(s) had understood critical elements of the information shared and discussed with them, once they had told the investigator they wanted to participate / consent to participation of their child/ward, they were asked age-appropriate questions about key elements of the study. The relevant information was shared and discussed with them again if the answers indicated that this was needed.

Both parents or guardians (unless one was deceased, unknown, incompetent, or not reasonably available, or only one parent/guardian had legal responsibility for the care and custody of the child) of potentially eligible children had to provide written consent (via signature or thumbprint) in the presence of an independent literate witness. The reason for consent by only one parent/guardian had to be documented. Within the Ghanaian culture, guardians are family members who look after orphans or children of parents unable to take care of them for medical reasons.

3. Information document for 12- to 17-year-old adolescents and their parents and guardians

General Information About the Study

I am Dr..., a doctor working at the University of Health and Allied Sciences in Hohoe. We are testing a new medicine called moxidectin in children aged 4 to 17 years. Moxidectin is a new treatment for onchocerciasis (river blindness), which you call "*oncho*".

Testing new medicines is called doing "research" or "a study" and means we want to learn something about the new medicine.

Here, we explain to you and your parents or guardians what we already know about moxidectin, what will happen during the study and the risks and advantages of taking part in this study.

This will help you decide whether you want to take part in this study or not and it will help your parents or guardians decide whether they will allow you to take part or not.

If you do not want to take part or your parents or guardians don't want you to take part, please don't be afraid to tell us. Your care or the care of your parents or guardians at your local health care centers will not be affected by saying no to being in this study.

Please ask questions about anything that you don't understand or want to know more about. Before making a decision, you might also want to talk about it with others.

What is oncho and what is being done to help people with oncho now?

Oncho is caused by a worm that is passed from one person to another through the bites of the small black flies that you see at the riverside and in your community in the mornings and early evenings. Two types of these worms live in the human body. The young worms in the skin and eyes cause the disease, for example itching or a rash. The adult worms live for up to 14 years in swellings under the skin called nodules and produce millions of the young worms.

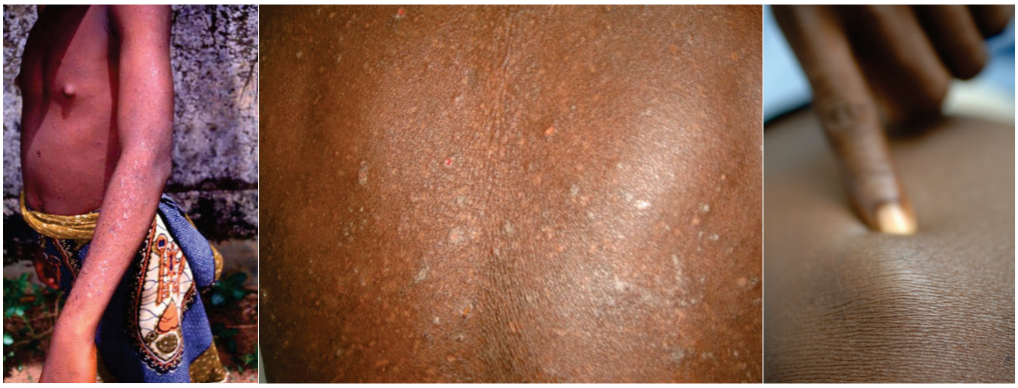

You and many of the adults and other children in your community may have oncho. Therefore, a medicine called ivermectin is now given to all people at least 5 years of age who are not pregnant or sick.

Ivermectin kills most of the young worms so that people who have oncho and take ivermectin have less or even none of the disease. However, the adult worms quickly start to make new worms and so ivermectin is given to you every six months.

What is the new medicine, moxidectin, and what does it do against the oncho worms?

You may have already heard about moxidectin because about 10 years ago, 219 people from Wii, Azua, Jagri Akua and Bitaaba who had oncho and were at least 12 years of age took part in a study to test moxidectin. In that study, some people were given moxidectin and some people were given ivermectin. If you know anybody who took part in that study, they can tell you about their experience in the study.

In the study, we found that moxidectin kills more young oncho worms in the skin than ivermectin and that moxidectin stops the adult worms from making new, young worms for a longer time than ivermectin. Here we are showing you pictures of the number of oncho worms in people before and after they took moxidectin or ivermectin.

Number of Young Worms in the Skin Before and After Taking Moxidectin or Ivermectin

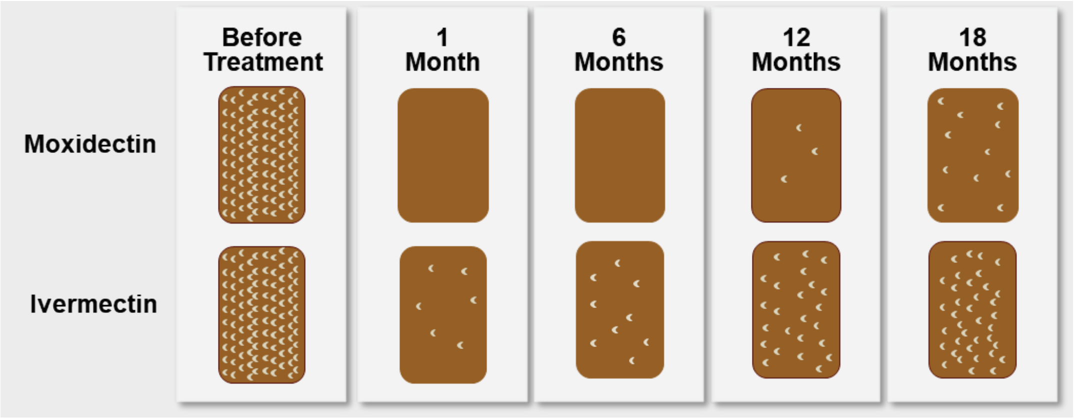

What do we know about unwanted effects of moxidectin?

In that study we also found that people who took moxidectin and people who took ivermectin had similar unwanted effects, but some unwanted effects occurred in more people who took moxidectin than in people who took ivermectin. Many of these unwanted effects are caused by the body getting rid of the young oncho worms after they have been killed by moxidectin or ivermectin. On the next page we show you how many out of 100 people had unwanted effects after taking moxidectin or ivermectin. Please ask me if you don’t understand what these unwanted effects mean.

Number of People with Unwanted Effects in 100 People Who Took Moxidectin or ivermectin

Unwanted Effect	Moxidectin	Ivermectin
Itching	65 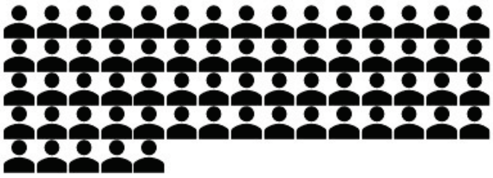	54 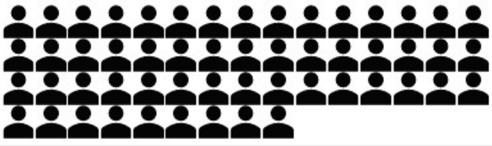
Muscle pain	64 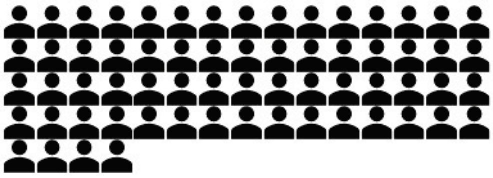	52 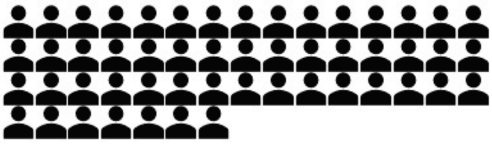
Headache	58 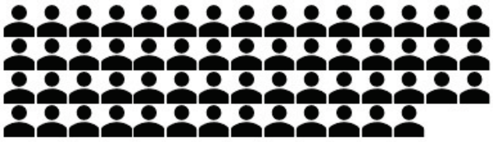	54 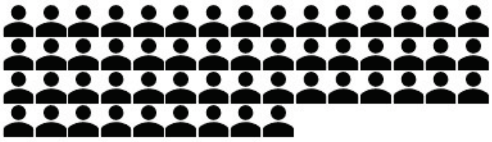
Fast heartbeat	39 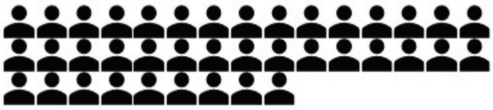	30 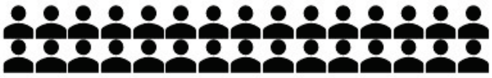
Rashes	37 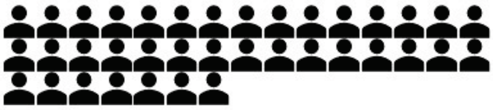	21 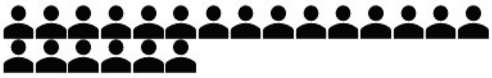
Stomach pain	31 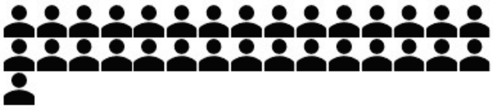	35 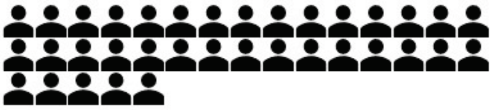
Feeling faint or dizzy when standing up	30 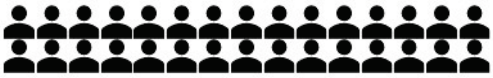	25 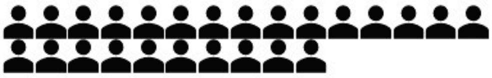
Fever /chills	27 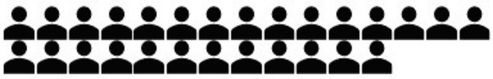	18 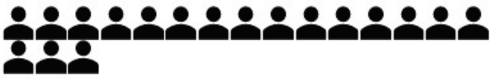

Number of People with Unwanted Effects in 100 People Who Took Moxidectin or ivermectin

Unwanted Effect	Moxidectin	Ivermectin
Common cold	23	21
Cough	17	18
Upset belly (Stomach flu)	15	17
Lymph node pain	13	6
Dizziness	12	9
Swollen arm or leg	11	6

Why are we doing a study of moxidectin in 4 to 17 year olds and asking you to take part?

We have already given moxidectin to 1349 people with oncho. These included 53 adolescents aged 12 to 17 years with oncho who took 4 tablets of moxidectin (8 milligrams), the same number of tablets adults with oncho took. Thirty-one (31) of them lived in Wii, Azua, Jagri Akua or Bitaaba. From this study, we know that 4 tablets is the right number of tablets for people at least 12 years of age.

Because moxidectin works better against the oncho worms than ivermectin, it would be good if moxidectin could also be given to children aged 4 to 11 years. Therefore, we want to find the right number of moxidectin tablets for children aged 4 to 11 years.

How will we find out what number of moxidectin tablets is right for children aged 4 to 11 years?

When somebody takes moxidectin, it gets into their blood and from the blood, moxidectin can reach the young oncho worms in the body to kill them.

The right number of tablets for children aged 4 to 11 years is the number that leads to the same amount of moxidectin in their blood as in the blood of adults and adolescents 12 to 17 years of age who take 4 moxidectin tablets and does not cause severe unwanted effects.

To find out the right number of tablets for children aged 4 to 11 years, we need to first give moxidectin tablets to adolescents aged 12 to 17 years, take a bit of blood and measure the amount of moxidectin in their blood and find out what unwanted effects they have.

We think that we will have to give moxidectin to at least nine adolescents aged 12 to 17 years but not more than 18.

What will happen in the study, where will it happen and how long will it take?

First, we will do Screening. Screening means that we fill find out whether you have the oncho worm but are otherwise healthy and can take part in the study.

The first step of Screening will happen in your community:

- We will ask if you have a sister or brother who has already taken part in the study, because only one child from your family can take part.
- If you are a young woman, we will ask whether you are breast-feeding a child because then you cannot take part in the study.
- We will ask questions about health problems you have had in the past, medicines you have taken, look closely at your arms to see if it will be easy to take blood.

This will take 1 to 2 days.

If we find you cannot take part in the study, we will tell you and your parents or guardians why.

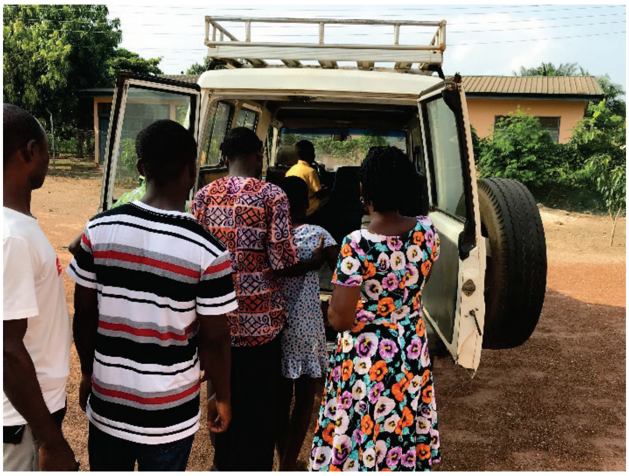

If we find that you can go on to **the second step of Screening**, we will drive you and one of your parents or guardians by car to our study center in Hohoe.

In Hohoe we have a special house for people taking part in our study and you will sleep there together with your parent or guardian and other children and their parents or guardians who take part in our study. We will give you free meals while you are in Hohoe.

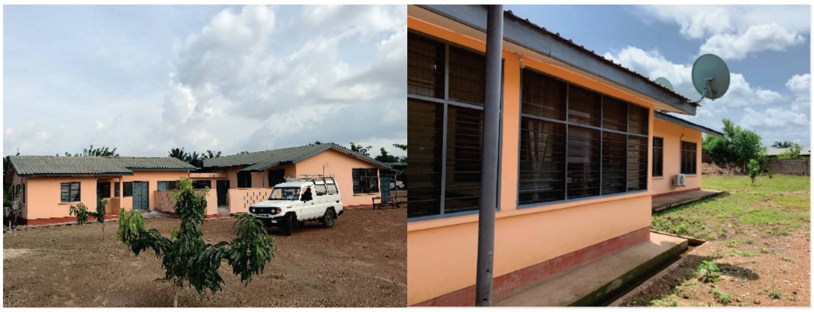

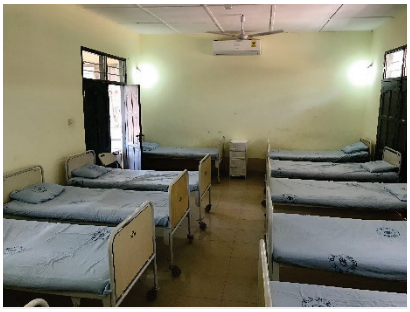

In Hohoe:

- We may ask you and your parent or guardian to tell us more about health problems you have had in the past and any medicines you have taken lately.
- We will measure your height and weight.
- We will examine how fast you breathe, how hard and fast your heart works and your body temperature (we call these measurements “vital signs”).
- We will examine how well your heart works with a special machine called an electrocardiograph (an “ECG”). Here I am showing you the things we will put on your body for the ECG, like you see in this picture. This won’t hurt.
- We will examine your body for signs of illness or pain. Some of this you can see in the pictures here.

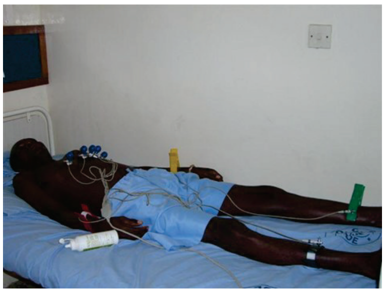

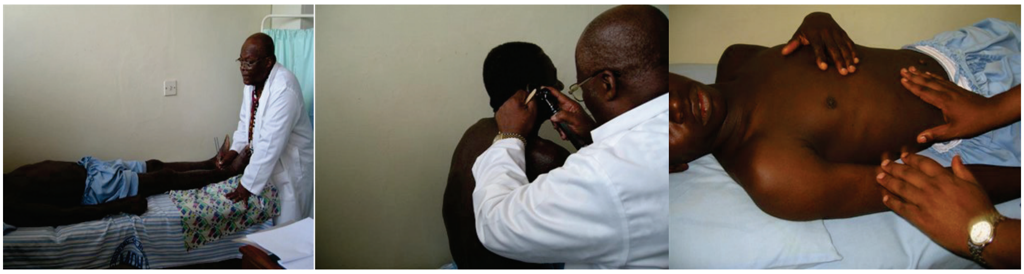

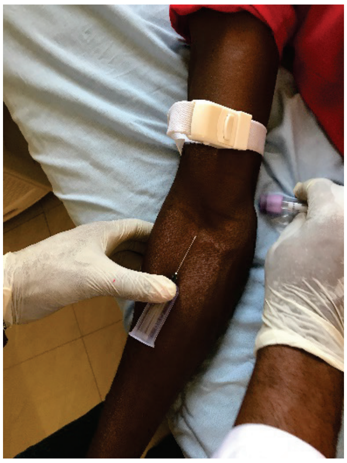

- Doctors can learn a lot about whether you are sick by testing your blood. We will take a bit of your blood (12.5 milliliters or a little bit more than 2 teaspoons) like you can see in this photo. Before we take the blood, we will put a cream on your arm so you won't feel much pain.

  We will test the blood to find out how well some of the organs in your body work and for signs of HIV (AIDS infection) or the germs that cause liver disease (hepatitis B and C).

  If you have lived outside Ghana in an area where another worm called Loa loa occurs, we will test your blood to check whether you have the Loa loa worm. We will do this because people with lots of Loa loa worms can have more unwanted effects when taking moxidectin and therefore they should not take part in our study.

- If you are a girl who has started her monthly bleeding, we will also test your blood to see whether you are pregnant. If you are pregnant, you cannot take part in the study.

All of this will take 1 to 2 days.

We will explain everything we learn about your health. We will let you and your parent or guardian know whether you can take part in the study.

What will happen after Screening if you cannot take part in the study?

We will explain to you and your parent or guardian why you cannot take part in the study. If you need to be taken care of by a nurse or doctor, we will arrange for you to visit a clinic or hospital.

On the day after Screening, we will drive you and your parent or guardian back to your community.

What will happen after Screening if you can take part in the study?

We will ask you to stay in Hohoe for 8 more days.

Girls can take part in this study only if they are not pregnant and if they avoid becoming pregnant until around 6 months after they have swallowed moxidectin. We will tell you more about this a bit later.

You will not be able to take ivermectin while you are on the study (for approximately 6 months).

On the day after Screening, we will:

- Repeat some of the examinations we did during Screening before we give you moxidectin.

- Ask you to swallow 4 moxidectin tablets 2 hours before you have breakfast.
- Repeat again some of the examinations we did during Screening.
- Take a bit of your blood (1.5 milliliters or 1/3 of a teaspoon) 4 times to measure the amount of moxidectin. We will use a special needle called a 'cannula' so that you only need to have one needle prick and we will use the special cream, so you won't feel much pain.
- Ask you several times to tell us if you are feeling unwell or have any pain. If you have pain or are feeling unwell, we will examine you to find out what we can do to make you feel better. If necessary, we will give you medicine.
- We will also ask you and your parent or guardian to tell us immediately if you are not feeling well. Even during the night, there will always be a nurse to talk to.

During the next 7 days, we will:

- Repeat some of the examinations we did during Screening.
- Take a bit of your blood (1.5 milliliters or 1/3 of a teaspoon) on 2 days to measure the amount of moxidectin.
- Take a bit more of your blood (3.5 milliliters total, 2/3 teaspoon) on the last day to measure the amount of moxidectin and test how well some of the organs in your body work.

If you are feeling well, we will drive you and your parent or guardian back to your community on the 7th day after you have taken the moxidectin tablets. If not, we will ask you to stay longer until you feel well.

2 weeks, 1 month, 3 months and around 6 months after you have swallowed the moxidectin tablets, we will:

- Drive you and your parent or guardian to Hohoe again.
- Each time we will ask you to tell us about any health problems and any medicines taken since we last saw you.
- Each time we will repeat some of the examinations we did when you were last in Hohoe.
- We will take a bit of your blood (1.5 milliliters or 1/3 of a teaspoon) on 2 visits to measure the amount of moxidectin in it.
- We will take a bit more of your blood (3.5 milliliters or 2/3 teaspoon) on 1 visit to measure the amount of moxidectin in it and to find out how some of the organs in your body work.
- Each time you and your parent or guardian will spend one night with us and we will give you free meals.

What happens when you are in Hohoe during school time?

There will be a teacher in our study center in Hohoe. The teacher will help you keep going with some of your schoolwork, to help make up for the school time you miss.

What should you do if you are not feeling well when you are back in your community?

You should let your parents or guardians know and contact us, either through the community coordinator or directly or you should go to the closest health clinic. The 'community coordinator' is the person chosen by the adults in your community to contact us whenever you want. We have written down the name for you.

We will make sure that you are examined and receive medicine if you need it. If needed, we will drive you and your parent or guardian to the study center in Hohoe or a health clinic or hospital.

Are there any potential advantages of taking part in this study?

You may not have any advantages.

- You will have examinations of your health. These examinations are often used by doctors and nurses to find out whether people are sick.
- If you have young oncho worms in your skin or eyes, moxidectin will cause most or all of them to die and prevent new young worms in your skin for up to about 1 year.
- If you have health problems because of the young oncho worms in your skin or eyes, these will become better or even go away for up to about 1 year.

Are there any possible risks of taking part in this study?

Unwanted effects of taking moxidectin

Almost all medicines cause unwanted effects.

We have already told you about the unwanted effects we found in people with the oncho worm who took moxidectin or ivermectin. In most cases, these unwanted effects were mild, went away on their own and lasted less than a week. Please let me know if you would like us to look at the pictures again.

You may have none, some or all of these unwanted effects. They may be mild, moderate or severe. They may occur soon after you have taken moxidectin or many hours or days later.

Also, because moxidectin is a new medicine, there may be unwanted effects that we don't know about yet and we don't know whether they would be mild, moderate or severe or how long they may last. If we learn about new unwanted effects that may make you change your mind about taking part in this study, we will tell you and your parents or guardians immediately.

Unwanted effects of taking blood

Having blood taken may cause some pain, bruising, and a little bit of bleeding. Sometimes, it can also cause minor infection (where germs can get into the needle prick) or fainting. This has never happened in our studies because we get you to lie down and clean the skin to remove any germs before we take blood. If you have any unwanted effects, they can easily be treated.

Why should girls who are pregnant or breast-feeding not take moxidectin and why should girls ensure that they don't become pregnant during the study?

As you may know, when ivermectin is distributed in your community, women who are pregnant should not take ivermectin until at least a week after their baby is born.

We don't know the effects of moxidectin on an unborn child or newborn baby that is breastfed by a mother who has taken moxidectin. Because of this, girls who are pregnant or breast-feeding cannot take part in this study and girls should not become pregnant during the study.

If you already have monthly bleeding, we will test whether you are pregnant.

How can girls avoid becoming pregnant?

You must agree to avoid becoming pregnant for around 6 months (until your last visit to Hohoe).

A family planning nurse will discuss highly effective methods to avoid becoming pregnant with you and your parent or guardian. We call these methods "methods of contraception". One method of contraception is not having sex. If you choose another method of contraception, we will provide it to you.

What happens if a girl who takes part in the study becomes pregnant?

If you become pregnant between taking moxidectin and around 6 months later, you should let us know immediately. We will advise you to attend all ante-natal care visits the Ghana Health Service offers. A doctor specializing in treating babies and children will examine the baby after birth and at least once a year until 2 years of age.

What happens if a boy who takes part in the study makes someone pregnant?

If you make someone pregnant between taking moxidectin and around 6 months later, you should also inform us immediately. We will ask you to let us visit your partner. We will advise her to attend all ante-natal care visits the Ghana Health Service offers and ask her to agree that a doctor specializing in treating babies and children will examine the baby after birth and at least once a year until 2 years of age.

What happens to the blood samples?

We will send the blood for measuring the amount of moxidectin to people in another country (the United States of America) to make the measurements. We will do the other tests in our country.

We will not write your name on the blood samples, only a number. We call this number your ‘participant number’. Only we know which number belongs to which adolescent or child who takes part in our study.

We will only use your blood for measuring the amount of moxidectin and for the tests we have told you about. Once these measurements and tests are done, any blood left over will be destroyed.

Who will know what we found out about your health during the study?

We will keep the information we collect about your health in what we call “records”. We will keep these records private (confidential). This means that we will not share your name with anybody who does not belong to our study team without your permission, except when we must because it is the law.

We will keep all the records in a locked, secure area for at least 25 years and possibly longer. When we don’t need them anymore, they will be destroyed.

The people who can see the records, including your name, are:

- The doctors, nurses and laboratory staff working in our study team.
- People coming to our study center in Hohoe to make sure that we do things right and follow the law. These are:
    - People from the Ghana Food and Drugs Authority and the United States Food and Drug Administration.
    - People from the ‘Sponsor’. The Sponsor is Medicines Development for Global Health, who make moxidectin tablets. They are giving us the money and the moxidectin tablets for the study and helping us write reports about what we learn.
    - People from the “Ethics Committees”. These are people that have been asked by the University of Health and Allied Sciences in Hohoe, the Ghana Health Service or the World Health Organization to make sure we do this study in the right way.

The people who can see the study records but NOT your name or the names of your parents or guardians are:

- People from the Sponsor and working with us and the Sponsor. The Sponsor will keep the study records without your name for at least 25 years.
- People from the Ghana Food and Drugs Authority, the United States Food and Drug Administration, or the authorities in other countries or at the World Health Organization

who look at study results to decide whether moxidectin can be used in Ghana and other countries.

- Other people who want to learn about what happens when children 4 to 17 years of age take moxidectin.

If you change your mind about taking part in the study and don't want to take part anymore, we will stop collecting information. We and the Sponsor will still use the information that we collected before you told us you want to stop being in the study. However, if you have an unwanted effect that has not stopped, we will ask you to let us visit you to find out whether you need any treatment for this effect. Also, if you are pregnant, we will ask you if the doctor working with us who specializes in treating babies and children can come and examine the baby after birth and at least yearly for 2 years. If you are male and your partner becomes pregnant, we will ask you if we can contact your partner to ask her if we can examine the baby. You do not have to agree to this.

Will you or your parents have to pay anything to take part in the study?

You or your parents or guardians will not pay anything.

- All examinations and medical care that are part of the study will not cost anything.
- When you and your parent or guardian stay overnight in Hohoe, you will not pay anything. We will give you free food and transportation by car to and from your home.

Will your parent/guardian be compensated for the time they spend with you at the study center?

The parent or guardian who goes with you to Hohoe will be compensated, that means get money to make up for loss of earnings. The amount will be based on the number of nights spent in Hohoe. The amount for each night's stay will be roughly the "by day" earnings of adults in your community, currently forty (40.00) Ghana Cedis per day.

What happens if you have a permanent injury or health problem because you take part in this study?

We do not think that anybody will have an injury from the study that will make them permanently sick or die. However, if this happens, the Sponsor's insurance will pay you or your parents or guardians compensation.

What happens after the study?

After the study, you should take ivermectin when it is distributed in your community.

We will tell you, your parents or guardians and your community about what we learnt. We will also tell you what we and the Sponsor plan to do so that the Ghana Health Service and the health services in other countries where people have oncho can decide whether they want to give out moxidectin in the way they are now giving out ivermectin.

Who can you talk to if you want more information before you agree to take part in the study or during the study?

If you want to talk to someone who is not in our study team about any worries about the study, your rights, an injury you may have suffered in the study, or any other questions, concerns or complaints about the study in the future, please contact:

- The **Administrator of the Research Ethics Committee, Institute of Health Research, University of Health and Allied Sciences** by email at rec@uhas.edu.gh or by telephone on **+233 362 196 193**.
- The **Secretary to the Research Ethics Committee, Institute of Health Research, University of Health and Allied Sciences, Mr. Fidelis Anumu**, by telephone on + **233 244 061 270**. If you or your child have any questions, concerns or complaints about the study in the future, you may also contact the Project Administrator later.
- The **Administrative Secretary of the Ghana Health Service Ethics Review Committee, Ms Nana Abena Apatu,** by telephone on **+233 503 539 896** or by writing to The Administrative Secretary, Ghana Health Service Ethics Review Committee, Research and Development Division, Ghana Health Service, P. O. Box M190 Accra Ghana.

If you have any questions about the study or need medical help during the study, please contact the following members of the study team or your community coordinator:

1. **Dr. Nicholas O. Opoku (On-site Principal Investigator)**
   University of Health and Allied Sciences
   School of Public Health Research Centre
   Municipal Hospital Hohoe,
   Telephone**: 03627 22042** or **0244 776668** (mobile)
2. **Dr. Felix Doe (Co-investigator)**
   Hohoe Municipal Health Directorate
   Telephone: **0208 437550** or **0245 118342** (mobile)
3. Your **Community Coordinator**

   Name: ______________________________

   Telephone: ______________________________

What if you or your parents/guardians do not want you to take part in the study or if you or your parents/guardians change your mind?

It is your and your parents' or guardians' decision to take part in this study.

You or your parents or guardians can tell us that you will stop taking part in the study at any time. You do not have to tell us why you want to stop.

Not taking part in the study or changing your mind will not change the health care you and your family will receive from the Ghana Health Service.

When you tell us that you will stop taking part in the study, we will ask you to let us do final examinations so that we know about your health and can tell you what you should look out for after leaving the study.

We may decide that you cannot continue in the study. We will do this if you are not ready to come to Hohoe or if you do not want us to do the examinations or take blood, or if we find that it is better for your health. If this happens, we will explain the reasons to you and your parents or guardians.

What do you and your parents/guardians need to do if you want to take part in the study?

You should discuss this with your parents and other people.

You can only take part if both you and your parents or guardians agree that you can take part.

After you have decided, please come and talk to us together with your parents or guardians and the person your community had chosen to be present when we provide you the information about the study. We will call this person "the witness".

We will ask you and your parents or guardians whether you have any questions. We will also ask you and your parents or guardians some questions to make sure you all know what will happen during the study. Then we will ask you and your parents or guardians to confirm that you want to take part in the study by putting your signature or thumbprint on a form. The witness will also sign the form.

Help for your discussion with your parents/guardians, friends, family and others you want to talk this over with

To help you in your discussions of the study with others, here is an overview of what will happen and where it will happen.

You and your parents or guardians discuss whether you can take part in the study and you all agree. → If you do not agree you don't need to do anything.

↓

If you agree, you let us know. We will check whether we need to tell you more about the study. We will ask you and your parents or guardians to sign or thumbprint a form.

↓

Step 1 Screening in your community to ask questions about your health (1-2 days). → If you cannot take part in Step 2 Screening, we will tell you why and tell you if you should see a nurse or doctor.

↓

If you can take part in **Step 2 Screening**, you and one of your parents/guardians will go to Hohoe.

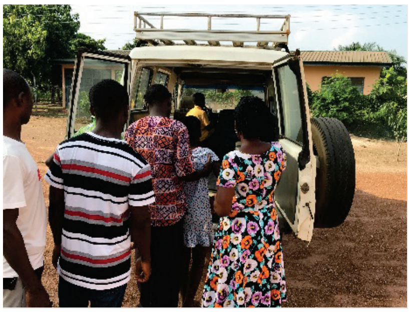

You will stay in our special house with other children who will take part in the study and their parents or guardians.

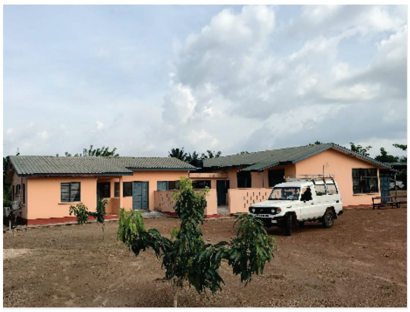

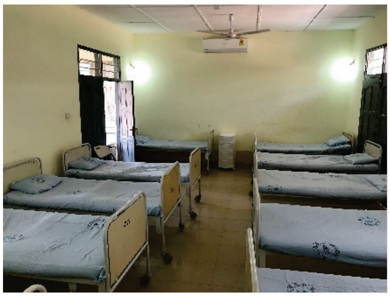

We will ask more questions, do examinations and take bits of blood to learn more about your health and find out whether you can take part in the study (1-2 days).

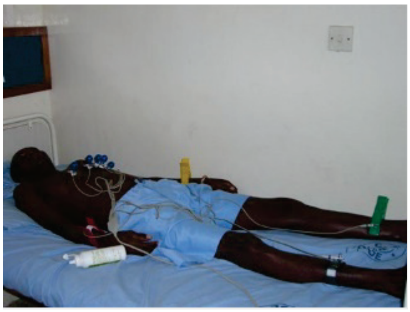

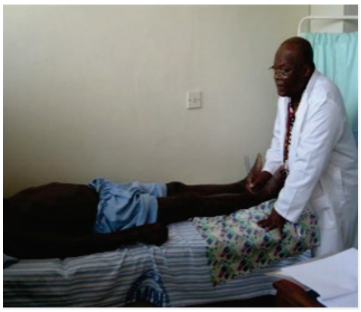

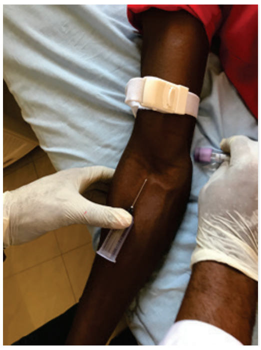

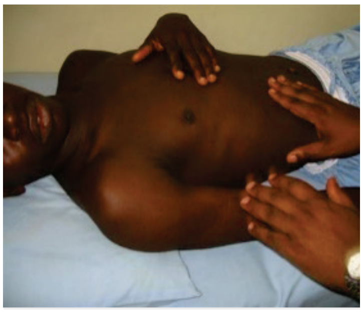

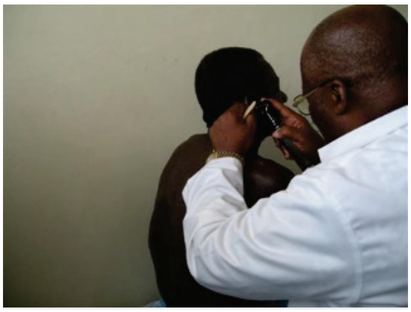

If you can take part in the study and you still agree, you need to stay in Hohoe for 8 more days.

If you cannot take part in the study, we will tell you why and take you home. If you have a health problem, we will arrange a visit to a clinic or hospital near your community.

The next day, we will ask you to swallow the moxidectin tablets.

On that day and the next 7 days we will take more bits of your blood and repeat some of the examinations we did during Screening.

Then we will take you back to your community, unless you are not well, in which case you will stay until you are better.

2 weeks, 1 month, 3 months and around 6 months after you swallowed the moxidectin tablets, we will bring you and one parent or guardian back to Hohoe.

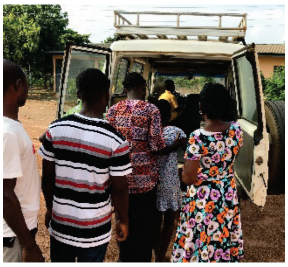
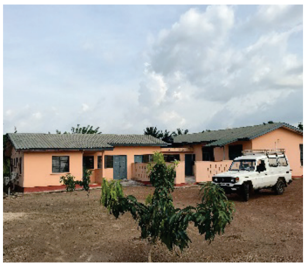
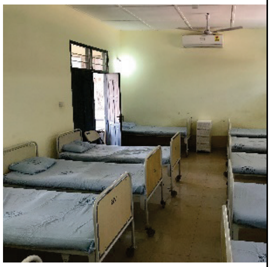

Each time we will repeat some of the examinations we did when you were in Hohoe last time and on 3 visits, we will take a bit of your blood.

**Assessment of Informed Assent – Questions for Adolescents 12 to 17 Years**	**Child Response**
Do you understand why you are being asked to participate in this study?	☐ Yes ☐ No
Have you been able to ask questions and discuss the study?	☐ Yes ☐ No
Questions:	
Do you understand that you can only take part if both you and your parents/guardians agree?	☐ Yes ☐ No
Do you understand that during Screening we might find that you cannot take part in the study?	☐ Yes ☐ No
Will we take blood during this study?	☐ Yes ☐ No
How long will you be in the study?	

**Assessment of Informed Assent – Questions for Adolescents 12 to 17 Years**	**Child Response**
Do you understand you can tell us at any time that you want to stop being in the study, without having to tell us why?	☐ Yes ☐ No
Is there any charge for being in the study?	☐ Yes ☐ No
Do you understand that this is a study and that we do not know all unwanted effects of moxidectin?	☐ Yes ☐ No
Do you know who to call if you have questions?	☐ Yes ☐ No
Do you know that we will share what we learn about your health with many different people but only those coming to Hohoe to check we do things right can learn your name?	☐ Yes ☐ No
Are you willing to come to Hohoe five times and have the examinations and blood taken that we told you about?	☐ Yes ☐ No
For girls who already have monthly bleeding: Do you understand that you must avoid becoming pregnant for around 6 months and that we will give you what you need if you chose a method other than not having sex?	☐ Yes ☐ No

**Assessment of Informed Consent – Questions for Parents/ Guardians of Adolescents 12 to 17 Years**	**Parent/Guardian Response**
Do you understand why your child/ward is being asked to participate in this study?	☐ Yes ☐ No
Have you been able to ask questions and discuss the study?	☐ Yes ☐ No
Questions:	
Do you understand that during Screening we might find that your child/ward cannot take part in the study?	☐ Yes ☐ No
Do you understand that it is your decision to allow your child/ward to take part in this study and that your child/ward must also agree (that means your child is a "volunteer")?	☐ Yes ☐ No

**Assessment of Informed Consent – Questions for Parents/ Guardians of Adolescents 12 to 17 Years**	**Parent/Guardian Response**
Are you willing to allow your child/ward to have all the examinations?	☐ Yes ☐ No
Will we take blood from your child/ward during this study?	☐ Yes ☐ No
How long will your child/ward be in this study?	
Do you understand your child/ward can stop taking part in the study at any time, without having to tell us why?	☐ Yes ☐ No
Is there any charge for being in the study?	☐ Yes ☐ No
Do you understand that this is a study and that we do not know all unwanted effects of moxidectin?	☐ Yes ☐ No
Do you know who to call if you or your child/ward has questions?	☐ Yes ☐ No
Do you know that we will share what we learn about the health of your child/ward with many different people but only those coming to Hohoe to check we do things right can learn the child's name?	☐ Yes ☐ No
Are you willing to come to Hohoe five times and so that your child/ward can have the examinations and blood taken that we told you about?	☐ Yes ☐ No
For parents/guardians of girls old enough to have a baby: Do you understand that your daughter/ward has to avoid becoming pregnant and that we will give her what she needs if you and she choose a method other than not having sex?	☐ Yes ☐ No

507

4. Information document for parents and guardians of 4- to 11-year-old children

General Information About the Study

I am Dr.., a doctor working at the University of Health and Allied Sciences in Hohoe. We are testing a new medicine called moxidectin in children 4 to 17 years of age. Moxidectin is a new treatment for onchocerciasis (River blindness), which you call "*oncho*".

Testing new medicines is called doing "research" or "a study" and means we want to learn something about the new medicine.

Here, we explain to parents of children aged 4 to 11 years what we already know about moxidectin, what will happen during the study and the risks and advantages of taking part in this study. This will help you decide whether you will allow your child or ward to take part in the study or not.

If you don't want your child or ward to take part, please don't be afraid to tell us. Your care or the care of your child or ward at your local health care centers will not be affected by saying no.

Please ask questions about anything that you don't understand or want to know more about. Before making a decision, you might also want to talk about it with others.

What is oncho and what is being done to help people with oncho now?

Oncho is caused by a worm that is passed from one person to another through the bites of the small black flies that you see at the riverside and in your community in the mornings and early evenings. Two types of these worms live in the human body. The young worms in the skin and eyes cause the disease, for example itching or a rash. The adult worms live for up to 14 years in swellings under the skin called nodules and produce millions of the young worms.

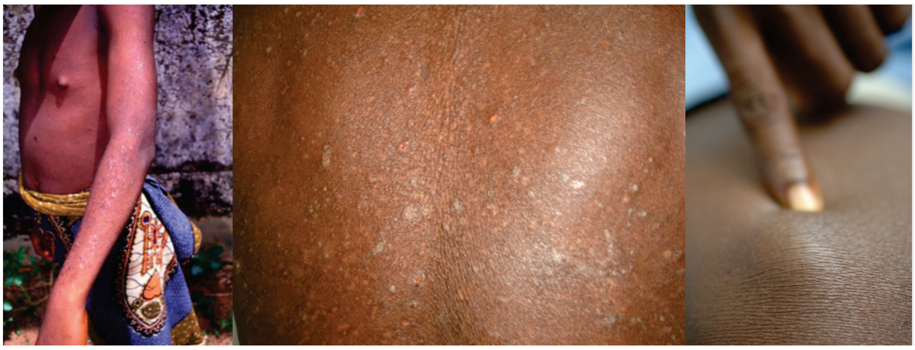

Many adults and children in your community may have oncho. Therefore, a medicine called ivermectin is now given to all people at least 5 years of age who are not pregnant or sick.

Ivermectin kills most of the young worms so that people who have oncho and take ivermectin have less or even none of the disease. However, the adult worms quickly start to make new worms and so ivermectin is given to you every six months.

What is the new medicine, moxidectin, and what does it do against the oncho worms?

You may have already heard about moxidectin because about 10 years ago, 219 people from Wii, Azua, Jagri Akua and Bitaaba who had oncho and were at least 12 years of age took part in a study to test moxidectin. In that study, some people were given moxidectin and some people were given ivermectin. If you know anybody who took part in that study, they can tell you about their experience in the study.

In the study, we found that moxidectin kills more young oncho worms in the skin than ivermectin and that moxidectin stops the adult worms from making new young worms for a longer time than ivermectin. We are showing you here pictures of the number of oncho worms in people before and after they took ivermectin or moxidectin.

Number of Young Worms in the Skin Before and After Taking Moxidectin or Ivermectin

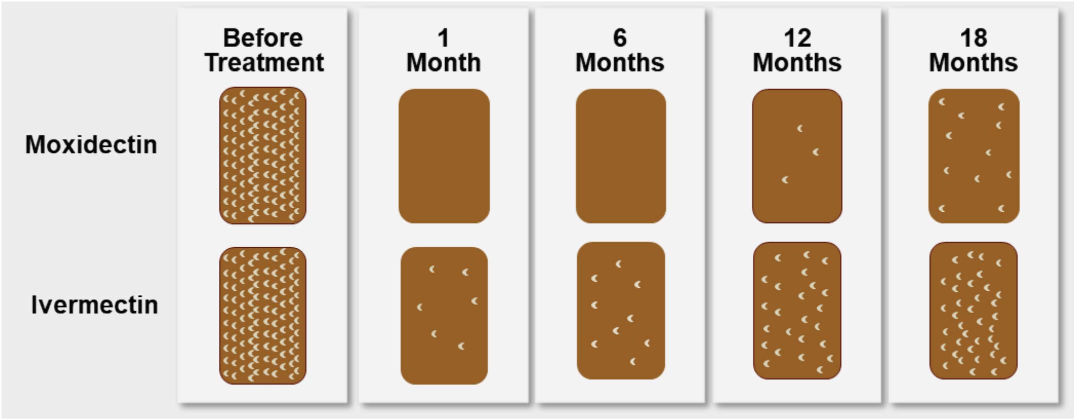

What do we know about the unwanted effects of moxidectin?

In that study we also found that people who took moxidectin and people who took ivermectin had similar unwanted effects but some unwanted effects occurred in more people who took moxidectin than in people who took ivermectin. Many of these unwanted effects are caused by the body getting rid of the young oncho worms after they have been killed by moxidectin or ivermectin. On the next page we show you how many out of 100 people had unwanted effects after taking moxidectin or ivermectin. Please ask me if you don't understand what these unwanted effects mean.

Supplementary information to:

Opoku et al. Identification of a moxidectin dose for 4-to 11-year-old children to support registration and potential use for onchocerciasis elimination: Results of an open-label pharmacokinetic and safety study in an onchocerciasis endemic area in Ghana

Number of People with Unwanted Effects in 100 People Who Took Moxidectin or Ivermectin

Unwanted Effect	Moxidectin	Ivermectin
Itching	65 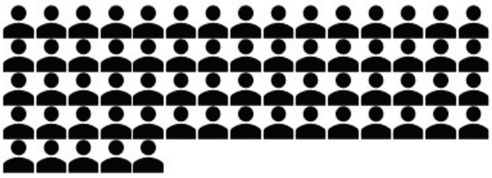	54 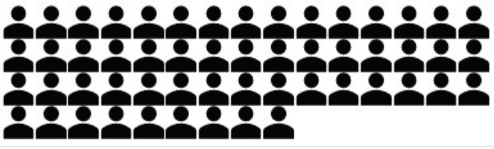
Muscle pain	64 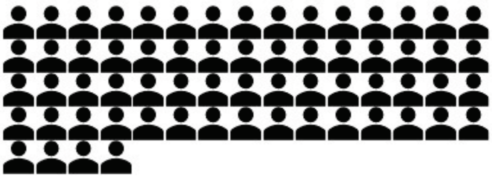	52 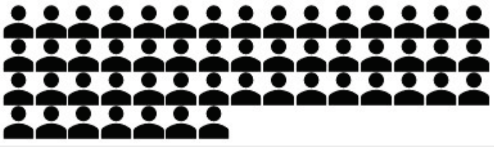
Headache	58 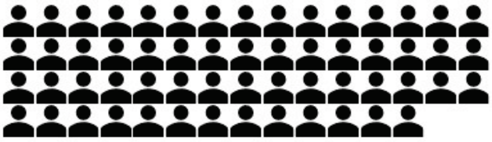	54 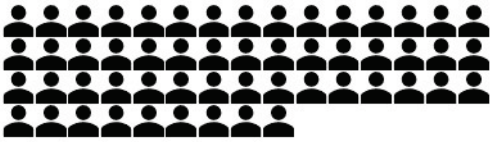
Fast heartbeat	39 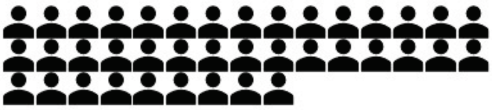	30 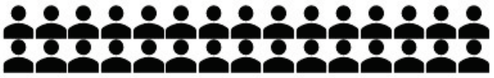
Rashes	37 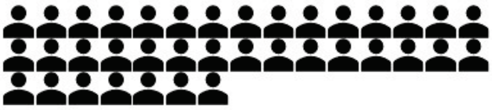	21 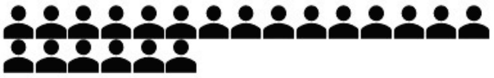
Feeling faint or dizzy when standing up	30 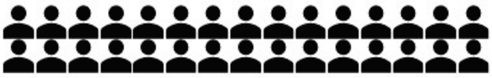	25 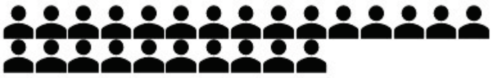
Stomach pain	31 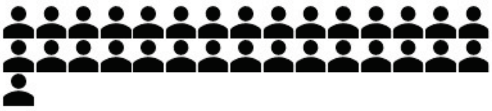	35 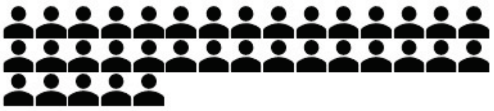
Fever /chills	27 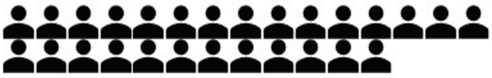	18 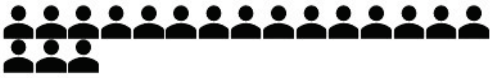

Number of People with Unwanted Effects in 100 People Who Took Moxidectin or Ivermectin

Unwanted Effect	Moxidectin	Ivermectin
Common cold	23	21
Cough	17	18
Upset belly (Stomach flu)	15	17
Lymph node pain	13	6
Dizziness	12	9
Swollen arm or leg	11	6

Why are we doing a study of moxidectin in 4 to 17 year olds and asking your permission for your child/ward to take part?

We have already given moxidectin to 1349 people with oncho. These included 53 adolescents aged 12 to 17 years with oncho who took 4 tablets of moxidectin (8 milligrams), the same number of tablets adults with oncho took. Thirty-one (31) of them lived in Wii, Azua, Jagri Akua or Bitaaba. From this study, we know that 4 tablets is the right number of tablets for people at least 12 years of age.

Because moxidectin works better against the oncho worms than ivermectin, it would be good if moxidectin could also be given to children aged 4 to 11 years. Therefore, we want to find the right number of moxidectin tablets for children aged 4 to 11 years.

How will we find out what number of moxidectin tablets is right for children aged 4 to 11 years?

When somebody takes moxidectin, it gets into their blood and from the blood, moxidectin can reach the young oncho worms in the body to kill them.

The right number of tablets for children aged 4 to 11 years is the number that leads to the same amount of moxidectin in their blood as in the blood of adults and adolescents aged 12 to

17 years who take 4 moxidectin tablets. Of course, the children should also not have severe unwanted effects.

To find out the right number of tablets for children aged 4 to11 years, we need to give them moxidectin tablets, take a bit of blood and then measure the amount of moxidectin in their blood and find out what unwanted effects they have.

We will start by giving 4 moxidectin tablets to 9 adolescents aged 12-17 years and 9 children aged 8 and 11 years. We will measure the amount of moxidectin in their blood and examine them for unwanted effects. If the children aged 8 to 11 years have more moxidectin in their blood or more unwanted effects than we saw in adults or adolescents aged 12-17 years who took moxidectin, we will give a smaller number of moxidectin tablets to another group of 9 children 8 to 11 years of age and measure the amount of moxidectin in their blood and examine them for unwanted effects.

When we have learnt the right number of moxidectin tablets for children aged 8 to 11 years, we will ask 9 children aged 4 to 7 years children to take part in the study.

We will give them moxidectin tablets and measure the amount of moxidectin in their blood and examine them for unwanted effects. If they have less moxidectin in their blood than we saw in adults or adolescents aged 12 to 17 year or children 8 to 11 years of age, we will give more moxidectin tablets to a second group of 9 children aged 4 to 7 years. If the first group have too much moxidectin or too many unwanted effects, we will give fewer moxidectin tablets to a second group of children aged 4 to 7 years.

We think that we will have to give moxidectin to at least 9 children aged 8 to 11 and at least 9 children aged 4 to 7 years, but maybe up to 52 children aged 4 to 11 years.

What will happen in the study, where will it happen and how long will it take?

First, we will do Screening. Screening means that we fill find out whether your child or ward has the oncho worm but is otherwise healthy and can take part in the study.

The first step of Screening will happen in your community:

- We will ask if a sister or brother has already taken part in the study, because only one child or ward from your family can take part.
- We will ask questions about health problems in the past, medicines taken, look closely at the child’s arm to see if it will be easy to take blood, and show an item that is the same size as the moxidectin tablets and ask the child and you if they could swallow a tablet of that size.

This will take 1 to 2 days.

If we find your child can not take part in the study, we will tell you and the child why.

If we find that your child can go on to **the second step of Screening**, we will drive the child and one of you by car to our study center in Hohoe.

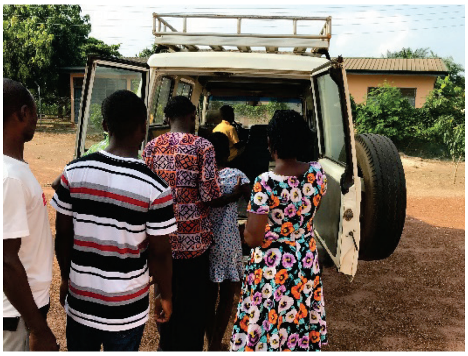

In Hohoe we have a special house for people taking part in our study and you will sleep there together with the child and other children and their parents or guardians who take part in our study. We will give you free meals while you are in Hohoe.

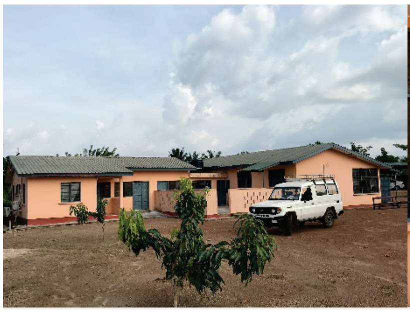

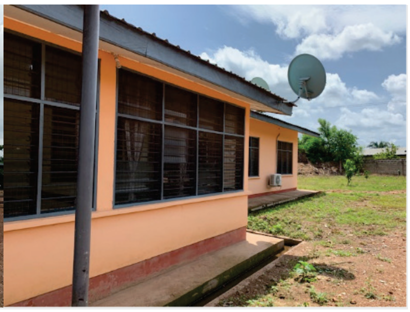

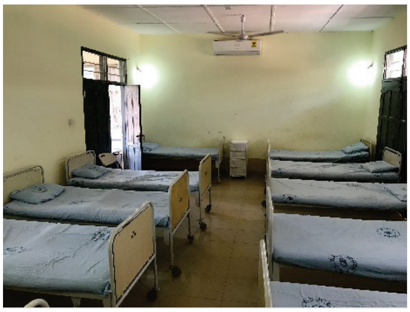

In Hohoe:

- We may ask you and the child to tell us more about the child's health problems in the past and any medicines they have taken lately.
- We will measure the child's height and weight.
- We will examine how fast the child breathes, how hard and fast the heart works and the body temperature (we call these measurements "vital signs").
- We will examine how well the heart works with a special machine called an electrocardiograph (an "ECG"). Here I am showing you the things we will put on your body for the ECG, like you see in this picture. This won't hurt.
- We will examine the child's body for signs of illness or pain. Some of this you can see in the pictures here.

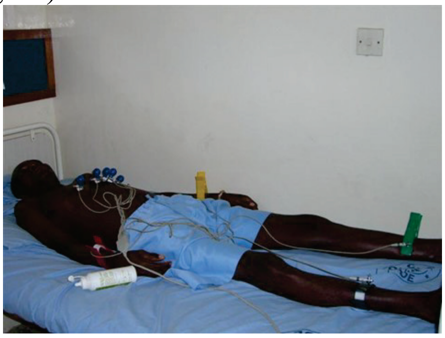

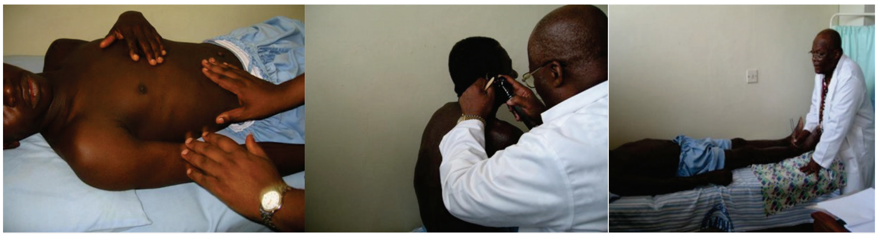

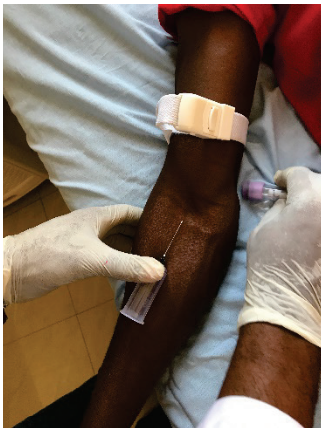

- Doctors can learn a lot about whether somebody is sick by testing the blood. We will take a bit of blood (no more than 8.5 milliliters or about 2 teaspoons) like you can see in this photo. Before we take the blood, we will put a cream on the child's arm so they won't feel much pain.

  We will test the blood to find out how well some of the organs in the child's body work and for signs of HIV (AIDS infection) or the germs that cause liver disease (hepatitis B and C).

  If the child has lived outside Ghana in an area where another worm called Loa loa occurs, we will test the blood to check whether the child has the Loa loa worm. We will do this because people with lots of Loa loa worms can have more unwanted effects when taking moxidectin and therefore they should not take part in our study.

All of this will take 1 to 2 days.

We will explain everything we learn about the child's health. We will let you and the child know whether the child can take part in the study.

What will happen after Screening if your child/ward cannot take part in the study?

We will explain to you and the child why they cannot take part in the study. If the child needs to be taken care of by a nurse or doctor, we will arrange for the child to visit a clinic or hospital.

On the day after Screening, we will drive you and the child back to your community.

What will happen after Screening if your child/ward can take part in the study?

We will ask you and the child to stay in Hohoe for 13 more days.

In each group of 9 children we will first give moxidectin to only 3 children. Once we know what unwanted effects these 3 children have during the first 3 days after they have swallowed the moxidectin tablets, we will give moxidectin tablets to the other children in that group.

Your child will not be able to take ivermectin while they are on the study (for approximately 6 months).

On the day after Screening (for the first 3 children) or 5 days later, we will:

- Repeat some of the examinations we did during Screening before we give moxidectin to the child.
- Ask the child to swallow moxidectin tablets 2 hours before the child has breakfast.
- Repeat again some of the examinations we did during Screening.
- Take a bit of their blood (1.5 milliliters or 1/3 of a teaspoon) 4 times to measure the amount of moxidectin. We will use a special needle called a 'cannula' so the child only needs to have one needle prick and we will use the special cream, so they won't feel much pain.
- Ask you and the child several times to tell us if the child is feeling unwell or has any pain. If the child has pain or is feeling unwell, we will examine them to find out what we can do to make them feel better. If necessary, we will give the child medicine.
- We will also ask you and the child to tell us immediately if the child is not feeling well. Even during the night, there will always be a nurse to talk to.

During the next 7 days, we will:

- Repeat some of the examinations we did during Screening.
- Take a bit of blood (1.5 milliliters or 1/3 of a teaspoon) on 2 days to measure the amount of moxidectin.
- Take a bit more blood (3.5 milliliters total or 2/3 teaspoon) on the last day to measure the amount of moxidectin and test how well some of the organs in the child's body work.

If the child is feeling well, we will drive you both back to your community on the 7th day after the child has taken the moxidectin tablets. If not, we will ask you and the child to stay longer until the child feels well.

2 weeks, 1 month, 3 months and around 6 months after the child has swallowed the moxidectin tablets, we will:

- Drive the child and one parent or guardian to Hohoe again.
- Each time we will ask you and the child to tell us about any health problems the child has had and any medicine they have taken since we last saw the child.
- Each time we will repeat some of the examinations we did the last time your child or ward was in Hohoe.
- We will take a bit of the child's blood (1.5 milliliters or 1/3 of a teaspoon) on 2 visits to measure the amount of moxidectin.
- We will take a bit more of your child's or ward's blood (3.5 milliliters or 2/3 teaspoon) on 1 visit to measure the amount of moxidectin in it and to find out how some of the organs in the child's body work.
- Each time, the accompanying parent or guardian and the child will spend one night with us and we will give you free meals.

What happens when your child/ward is in Hohoe during school time?

There will be a teacher in our study center in Hohoe. The teacher will help the children keep going with schoolwork, to help make up for the school time they miss.

What should you do if your child/ward is not feeling well when you are back in your community?

You should contact us, either through the community coordinator or directly, or you should take the child to the closest health clinic. The “community coordinator” is the person chosen by your community to contact us whenever you want. We have written down the name for you.

We will make sure that the child is examined and receives medicine if they need it. If needed, we will drive the child or ward and one parent or guardian to the study center in Hohoe or a health clinic or hospital.

Are there any potential advantages of taking part in this study?

The child may not have any advantages.

- The child will have examinations of their health. These examinations are often used by doctors and nurses to find out whether people are sick.
- If the child has the young oncho worms in their skin, moxidectin will cause most or all of the young worms to die and prevent new young worms in the skin for up to about one year.
- If child has health problems because of the young oncho worms in the skin or eyes, these will become better or even go away for up to about 1 year.

Are there any possible risks of taking part in this study?

Unwanted effects of taking moxidectin

Almost all medicines cause unwanted effects.

We have already told you about the unwanted effects we found in people with the oncho worm who took moxidectin or ivermectin. In most cases, these unwanted effects were mild, went away on their own and lasted less than a week. Please let me know if you would like us to look at the pictures again.

Your child may have none, some or all of these unwanted effects. They may be mild, moderate or severe. They may occur soon after the child has taken moxidectin or many hours or days later.

Also, because moxidectin is a new medicine, there may be unwanted effects that we don’t know about yet and we don’t know whether they would be mild, moderate or severe or how long they may last. If we learn about new unwanted effects that may make you change your

mind about your child or ward taking part in this study, we will tell you and the child immediately.

Unwanted effects of taking blood

Having blood taken may cause some pain, bruising, and a little bit of bleeding. Sometimes, it can also cause minor infection (where germs can get into the needle prick) or fainting. This has never happened in our studies because we get the children to lie down and clean the skin to remove any germs before we take blood. If your child or ward has any unwanted effects, they can easily be treated.

What happens to the blood we take?

We will send the blood for measuring the amount of moxidectin to people in another country (the United States of America) to make the measurements. We will do the other tests in our country.

We will not write the child's name on the blood samples only a number. We call this number the child's 'participant number'. Only we know which number belongs to which child who takes part in our study.

We will only use the blood for measuring the amount of moxidectin and for the tests we have told you about. Once these measurements and tests are done, any blood left over will be destroyed.

Who will know what we found out about the child's health during the study?

We will keep the information we collect about your child's or ward's health in what we call "records". We will keep these records private (confidential). This means that we will not share the child's name with anybody who does not belong to our study team without your permission, except when we must because it is the law.

We will keep all the records in a locked, secure area for at least 25 years and possibly longer. When we don't need them anymore, they will be destroyed.

The people who can see the records, including the child's name, are:

- The doctors, nurses and laboratory staff working in our study team.
- People coming to our study center in Hohoe to make sure that we do things right and follow the law. These are
    - People from the Ghana Food and Drugs Authority, and the United States Food and Drug Administration
    - People from the 'Sponsor'. The Sponsor is Medicines Development for Global Health, who make moxidectin tablets. They are giving us the money and the moxidectin tablets for the study and helping us write reports about what we learn.

  - People from the "Ethics Committees". These are people that have been asked by University of Health and Allied Sciences in Hohoe, the Ghana Health Service or the World Health Organization to make sure we do this study in the right way.

The people who can see the records but NOT the child's name or the names of the child's parents or guardians are:

- People from the Sponsor and working with us and the Sponsor. The Sponsor will keep the records without the child's name for at least 25 years.
- People from the Ghana Food and Drugs Authority, the United States Food and Drug Administration, or the authorities in other countries or at the World Health Organization who look at study results to decide whether moxidectin can be used in Ghana and other countries.
- Other people who want to learn about what happens when children 4 to 17 years of age take moxidectin.

If you change your mind about allowing your child or ward to take part in the study or the child doesn't want to take part anymore, we will stop collecting information. We and the Sponsor will still use the information that we collected before you told us you wanted your child or ward to stop taking part in the study (or your child or ward told us they want to stop taking part in the study). However, if your child/ward has an unwanted effect that has not stopped, we will ask you to let us visit you and your child/ward to find out whether they need any treatment for this effect. You and your child/ward do not have to agree to this.

Will you have to pay anything to take part in this study?

You will not pay anything.

- All examinations and medical care that are part of the study will not cost anything.
- When you and the child stay overnight in Hohoe, you will not pay anything. We will give you free food and transportation by car to and from your home.

Will the parent/guardian coming to Hohoe with the child be compensated for the time spent in Hohoe?

The parent or guardian who goes with the child to Hohoe will be compensated, that means get money to make up for loss of earnings. The amount will be based on the number of nights spent in Hohoe for the study. The amount for each night's stay will be roughly the "by day" earnings of adults in your community, currently forty (40.00) Ghana Cedis per day.

What happens if the child has a permanent injury or health problem because of taking part in this study?

We do not think that anybody will have an injury from the study that will make them permanently sick or die. However, should this happen, the Sponsor's insurance will pay you or the child compensation.

What Happens After the Study?

After the study, the child should take ivermectin when it is distributed in your community.

We will tell you, the child and your community about what we learnt. This will happen after the last children finish the study. We will also tell you what we and the Sponsor plan to do so that the Ghana Health Service and the health services in other countries where people have oncho can decide whether they want to give out moxidectin in the way they are now giving out ivermectin.

Who can you talk to if you want more information before you agree to let your child/ward take part in the study or during the study?

If you want to talk to someone who is not in our study team about any worries about the study, your rights, an injury the child may have suffered in the study, or any other questions, concerns or complaints about the study in the future, please contact:

- The **Administrator of the Research Ethics Committee, Institute of Health Research, University of Health and Allied Sciences** by email at **rec@uhas.edu.gh** or by telephone on **+233 362 196 193**.
- The **Secretary to the Research Ethics Committee, Institute of Health Research, University of Health and Allied Sciences, Mr. Fidelis Anumu**, by telephone on + **233 244 061 270**. If you or your child have any questions, concerns or complaints about the study in the future, you may also contact the Project Administrator later.
- The **Administrative Secretary of the Ghana Health Service Ethics Review Committee, Ms Nana Abena Apatu,** by telephone on **+233 503 539 896** or by writing to The Administrative Secretary, Ghana Health Service Ethics Review Committee, Research and Development Division, Ghana Health Service, P. O. Box M190 Accra Ghana.

If you have any questions about the study or need medical help during the study, please contact the following members of the study team or your community coordinator:

1. **Dr. Nicholas O. Opoku (On-site Principal Investigator)**
   University of Health and Allied Sciences
   School of Public Health Research Centre
   Municipal Hospital Hohoe,
   Telephone**: 03627 22042** or **0244 776668** (mobile)
2. **Dr. Felix Doe (Co-investigator)**
   Hohoe Municipal Health Directorate
   Telephone: **0208 437550** or **0245 118342** (mobile)
3. Your **Community Coordinator**
   Name: ______________________________

Telephone: ______________________________

What if you do not want the child to take part in the study or the child does not want to take part in the study, or if you change your mind about taking part in the study?

It is your and your child's or ward's decision to take part in this study.

You or the child can tell us that the child will stop taking part in the study at any time. You do not have to tell us why you want to stop.

Not taking part in the study or changing your mind will not change the health care you and your family will receive from the Ghana Health Service.

When you tell us that you want the child to stop taking part in the study, or the child wants to stop, we will ask you to let us do final examinations so that we know about the child's health and can tell you what you should look out for after the child leaves the study.

If new information becomes available, we will tell you and your child or ward about it and discuss with you whether you and your child or ward want to continue in the study.

We may decide that the child cannot continue in the study. We will do this if we see that the child is not ready to come to Hohoe or if you or the child do not want us to do the examinations or take the blood samples we told you about, or if we find that it is better for the child's health. If this happens, we will explain the reasons to you and the child.

What do you need to do to let us know that you allow your child/ward to take part in the study and your child or ward also wants to take part?

You should discuss this with your child or ward and other people in your community.

For children 8 to 11 years of age, we have a special document describing the study which we will discuss with them while you and a witness your community has chosen are present. If you and the child agree, we will ask you some questions about the study so we can find out what we need to explain better. Then we will ask you, the witness and your child or ward to sign or thumbprint a form.

If your child or ward is 4 to 7 years of age, we will discuss the study with them using a simpler description of what will happen in the study. We will also do this if the child is older but you and we think they are not mature enough to understand the information in the document for the children aged 7 to 11 years. You and a witness your community has chosen will be present. We will look for signs that the child does not want to take part in the study. Because you know the child better than us, we will also ask you if you think the child is showing signs that they do not want to take part in the study. If your child or ward does not show such signs, we will ask you some questions about the study so we can find out what we need to explain better. Then we will ask you and the witness to sign or thumbprint a form.

Help for your discussion with your child/ward, friends, family and others you want to talk this over with

To help you in your discussions of the study with others, here is an overview of what will happen and where it will happen.

You and your child discuss whether the child wants to take part in the study and you all agree

If you do not agree you don't need to do anything

If you agree, you let us know. We will check whether we need to tell you more about the study. We will ask you and your child (if they are old enough) to sign or thumbprint a form.

Step 1 Screening in your community to ask questions about the health of your child (1-2 days)

If your child cannot take part in Step 2 Screening, we will tell you why and tell you if your child should see a nurse or doctor

If your child can take part in **Step 2 Screening**, your child and one of their parents/guardians will go to Hohoe

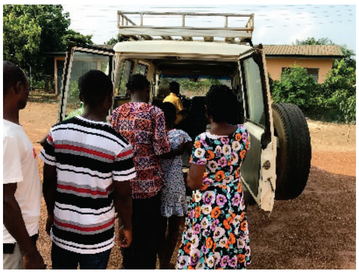

You will stay in our special house with other children who take part in the study and their parents or guardians

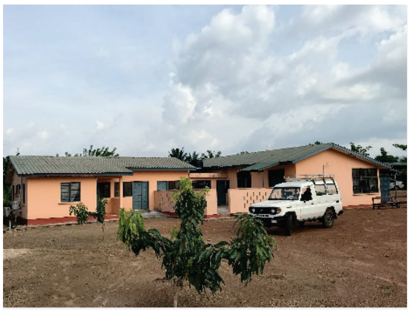

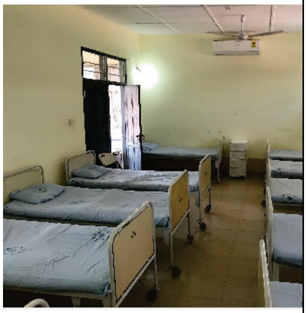

We will ask more questions, do examinations and take bits of blood to learn more about your child's health and find out whether your child can take part in the study (1-2 days)

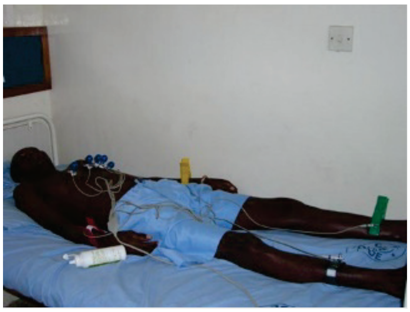
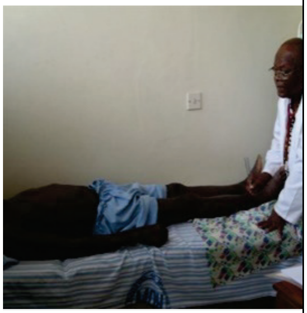
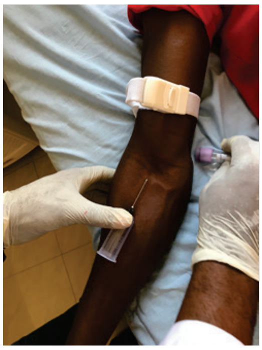
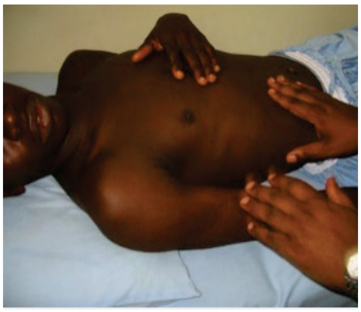
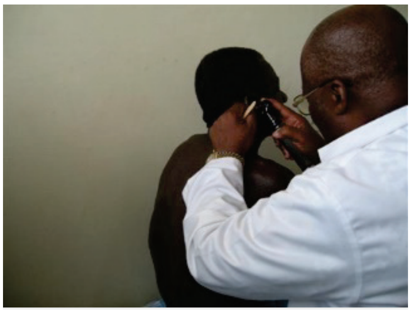

If your child can take part in the study and you still agree, you need to stay in Hohoe for 13 more days.

If your child cannot take part in the study, we will tell you why and take you home. If your child has a health problem, we will arrange a visit to a clinic or hospital near your community.

The next day or 5 days later, we will ask your child to swallow the moxidectin tablets.

On that day and the next 7 days we will take more bits of blood and repeat some of the examinations we did during Screening.

Then we will take you back to your community, unless your child is not well and you stay until your child is well.

2 weeks, 1 month, 3 months and around 6 months after your child swallowed the moxidectin tablets we will bring your child and one parent

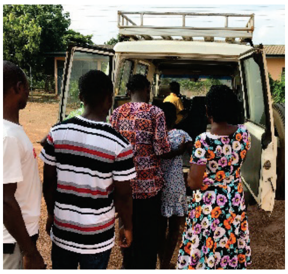
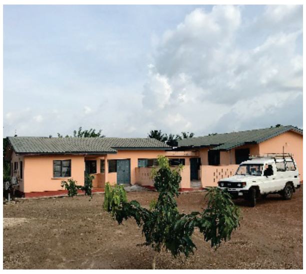
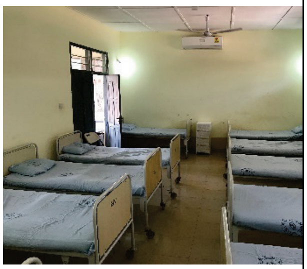

or guardian back to<br>Hohoe.<br>Each time we will repeat some of the examinations we did when your child was in Hohoe last time and on 3 visits we will take a bit of your child's blood.

870

**Assessment of Informed Consent – Questions for Parents/ Guardians of Children 4 to 11 Years**	**Parent/Guardian Response**
Do you understand why your child/ward is being asked to participate in this study?	☐ Yes ☐ No
Have you been able to ask questions and discuss the study?	☐ Yes ☐ No
Questions:	
Do you understand that during Screening we might find that your child/ward cannot take part in the study?	☐ Yes ☐ No
Do you understand that it is your decision to allow your child/ward to take part in this study and that your child/ward must also agree (7 to 11 years) or not show that they don't want to take part (4 to 6 years) (that means your child is a "volunteer")?	☐ Yes ☐ No
Are you willing to allow your child/ward to have all the examinations?	☐ Yes ☐ No
Will we take blood from your child/ward during this study?	☐ Yes ☐ No
How long will your child/ward be in this study?	
Do you understand your child/ward can stop taking part in the study at any time, without having to tell us why?	☐ Yes ☐ No
Is there any charge for being in the study?	☐ Yes ☐ No
Do you understand that this is a study and that we do not know all unwanted effects of moxidectin?	☐ Yes ☐ No
Do you know who to call if you or your child/ward has questions?	☐ Yes ☐ No
Do you know that we will share what we learn about the health of your child/ward with many different people but only those coming to Hohoe to check we do things right can learn the child's name?	☐ Yes ☐ No

**Assessment of Informed Consent – Questions for Parents/ Guardians of Children 4 to 11 Years**	**Parent/Guardian Response**
Are you willing to come to Hohoe five times and so that your child/ward can have the examinations and blood taken that we told you about?	☐ Yes ☐ No

5. Information document for 7- to 11-year-old children

Introduction

My name is ……………………………………………...…. and I am a doctor working at the University of Health and Allied Sciences in Hohoe. I am testing a new medicine called moxidectin in children 4 to 17 years of age. Moxidectin is a new medicine for an illness that you call "oncho".

Because of your age, I want to ask you whether you want to take part in this testing. Testing of a new medicine is called doing "research" or a "study" and means that we want to learn more about the new medicine.

Do you have to take part in the study?

Don't worry, you and your parents or guardian will decide together whether or not you take part in this study. If you don't want to take part, you don't have to, even if your parents or guardian say that you can. You don't have to tell us why you don't want to take part. If you first decide to take part but then change your mind, you just have to tell us. You don't have to tell us why you changed your mind. Nobody will get angry with you.

I will tell you and your parents or guardian now why we want to do this study and what will happen in the study. If I say anything that you don't understand or you have a question, please interrupt me and ask me. You can ask me questions again and again until I have explained in a way that you understand.

Afterwards, you and your parents or guardian and friends can talk about it. Then you and your parents or guardian can let me know whether you want to take part in the study or not.

What is oncho and what is being done now to help people with oncho?

Oncho is caused by a worm that is passed from one person to another through the bites of the small black flies that you see at the riverside and in your community in the mornings and early evenings. The very small young oncho worms in the skin and eyes can make people feel itchy, give them a rash or make them sick in other ways.

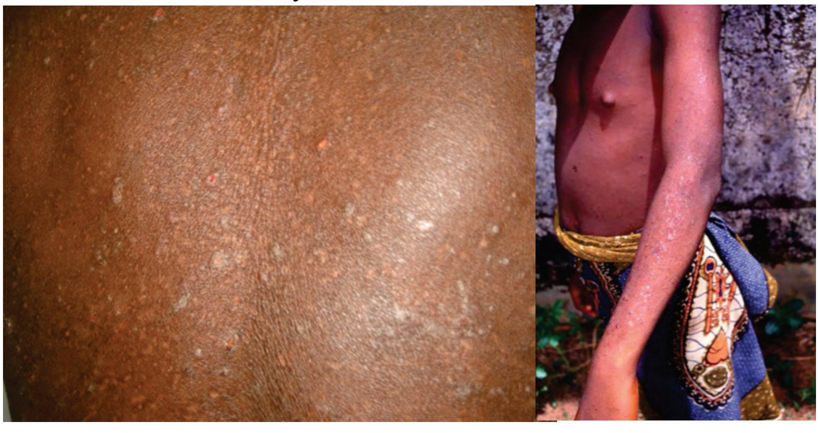

These photos show the skin of people who have a rash because of oncho worms.

Many adults and children in your community may have oncho. Therefore, a medicine called ivermectin is given two times a year to

all people in your community who are at least 5 years of age. Ivermectin kills most of the young oncho worms.

What does moxidectin do?

We have already tested moxidectin in adults and children who were at least 12 years of age. Many of these adults and children were living in your community or in communities nearby. If you or your parents know some of these people, you can ask them about it.

We found out that when people at least 12 years of age take 4 tablets of moxidectin, the young oncho worms in the skin and eyes go away better and for longer than when people take ivermectin.

All medicines have what we call ‘unwanted effects’. For example, some people who take ivermectin may feel more itchy in the first few days after they have taken ivermectin than before they took ivermectin. But afterwards, their itching or itchy rash goes away. We found that when people at least 12 years of age took 4 tablets of moxidectin, some had unwanted effects just like some people have after taking ivermectin.

Why do we want to test moxidectin in children your age?

We want to do this testing to find out the right number of moxidectin tablets for children your age.

How will we find out the right number of moxidectin tablets for children your age?

When somebody takes moxidectin, the moxidectin gets into their blood and from the blood, it kills the young oncho worms in the body. To find the right number of moxidectin tablets for children your age, we need to find out how much moxidectin is in their blood after they swallow moxidectin tablets.

Also, by testing your blood, doctors can tell whether you are sick or getting sick.

To find out how much moxidectin is in your blood and whether you are sick or getting sick, we need to take blood from your arm with a needle. Before we put the needle in your arm, we will put a cream on your arm so that it goes numb and you won’t feel much pain. Here I am showing you the needle and tube I will use and a picture of how I will use them.

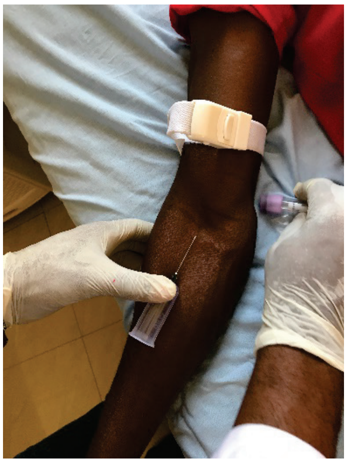

[Note: The Investigator will show the syringe and needle]

To find out whether you have any unwanted effects after taking moxidectin, we will also need to do checks before and after you take moxidectin. None of these checks will hurt. Here are some pictures of these checks.

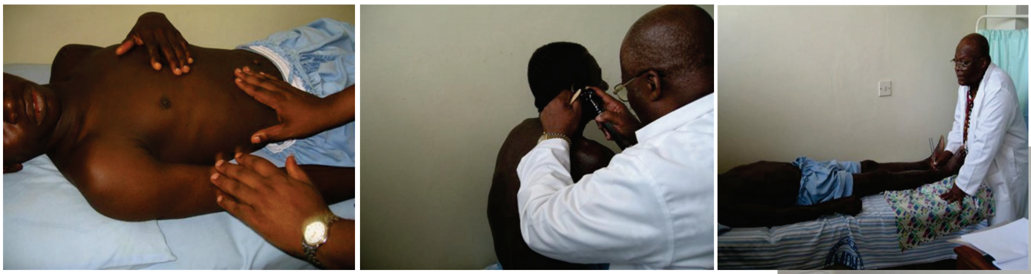

Where and when will this happen?

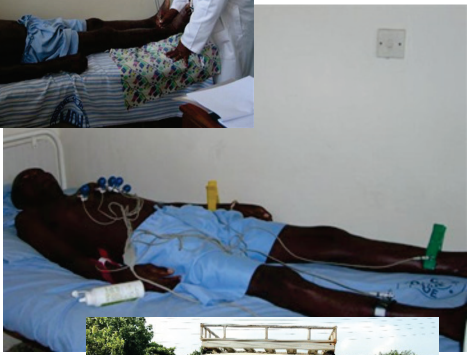

If you and your parents or guardian agree that you can take part, we will first need to learn about your health.

Here in your community, we will ask your parents or guardians about your health. If we learn something about your health that makes us think it is better for you not to take part, we will tell you that you cannot take part in the study.

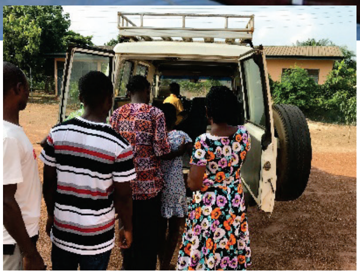

If you are healthy and if you still want to take part, we will take you by car with one of your parents or guardians our place in Hohoe.

Your parent and you will sleep in a special house together with the other children and parents that have come. Here are some pictures.

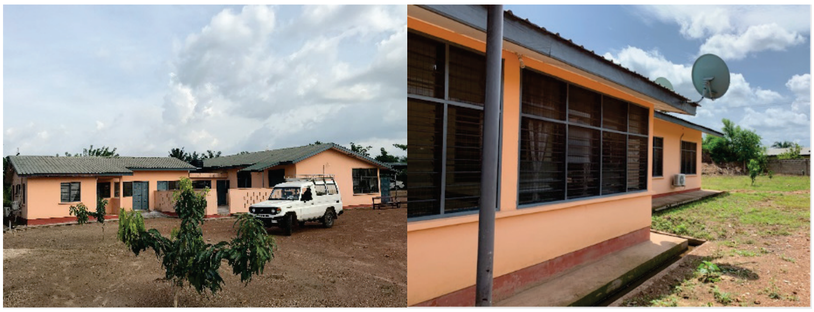

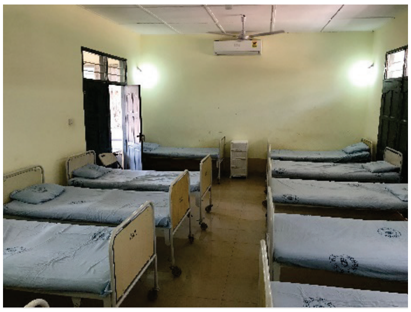

We will take care of your food.

We will do examinations to learn about your health. Your parent or guardian will be with you. We will:

- Weigh you and measure how tall you are.

- Examine your body.
- Measure how hard and fast your heart works and examine how your heart works with a special machine called an "ECG" machine.
- Measure how fast you breathe.
- Find out whether you have a fever.
- Take bits of your blood from your arm with a needle, after we have put a special cream on your arm so you don't feel the needle prick much.

If we find you have a health problem, you cannot take part in the study and we will drive you and your parent or guardian back home. We will tell you and your parent or guardian about the health problem so you can see a doctor or nurse close to your community if you need to do this to get better.

If you can take part in the study, you and your parent will need to stay in Hohoe for between 8 and 13 more days.

We will ask you to swallow up to 4 moxidectin tablets with water.

During the next days, we will take bits of your blood 7 times and we will do the health checks we did before you swallowed the moxidectin tablets. Every day, we will ask you how you are feeling. You and your parent or guardian should also tell us if you don't feel well. We will always be there to talk to you whenever you want to, even at night.

After these 7 days, we will drive you and your parent or guardian home.

There will be 4 more trips from your community to Hohoe for you and your parent or guardian around 2 weeks, 1 month, 3 months and 6 months after you took the moxidectin tablets. Each time, we will do the examinations again and 3 times we will also take bits of your blood. You will stay at least one night each time before we take you home again.

Will you miss school?

You may miss school while you are in Hohoe. We have made sure that there will be a teacher to help you keep going with your schoolwork while you stay in Hohoe.

Will taking blood and swallowing moxidectin make you sick?

We don't think you will get sick during this study. You won't feel much pain when we take bits of your blood, but you might have some pain or get a bruise afterwards.

You might have unwanted effects, that means feel a bit unwell for a few days after you swallow the moxidectin tablets but if this happens, we are there to look after you.

Who will we tell that you took part in the study and what we found out about your health?

We will keep your name and everything we find out about your health private. This means that I will make sure that only the people working with me in Hohoe and people coming to Hohoe to check that we are doing the study the right way can know your name.

We will tell the people who make the moxidectin tablets what we found out about your health, but we will not tell them your name. We will do this so that they can help us find the right number of moxidectin tablets for children your age and then tell the people in our country, and people in other countries who want to learn about moxidectin, what we found in our study. None of these people will know you name.

What can you do if you want to know more before you and your parents/guardians decide whether you can take part in this study?

You can ask me more questions now. In case you want to talk to me or other people who know about this study later, your parents know how to talk to me and others.

How should you tell us that you and your parents/guardians have decided that you can take part in this study?

I will come back to ask you what you and your parents have decided.

If you don’t want to take part in the study, you do not need to do anything.

If you have decided that you want to take part in this study and your parents agree, I will ask you to sign or thumbprint a form. Your parent or guardian and another person chosen by your community will be with you. Before you do this, I will ask you some questions to see whether I need to explain more to you.

**Assessment of Informed Assent – Questions for Children 7-11 Years**	**Child Response**
Do you understand why you are being asked to participate in this study?	☐ Yes ☐ No
Have you been able to ask questions and discuss the study?	☐ Yes ☐ No
Questions:	
Do you understand that during Screening we might find that you cannot take part in the study?	☐ Yes ☐ No
Do you understand that you can only take part if both you and your parents/guardians agree?	☐ Yes ☐ No
Will we take blood during this study?	☐ Yes ☐ No
How long will you be in the study?	
Do you understand you can tell us at any time that you want to stop being in the study, without having to tell us why?	☐ Yes ☐ No
Is there any charge for being in the study?	☐ Yes ☐ No
Do you understand that this is a study and that we do not know all unwanted effects of moxidectin?	☐ Yes ☐ No
Do you know that we will share what we learn about your health with many different people but only those coming to Hohoe to check we do things right can learn your name?	☐ Yes ☐ No
Are you willing to come to Hohoe five times and have the examinations and blood taken that we told you about?	☐ Yes ☐ No

6. Information document for 4- to 6-year-old children and older children considered not mature enough to provide assent

Introduction

My name is …………………………………………………….. and I am a doctor in Hohoe. I want to find out how many tablets of a new medicine for the illness that you call "*oncho*" children should take.

What is oncho?

Oncho is an illness caused by worms so small that you cannot see them with your eyes. The oncho worms can make people itchy, have a rash or make them feel sick. These photos show the skin of people who have a rash because of oncho worms.

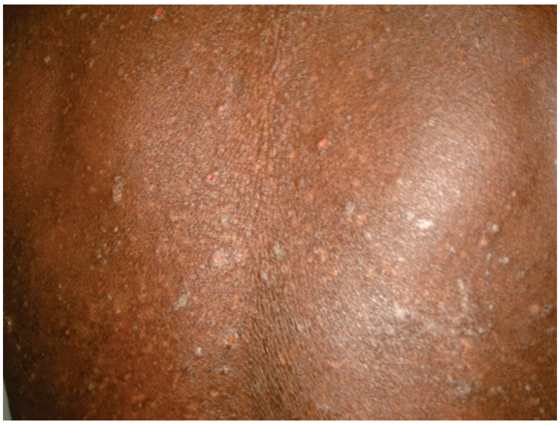

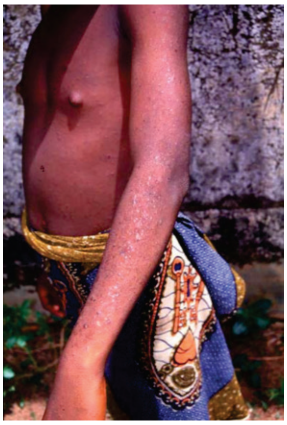

To help people who have the oncho worm, a medicine called ivermectin is given to all children who are 5 years and older and all adults. Have you ever swallowed ivermectin?

What is the new medicine?

The new medicine is called moxidectin (we will call it "moxi" for short). When we gave moxi to children older than you and to adults, we found that it fights the oncho worms better than ivermectin.

I want to find out how many moxi tablets children the same age as you should take. To do this, I am asking you and other children your age to come with one of your parents to Hohoe.

How big are the tablets?

I am showing you here how big a moxi tablet is. The size is about the same as the size of ivermectin tablets. Do you think you can swallow a tablet like this?

[Note – The investigator will show a tablet of similar shape and size to moxidectin tablets. If the child or their parents/guardians do not think the child can swallow the tablet, the information session ends here with a thank you to the child and parents]

When people swallow moxi tablets, moxi gets into their blood and from the blood, moxi kills the oncho worms in the body. Do you know what blood is?

[Note – If not, the investigator will explain what blood is].

To know how many tablets children your age should take, I need to find out how much moxi is in your blood. I will get bits of your blood by putting a needle in your arm.

Please let me examine your arms to see whether it will be easy to take a bit of your blood. This won't hurt.

[Note – If the examination of the arms shows poor venous access, the information session ends here with a thank you to the child and parents]

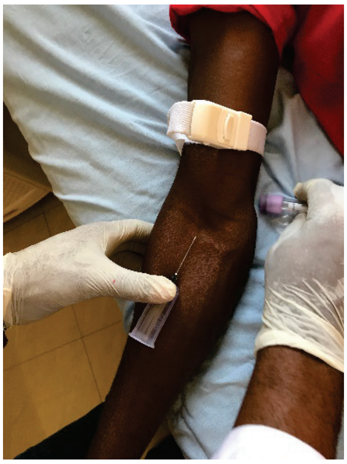

I will put a special cream on your arm so that you don't feel much pain. I am showing you here the needle and the tube I will use to get bits of your blood and a picture of how I will use them.

[Note: The Investigator will show the syringe and needle]

Do you have to come to Hohoe?

Don't worry, you and your parents or guardians will together decide whether you do this. Nobody will get angry with you if you don't want to do this.

I will now tell you what will happen in Hohoe.

If I say anything that you don't understand or you have a question, please interrupt me and ask me. I will explain and if you don't understand what I say, ask me again and again until I have explained in a way that is right for you.

Afterwards, you and your parents or guardians can talk about it.

What if you first think you want to come to Hohoe but later you change your mind?

Don't worry, you just have to tell me or your parents or guardians. Nobody will get angry with you.

What will happen if you want to come to Hohoe?

We will ask your parents to tell us about your health.

If your parents tell us that you have health problems, you will not go to Hohoe since it will be better for you not to do that.

What will happen if you can go to Hohoe?

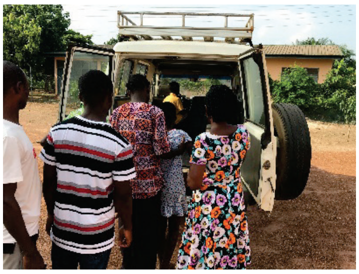

If you do not have health problems, you and one of your parents will ride in a car together with other children and one of their parents to Hohoe.

In Hohoe, you will all stay in our place. You and your parent will sleep in the same room together with the other children and parents who have come. We will give you food.

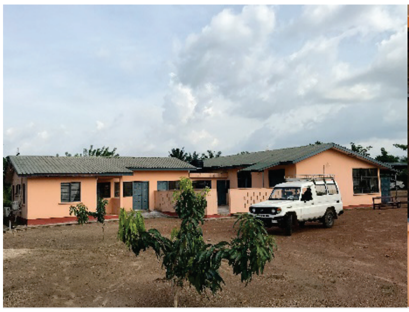
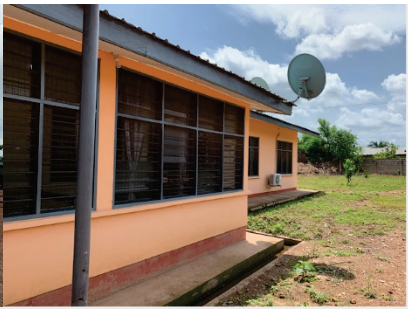

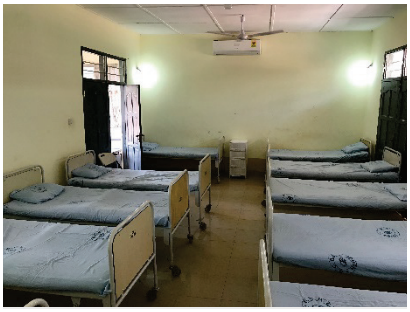

Before we give you tablets to swallow, we will do some checks to find out more about your health. Here are pictures of some of the checks and here are the things that I will put on your body, like in the picture. None of these checks will hurt.

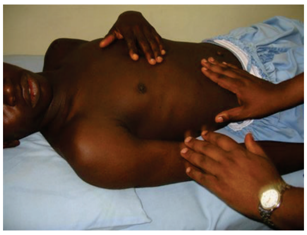
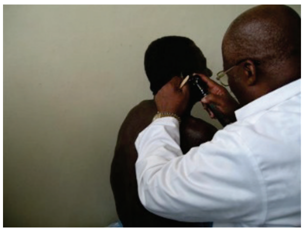
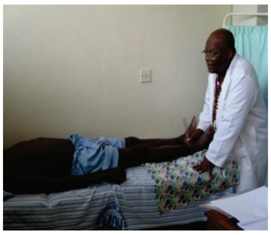
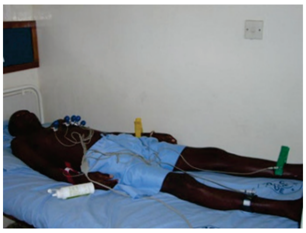

If the checks find you are not healthy, it will be better for you not to swallow the tablets and we will drive you and your parent back to your community in the car.

If you are healthy, we will ask you to swallow the tablets with a lot of water.

After you have swallowed the tablets, you and your parent will stay with us in Hohoe for 7 more days. We will do checks and take bits of your blood like we did before you swallowed the tablets.

Afterwards we will bring you and your parent and the other children and their parents back to your community by car.

We will ask you and your parent to come back to us at Hohoe four more times so we can do more checks and take bits of your blood like we did before you swallowed the tablets.

Will you miss school?

You may miss school while you are in Hohoe. Therefore, we have made sure that there will be a teacher to help you keep going with your schoolwork while you are in Hohoe.

Will the checks and taking bits of your blood and swallowing the tablets make you sick?

We don't think you will get sick. You won't feel much pain when we take bits of your blood, but you might have some pain afterwards. You might feel a bit unwell for a few days after you swallow the tablets but if this happens, we are there to look after you.

7. Consulting room, Offices and study participant accommodation at the University of Health and Allied Sciences (UHAS) Research Centre

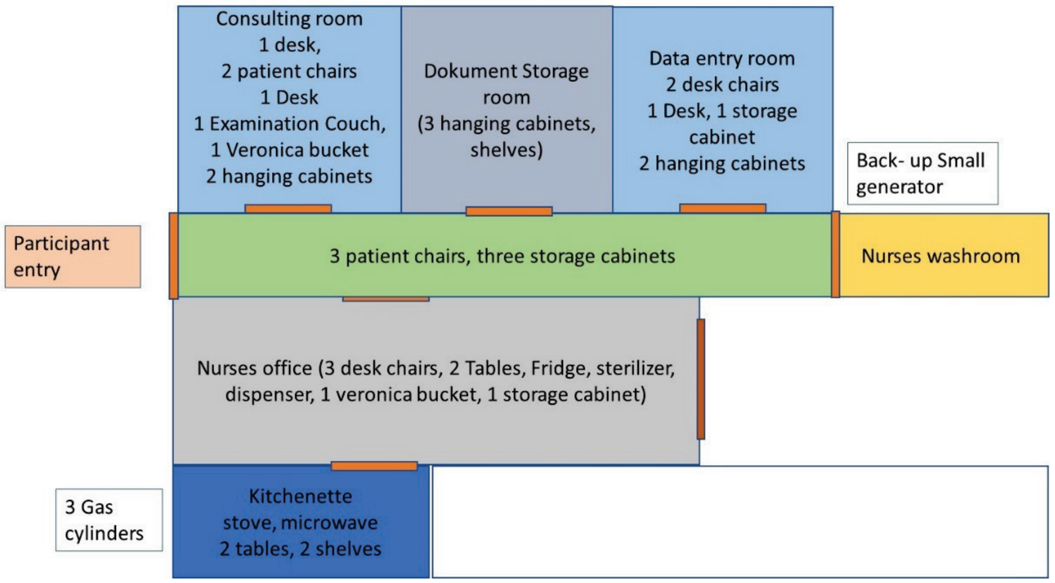

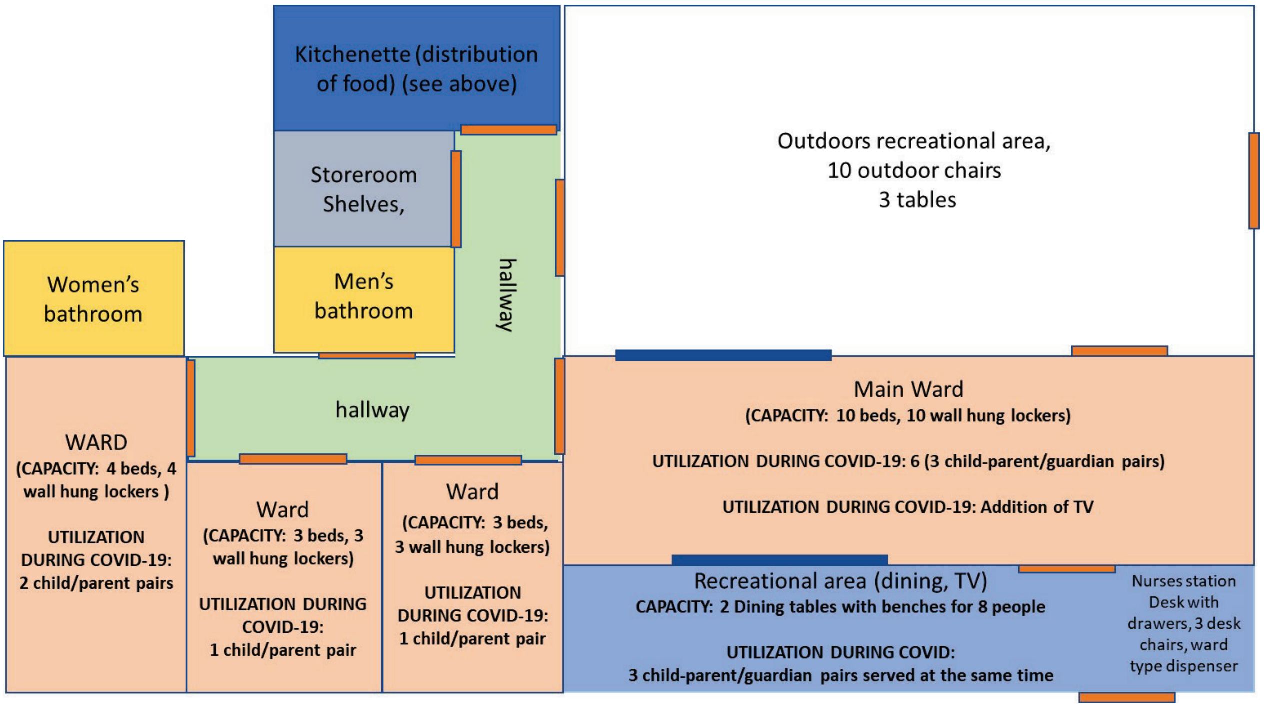

Supplementary information to:

Opoku et al. Identification of a moxidectin dose for 4-to 11-year-old children to support registration and potential use for onchocerciasis elimination: Results of an open-label pharmacokinetic and safety study in an onchocerciasis endemic area in Ghana

8. Overview of timing and evaluations conducted during screening and post-treatment follow-up

Assessment	Assent/Consent & Pre-screening	Screening	On-Study Period													Exit Evaluation
		In-center											Outpatient[i]			
	D-30 to D-1	D-7 to D-1	D0						D1 (H24)	D2 (H48)	D3 (H72)	D7	D14	D28	W12	W24 (or Unscheduled / Early Withdrawal visit)
			Pre-Dose / BL	H0	H1	H2	H4	H8								
Allowable assessment window					±10M	±10M	±30M	±1H	±2H	±2H	±2H	±1D	±2D	±4D	±14D	±28D
Informed consent / assent	X															
Inclusion/exclusion criteria	X	X	X													
Medical history	X	X														
Physical examination [a]		X														
Targeted physical examination [a]			X		(X)	(X)	(X)	(X)	X	X	X	X	X	X	X	X
Vitals signs [b]		X	X		X	(X)	(X)	X	X	X	X	X	X	X	X	X
Height		X														
Body weight		X												X		X [j]
Arm circumference		X														
12-lead ECG		X			(X)	(X)	(X)	(X)	(X)	(X)	(X)	(X)	(X)	(X)	(X)	(X)
Haematology [c,g]		X										X		X	X	(X)
Clinical chemistry [c,g]		X										X		X	X	(X)
Pregnancy test [d]		X												X	X	X [j]
Pharmacokinetic blood sampling [g]		X			X	X	X	X	X		X	X	X	X	X	
Screening for co-infections [f]		X														
Moxidectin administration [h]				X												
Adverse events			X<--->X													
Concurrent medications			X<--->X													

Abbreviations: X = required; (X) = as clinically indicated; M = minutes; H = hour; D = day; W = Week; ECG = electrocardiogram; BL = Baseline.

a. Full physical examination (PE) at Screening, targeted PE (informed by concurrent conditions, signs, symptoms and adverse events) at all subsequent time points.
b. Blood pressure and pulse rate will be measured semi-supine. Unscheduled recordings will be performed if clinically indicated.
c. Blood samples (approx. 2 mL total) for haematology and biochemistry testing will be drawn within 3 days prior to Day 0 and results available prior to dosing.
d. Serum pregnancy testing will be conducted at Screening and urine pregnancy testing at all other time points for girls of child-bearing potential (including at unscheduled visits as clinically indicated).
f. Testing for Human Immunodeficiency Virus (HIV), hepatitis B, and hepatitis C. *Loa loa* testing only for participants with a history of residence in a loiasis endemic area.
g. Application of topical local anaesthetic is recommended prior to obtaining blood samples via cannula or needle.
h. Study drug dosing will be after an overnight fast. Breakfast may be given 2 hours after dosing (and following the H2 blood sample collection).
i. Participants and one parent/guardian will be brought back to the Research Center
j. Not required at unscheduled visits unless clinically indicated.

9. Population pharmacokinetic modelling for dose selection in the study and parameter estimates for the initial, the updated and the final model

The population pharmacokinetic (popPK) analyses were performed according to the United States Food and Drug Administration (US FDA) 'Guidance for Industry: Population Pharmacokinetics' (1) and the European Union Guidance on 'Reporting the Results of Population Pharmacokinetic Analyses' (2).

The popPK model was developed using the data from one Phase 1 study in healthy male adults (56/58 white, 1 Indian, 1 Asian) which evaluated the relative bioavailability of a liquid and tablet formulation of moxidectin (10 mg dose, N=29 for each formulation) (3), one Phase 1 study in healthy male adults (52/54 white, 1 black, 1 mixed race) with the tablet formulation which evaluated the effect of food on moxidectin pharmacokinetics (PK) (8 mg dose, N=27 in the fasted and in the fed arm) (4), a third Phase 1 study conducted in healthy male adults (27 white, 29 black, 4 other) with the tablet formulation (doses 4 mg, 8 mg, 16 mg, 24 mg, 36 mg, N=10 for each dose level) (5) and the data from a Phase 2 study in *O. volvulus* infected African adults (71 men, 27 women) who received the tablet formulation (doses 2 mg N=32, 4 mg N=34, 8 mg N=31) (6). Fig S 1 shows the moxidectin plasma concentration-time profiles for each adult in these four studies together with those obtained in this study.

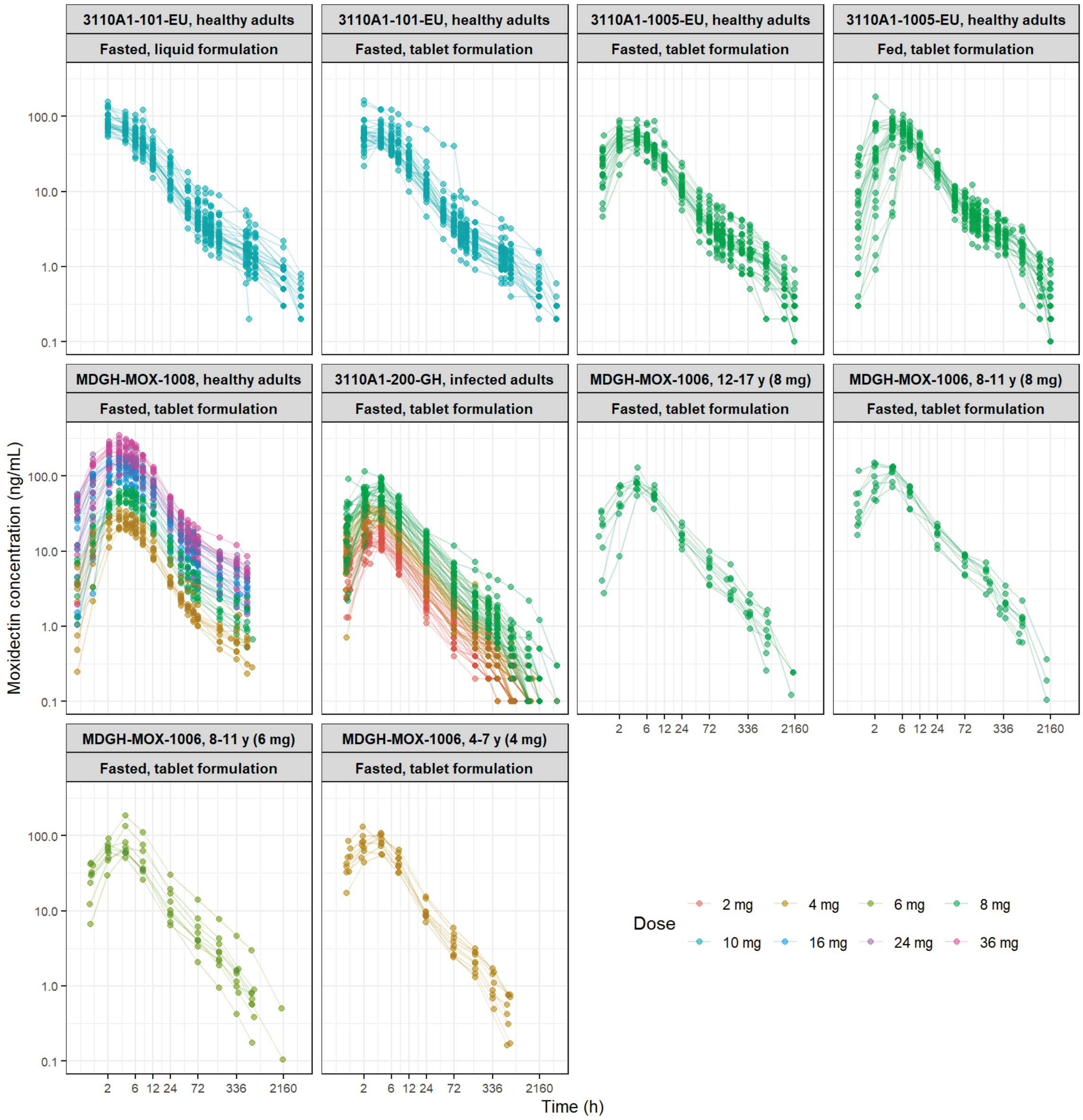

Fig S 1: Individual moxidectin plasma concentrations in each adult in the studies which contributed to the development of the popPK model and in the participants in study MDGH-MOX-1006

3110A1-101-EU: concentrations in healthy male volunteers after administration of 10 mg moxidectin as a liquid formulation of moxidectin (N=29 for each formulation) (3); 3110A1-1005-EU: concentrations in healthy male volunteers after administration of 8 mg moxidectin as a tablet formulation fasted (N=27) or fed (after a high fat breakfast (N=27) (4); MDGH-MOX-1008: concentrations in healthy male volunteers who received 4 mg, 8 mg, 16 mg , 24 mg or 36 mg as a tablet formulation (N=10 per dose level); 3110A1-200-GH: concentrations in *O. volvulus* infected adults who received 2 mg (N=32), 4 mg (N=34) or 8 mg (N=31) moxidectin in tablet formulation (6).

The model (Fig S 2) comprises a 2-transit absorption process with 3-compartment disposition and first-order elimination. Effects of food and formulation are included on the absorption parameters for mean transit time (*MTT*) and relative bioavailability. The model accounts for the effects of sex and body mass index (BMI) on the apparent inter-compartmental clearance between the central and second peripheral compartments (CLd_2/F), and the apparent volume of distribution of the second peripheral compartment (V_{p2}/F), specifically larger estimates of both peripheral parameters in women relative to men, and increased V_{p2}/F in individuals with higher BMI (stratified by Phase 1 and Phase 2). For all parameters, the model differentiated healthy (Phase 1) effects from those in *O. volvulus* infected individuals.

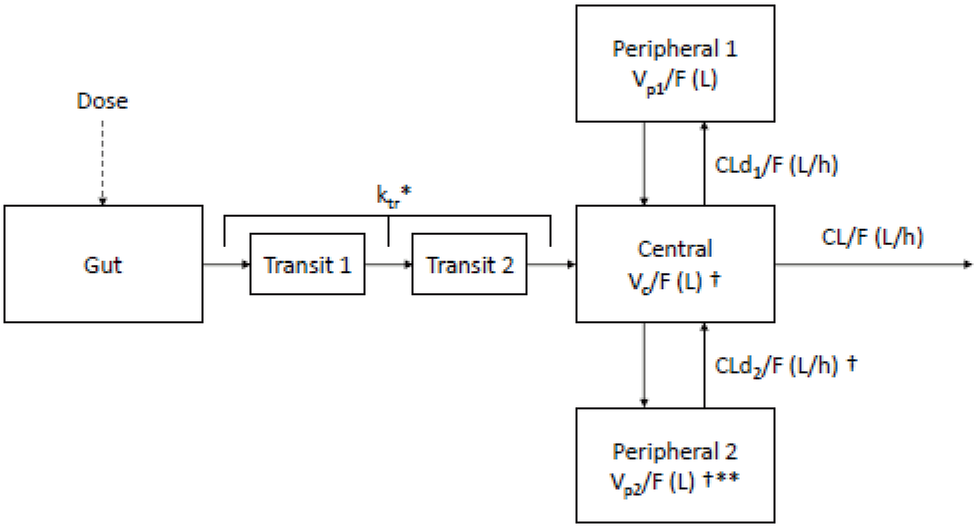

Fig S 2: Schematic of the moxidectin PopPK model

k_{tr}=transit rate constant; CL/F=apparent clearance; CL_{d1}/F=apparent inter-compartmental clearance between the central and first peripheral compartments; CL_{d2}/F=apparent inter-compartmental clearance between the central and second peripheral compartments; MTT=mean transit time V_c/F=apparent volume of distribution of the central compartment; V_{p1}/F=apparent volume of distribution of the first peripheral compartment; V_{p2}/F=apparent volume of distribution of the second peripheral compartment.
k_{tr}* = 3/MTT, † Allometry imposed in the original model based on fixed theoretical exponents. These were replaced by data-derived exponents during the model updates, ** Relative bioavailability study design effect (fractional change) incorporated (3)

In the initial model, body weight scaling was incorporated using fixed theoretical exponents (0.75 and 1.0 for clearance and volume terms, respectively) on V_c/F, CLd_2/F, and V_{p2}/F. The data did not support inclusion of allometric body weight effects on the apparent clearance (*CL/F*) in the model.

Re-fitting the initial model (built on the data from adults with a weight range of 42.7 to 107 kg) after inclusion of the data from Cohort I and Cohort II treated with 8 mg into the dataset (weight range 22.6 to 55.4 kg) suggested that the initial model predicted moxidectin clearance in adolescents and children aged 8 to 17 years to be faster than indicated by the data. Incorporating body weight on *CL/F* was sufficient to obtain a good fit of model-predicted and observed moxidectin concentrations. This fit was further improved when the allometric exponents were estimated, and not fixed to theoretical values. Given the high correlation between weight and BMI, BMI was dropped from the model as a covariate.

Once the data from Cohort II treated with 6 mg and Cohort III treated with 4 mg were available (weight range 13.6 to 31.4 kg), refitting the model to the complete set of data (now covering a weight range of 13.6 kg to 107 kg) resulted in updates to the weight exponents on apparent clearance and volume of distribution parameters for the central and the second peripheral compartment and addition of weight exponents for clearance and volume of distribution for the first peripheral compartment.

Table S 1 shows the parameter estimates for the initial model, the model updated after the availability of plasma moxidectin concentrations up to 28 days after dosing of Cohort I and Cohort II with 8 mg, and the final model emerging after availability of the data from Cohort II dosed with 6 mg and Cohort III dosed with 4 mg.

Table S 1: Parameter estimates for the initial moxidectin PopPK model, the updated model and the final model after incorporation of all data

	**Initial Model** For tablet or liquid formulation administered in fasted or fed state based on data from 3 studies in healthy and 1 in *O. volvulus* infected adults				**Updated model** For tablet formulation administered in fasted state After incorporation of data from Cohorts I and II – 8 mg				**Final Model** For Tablet formulation administered in fasted state After incorporation of data from Cohort II – 6 mg and III			
**Parameter**	**Est.**	**%RSE**	**BSV**	**%RSE**	**Est.**	**%RSE**	**BSV**	**%RSE**	**Est.**	**%RSE**	**BSV**	**%RSE**
MTT (h)	1.70	4.13	0.447	12.1	1.71	2.93	0.446	10.9	1.69	3.76	0.439	11.0
CL/F (L/h)	3.65	5.81	0.429	10.5								
CL/F (L/h/70kg)					3.61	4.21	0.418	10.4	3.86	6.08	0.420	9.57
V_c/F (L/70 kg)	127	4.39	0.236	14.3	119	3.4	0.227	13.9	120	4.71	0.224	14.2
CL_{d1}/F (L/h)	5.03	5.05										
CL_{d1}/F (L/h/70kg)					4.66	4.04			5.45	5.66		
V_{p1}/F (L)	216	6.34										
V_{p1}/F (L/70kg)					208	15.3			226	7.29		
CL_{d2}/F (L/h/70 kg)	3.25	6.15	0.336	12.4	3.55	11.6	0.319	20.6	3.52	6.73	0.323	11.9
V_{p2}/F (L/70 kg)	1086	5.17	0.422	9.51	1190	7.74	0.439	15.7	1140	5.85	0.448	10.0
Correlation (*CL/F, Vc/F*)	0.619				0.607				0.614			
Correlation (*CLd2/F, Vp2/F*)	0.685				0.632				0.632			
Phase 1 effect* on *CL/F*	0.689	19.6			0.708	16.5			0.634	17.4		
Phase 1 effect* on V_c/F	0.800	23.2			0.883	45			0.866	40.2		
Phase 1 effect* on CL_{d1}/F	0.837	36.3			0.899	59.3			0.722	22.8		
Phase 1 effect* on V_{p1}/F	0.515	11.7			0.553	36.6			0.484	12.9		
Phase 1 effect* on CL_{d2}/F	1.76	13.4			1.50	29.9			1.51	21.6		
Phase 1 effect* on V_{p2}/F	1.27	35.1			1.25	43.7			1.31	33.7		
Food effect* on *MTT*	2.09	10.4			2.05	17.2			2.06	10		
Food effect* on relative bioavailability***	1.36	17.0			1.36	13.6			1.33	15.9		
Formulation effect* on *MTT* (fixed)	0.292				0.292				0.292			
Formulation effect* on relative bioavailability***	1.18	14.3			1.18	36.4			1.18	13.1		
Study 3110A1-101-EU*** effect* on *MTT* (fixed)	0.477				0.477				0.477			
Study 3110A1-101-EU*** effect* on V_{p2}/F	1.19	40.8			1.23	32.9			1.26	32.8		
Study 3110A1-101-EU effect* on proportional error	0.900	16.8			0.892	106			0.905	19.8		
Sex effect* on CL_{d2}/F	1.48	25.1			1.53	20.5			1.53	20.5		

	**Initial Model** For tablet or liquid formulation administered in fasted or fed state based on data from 3 studies in healthy and 1 in *O. volvulus* infected adults				**Updated model** For tablet formulation administered in fasted state After incorporation of data from Cohorts I and II – 8 mg				**Final Model** For Tablet formulation administered in fasted state After incorporation of data from Cohort II – 6 mg and III			
**Parameter**	**Est.**	**%RSE**	**BSV**	**%RSE**	**Est.**	**%RSE**	**BSV**	**%RSE**	**Est.**	**%RSE**	**BSV**	**%RSE**
Sex effect* on V_{p2}/F	1.61	21.9			1.83	20.7			1.83	16.6		
Proportional error (SD; %)	21.5	0.785			21.7	4.78			21.4	0.762		
Phase 1 BMI effect** on V_{p2}/F	1.69	18.3										
Phase 2 BMI effect** on V_{p2}/F	0.586	52.4										
Weight exponent on CL/F					0.445	22.3			0.460	25.5		
Weight exponent on V_c/F					0.689	11.5			0.748	11.3		
Weight exponent on CL_{d2}/F					1.26	8.09			1.28	14.3		
Weight exponent on V_{p2}/F					1.79	10.0			1.5	10.2		
Weight exponent on CL_{d1}/F									0.554	17		
Weight exponent on V_{p1}/F									0.226	63.4		

Est. = estimate, BMI = Body mass index, BSV = Between-subject variance, SD = Standard deviation; CL/F = apparent clearance; CLd_1/F = apparent inter-compartmental clearance between the central and first peripheral compartments; CLd_2/F = apparent inter-compartmental clearance between the central and second peripheral compartments; CV = coefficient of variation; MTT = mean transit time; V_c/F = apparent volume of distribution of the central compartment; SD = standard deviation; V_{p1}/F = apparent volume of distribution of the first peripheral compartment; V_{p2}/F = apparent volume of distribution of the second peripheral compartment, %RSE = Percent relative standard error.

Note: Given the correlation between BMI and weight, BMI was not included in the model once data-derived weight exponents were included in the models. For fixed parameters %RSE is not applicable

*Fractional change; **Exponent from power function; *** Study 3110A1-101-EU was a relative bioavailability study in which 29 healthy adults received 10 mg moxidectin as a liquid formulation and 29 received 10 mg moxidectin as a tablet formulation.

Notes: RSE of parameters was calculated as 100 × (standard error (SE)/typical value), BSV was calculated as √(exp[variance]-1), CV for BSV estimates was based on the estimated variances, and ETA shrinkage (%) was 100×(1-SD(ETA)/√[variance]).

10. Additional information on evaluation of safety of moxidectin and results

Table S 2: Criteria for assessing the causal relationship of an adverse event (AE) to moxidectin administration

Causality	Comment
Unrelated	AE is clearly due to extraneous causes (e.g. underlying disease, environment, known effect of another drug)
Unlikely	The temporal association between the AE and study drug is such that study drug is not likely to have any reasonable association with the AE
Possible	The AE could have been produced by the subject's clinical state or study drug
Probable	The AE follows a reasonable temporal sequence from the time of study drug administration, abates upon discontinuation of the study drug and cannot be reasonably explained by the known characteristics of the subject's clinical state
Definite	The AE follows a reasonable temporal sequence from the time of study drug administration, abates upon discontinuation of the study drug and/or reappears when study drug is re-introduced

Table S 3: All adverse events recorded after moxidectin administration by system organ class and preferred term

	**Cohort I 12-17 yrs**	**Cohort II 8-11 yrs**		**Cohort III 4-7 yrs**	
**System Organ Class Preferred Term**	**MOX 8 mg (N=9) n (%) m**	**MOX 8 mg (N=9) n (%) m**	**MOX 6 mg (N=9) n (%) m**	**MOX 4 mg (N=9) n (%) m**	**TOTAL (N=36) n (%) m**
**All AEs**	**4 (44.4) 4**	**6 (66.7) 10**	**7 (77.8) 11**	**7 (77.8) 13**	**24 (66.7) 38**
**Infections and infestations**	**3 (33.3) 3**	**6 (66.7) 8**	**6 (66.7) 9**	**6 (66.7) 8**	**21 (58.3) 28**
Malaria	1 (11.1) 1	4 (44.4) 6	5 (55.6) 7	4 (44.4) 6	14 (38.9) 20
Upper respiratory tract infection	1 (11.1) 1	1 (11.1) 1	1 (11.1) 1	0 (0.0) 0	3 (8.3) 3
Conjunctivitis	0	1 (11.1) 1	1 (11.1) 1	0	2 (5.6) 2
Abscess limb	0	0	0	1 (11.1) 1	1 (2.8) 1
Hookworm infection	0	0	0	1 (11.1) 1	1 (2.8) 1
Tinea versicolour	1 (11.1) 1	0	0	0	1 (2.8) 1
**Gastrointestinal disorders**	**0**	**2 (22.2) 2**	**2 (22.2) 2**	**3 (33.3) 3**	**7 (19.4) 7**
Abdominal pain	0	2 (22.2) 2	0	1 (11.1) 1	3 (8.3) 3
Diarrhoea	0	0	2 (22.2) 2	1 (11.1) 1	3 (8.3) 3
Vomiting	0	0	0	1 (11.1) 1	1 (2.8) 1
**Blood and lymphatic system disorders**	**0**	**0**	**0**	**1 (11.1) 1**	**1 (2.8) 1**
Anaemia	0	0	0	1 (11.1) 1	1 (2.8) 1
**Injury, poisoning and procedural complications**	**0**	**0**	**0**	**1 (11.1) 1**	**1 (2.8) 1**
Skin laceration	0	0	0	1 (11.1) 1	1 (2.8) 1
**Musculoskeletal and connective tissue disorders**	**1 (11.1) 1**	**0**	**0**	**0**	**1 (2.8) 1**
Neck pain	1 (11.1) 1	0	0	0	1 (2.8) 1

AE: adverse event, N: number of subjects; n: subject count; m: event count.
Notes: A higher number of events than subjects is due to the fact one subject may have had the same preferred term event twice or two events in the same System Organ Class. Differences in the total number of AEs relative to the results posted on Clinicaltrials.gov are due to the fact that the results posted include a 'false positive investigation result'.

Supplementary information to:

Opoku et al. Identification of a moxidectin dose for 4-to 11-year-old children to support registration and potential use for onchocerciasis elimination: Results of an open-label pharmacokinetic and safety study in an onchocerciasis endemic area in Ghana

11. Moxidectin plasma concentrations and pharmacokinetic parameters

Table S 4: Summary statistics of plasma moxidectin concentrations by cohort and time point

Cohort Visit-Time Point	Statistics								
	**n**	**n BLQ**	**Mean (SD) (ng/mL)**	**CV (%)**	**Median (ng/mL)**	**Minimum (ng/mL)**	**Maximum (ng/mL)**	**GM (GSD) (ng/mL)**	**GCV (%)**
**Cohort I: 8 mg Oral 12 to 17 yr (N=9)**									
Pre-dose	9	9	N/A	N/A	BQL	BQL	BQL	N/A	N/A
1 hr post-dose	9	0	19.2 (11.6)	60.4	21.8	2.76	34.6	14.6 (2.48)	114
2 hr post-dose	9	0	48.5 (22.2)	45.8	42.7	8.57	76.0	41.8 (1.96)	75.6
4 hr post-dose	9	0	81.5 (21.3)	26.1	79.3	54.0	129	79.3 (1.28)	24.8
8 hr post-dose	9	0	55.3 (11.1)	20.1	54.2	36.7	75.7	54.3 (1.23)	20.6
Day 1 post-dose	9	0	16.2 (4.33)	26.8	16.2	10.6	24.1	15.7 (1.30)	26.6
Day 3 post-dose	9	0	6.19 (2.13)	34.4	6.20	3.47	9.86	5.85 (1.44)	37.4
Day 7 post-dose	9	0	3.58 (1.42)	39.6	3.05	2.21	6.63	3.36 (1.44)	37.8
Day 14 post-dose	9	0	1.66 (0.518)	31.2	1.48	0.928	2.63	1.59 (1.36)	31.7
Day 28 post-dose	9	0	0.864 (0.457)	52.9	0.737	0.255	1.64	0.751 (1.80)	64.1
Week 12 post-dose	9	5	N/A	N/A	BQL	BQL	0.243	N/A	N/A
**Cohort II: 8 mg Oral 8 to 11 yr (N=9)**									
Pre-dose	9	9	N/A	N/A	BQL	BQL	BQL	N/A	N/A
1 hr post-dose	9	0	44.2 (31.3)	70.7	33.0	16.2	117	36.9 (1.85)	67.8
2 hr post-dose	9	0	92.9 (40.5)	43.6	80.5	46.6	149	85.1 (1.57)	47.5
4 hr post-dose	9	0	114 (22.4)	19.6	123	70.7	133	112 (1.25)	22.7
8 hr post-dose	9	0	58.3 (14.0)	24.1	61.7	35.9	72.8	56.6 (1.31)	27.7
Day 1 post-dose	9	0	17.7 (4.24)	23.9	17.7	10.7	23.1	17.2 (1.30)	26.9
Day 3 post-dose	9	0	7.43 (1.73)	23.2	8.31	4.74	9.03	7.23 (1.30)	26.3
Day 7 post-dose	9	0	4.57 (1.45)	31.7	4.26	2.63	7.07	4.36 (1.39)	33.5
Day 14 post-dose	9	0	2.04 (0.680)	33.4	1.96	1.29	3.49	1.95 (1.36)	31.4
Day 28 post-dose	9	0	1.13 (0.487)	43.0	1.11	0.604	2.20	1.05 (1.51)	42.8
Week 12 post-dose	9	6	N/A	N/A	BQL	BQL	0.360	N/A	N/A
**Cohort II: 6 mg Oral 8 to 11 yr (N=9)**									
Pre-dose	9	9	N/A	N/A	BQL	BQL	BQL	N/A	N/A
1 hr post-dose	9	0	28.8 (12.7)	44.1	30.5	6.68	42.9	25.1 (1.87)	69.5
2 hr post-dose	9	0	61.5 (18.1)	29.4	61.8	29.7	91.6	58.8 (1.39)	33.7
4 hr post-dose	9	0	84.8 (45.9)	54.1	63.5	50.1	187	76.8 (1.55)	46.2
8 hr post-dose	9	0	50.9 (27.5)	53.9	36.4	25.6	111	45.8 (1.60)	49.6
Day 1 post-dose	9	0	13.4 (7.57)	56.5	10.1	6.43	29.8	11.9 (1.66)	54.4
Day 3 post-dose	9	0	5.59 (3.57)	63.8	4.07	2.07	14.0	4.84 (1.73)	59.2
Day 7 post-dose	9	0	3.16 (1.97)	62.4	2.73	0.937	7.74	2.71 (1.80)	63.9
Day 14 post-dose	9	0	1.59 (1.22)	76.9	1.46	0.422	4.65	1.30 (1.91)	72.4
Day 28 post-dose	9	0	0.871 (0.826)	94.8	0.669	0.173	2.99	0.661 (2.13)	88.0
Week 12 post-dose	9	7	N/A	N/A	BQL	BQL	0.497	N/A	N/A
**Cohort III: 4 mg Oral 4 to 7 yr (N=9)**									
Pre-dose	9	9	46.6 (20.2)	43.4	42.1	17.4	85.0	42.6 (1.59)	48.9
1 hr post-dose	9	0	77.0 (26.2)	34.0	74.5	44.3	131	73.3 (1.39)	34.0
2 hr post-dose	9	0	82.8 (19.2)	23.2	79.7	55.9	108	80.7 (1.28)	24.7
4 hr post-dose	9	0	45.3 (11.3)	24.8	42.8	32.0	64.8	44.1 (1.28)	24.9
8 hr post-dose	9	0	10.0 (2.87)	28.6	8.86	6.99	15.4	9.72 (1.30)	26.4
Day 1 post-dose	9	0	3.68 (1.20)	32.5	3.41	2.38	5.89	3.52 (1.37)	32.5

Supplementary information to:

Opoku et al. Identification of a moxidectin dose for 4-to 11-year-old children to support registration and potential use for onchocerciasis elimination: Results of an open-label pharmacokinetic and safety study in an onchocerciasis endemic area in Ghana

Cohort<br>Visit-Time Point	Statistics								
	n	n BLQ	Mean (SD) (ng/mL)	CV (%)	Median (ng/mL)	Minimum (ng/mL)	Maximum (ng/mL)	GM (GSD) (ng/mL)	GCV (%)
Day 3 post-dose	9	0	2.20 (0.652)	29.7	2.06	1.30	3.12	2.11 (1.37)	32.0
Day 7 post-dose	8	0	1.07 (0.436)	40.9	0.986	0.492	1.69	0.985 (1.55)	45.8
Day 14 post-dose	8	1	1.07 (0.436)	40.9	0.986	0.492	1.69	0.985 (1.55)	45.8
Day 28 post-dose	9	0	0.511 (0.250)	49.0	0.566	0.163	0.765	0.441 (1.87)	69.4
Week 12 post-dose	9	9	N/A	N/A	BQL	BQL	BQL	N/A	N/A

BLQ: Below limit of quantification (<0.1 ng/mL), GM: Geometric mean, GSD: Geometric SD, GCV: Geometric CV, CV: Coefficient of Variation, NA: Not Applicable, n: Number of observations, N: Number of participants, SD: Standard Deviation.

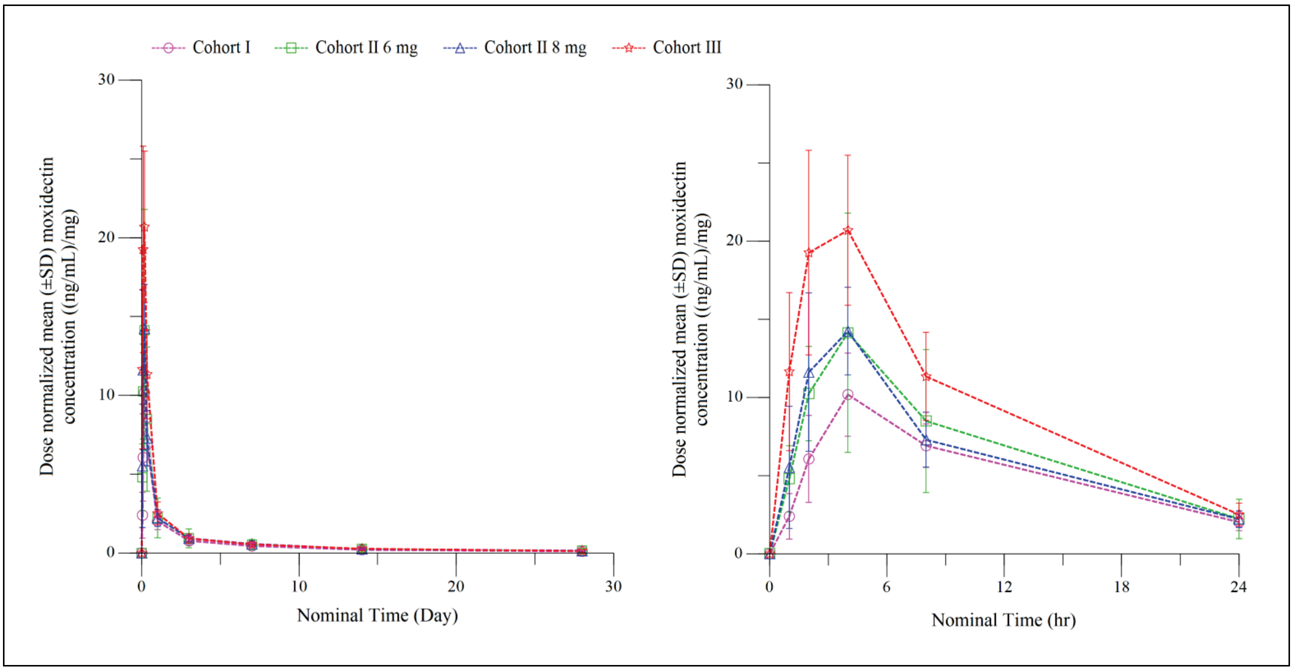

Fig S 3: Dose-normalised mean (± standard deviation) plasma moxidectin concentration-time profiles

Table S 5: Summary statistics of plasma moxidectin pharmacokinetic parameters

Cohort, Dose, Age, N<br>Parameter (unit)	Statistics							
	n	Mean (SD)	CV (%)	Median	Minimum	Maximum	GM (GSD)	GCV (%)
**Cohort I: 8 mg Oral 12 to 17 yr (N=9)**								
C_{max} (ng/mL)	9	84.6 (18.2)	21.5	79.3	67.0	129	83.1 (1.21)	19.1
Dn-C_{max} ((ng/mL)/mg)	9	10.6 (2.28)	21.5	9.91	8.38	16.1	10.4 (1.21)	19.1
t_{max} (hr)	9	-	-	3.98	2.12	8.03	-	-
AUC_{0-24} (hr*ng/mL)	9	948 (122)	12.8	980	744	1120	940 (1.14)	13.3
AUC_{0-48} (hr*ng/mL)	9	1260 (194)	15.4	1270	956	1520	1240 (1.17)	15.9
AUC_{0-72} (hr*ng/mL)	9	1450 (250)	17.3	1430	1070	1820	1430 (1.19)	17.8
AUC_{0-D7} (hr*ng/mL)	9	1900 (402)	21.2	1840	1340	2600	1860 (1.24)	21.5
AUC_{0-D14} (hr*ng/mL)	9	2330 (550)	23.6	2210	1600	3360	2270 (1.26)	23.5
AUC_{0-D28} (hr*ng/mL)	9	2750 (692)	25.1	2600	1880	4060	2680 (1.28)	24.7

	Statistics							
**Cohort, Dose, Age, N Parameter (unit)**	**n**	**Mean (SD)**	**CV (%)**	**Median**	**Minimum**	**Maximum**	**GM (GSD)**	**GCV (%)**
AUC_{0-W12} (hr*ng/mL)	9	3330 (858)	25.8	2980	2480	4730	3240 (1.28)	25.0
$AUC_{0-\infty}$ (hr*ng/mL)	8	3230 (868)	26.9	2890	2490	4780	3140 (1.28)	24.8
Dn-$AUC_{0-\infty}$ ((hr*ng/mL)/mg)	8	404 (108)	26.8	361	312	597	393 (1.28)	24.7
$t_{1/2}$ (hr)	8	415 (247)	59.6	333	134	838	351 (1.88)	70.0
CL/F (mL/hr)	8	2610 (568)	21.8	2780	1670	3210	2550 (1.28)	24.8
V_z/F (L)	8	1530 (1010)	66.4	1260	620	3490	1290 (1.83)	66.5
**Cohort II: 8 mg Oral 8 to 11 yr (N=9)**								
Cmax (ng/mL)	9	118 (26.5)	22.4	128	70.7	149	115 (1.29)	25.6
Dn-Cmax ((ng/mL)/mg)	9	14.8 (3.31)	22.4	16.0	8.84	18.6	14.4 (1.29)	25.6
t_{max} (hr)	9	-	-	3.95	1.93	4.07	-	-
AUC_{0-24} (hr*ng/mL)	9	1170 (216)	18.5	1200	808	1420	1150 (1.22)	19.8
AUC_{0-48} (hr*ng/mL)	9	1510 (251)	16.6	1530	1040	1820	1490 (1.19)	17.9
AUC_{0-72} (hr*ng/mL)	9	1740 (283)	16.3	1720	1180	2080	1710 (1.19)	17.7
AUC_{0-D7} (hr*ng/mL)	9	2310 (393)	17.0	2410	1530	2720	2280 (1.21)	18.9
AUC_{0-D14} (hr*ng/mL)	9	2860 (533)	18.7	2870	1830	3360	2810 (1.23)	20.9
AUC_{0-D28} (hr*ng/mL)	9	3390 (703)	20.8	3300	2120	4270	3310 (1.25)	23.0
AUC_{0-W12} (hr*ng/mL)	9	4050 (938)	23.1	4170	2490	5560	3950 (1.28)	25.1
$AUC_{0-\infty}$ (hr*ng/mL)	9	3910 (1070)	27.4	3960	2280	5760	3780 (1.33)	29.2
Dn-$AUC_{0-\infty}$ ((hr*ng/mL)/mg)	9	489 (133)	27.3	495	286	720	472 (1.33)	29.0
$t_{1/2}$ (hr)	9	293 (99.2)	33.9	234	177	458	278 (1.40)	34.4
CL/F (mL/hr)	9	2200 (655)	29.8	2020	1390	3500	2120 (1.33)	29.1
V_z/F (L)	9	869 (183)	21.0	911	606	1160	851 (1.25)	22.4
**Cohort II: 6 mg, 8 to 11 yr (N=9)**								
Cmax (ng/mL)	9	89.0 (43.4)	48.8	69.5	59.1	187	82.1 (1.49)	41.2
Dn-Cmax ((ng/mL)/mg)	9	14.8 (7.24)	48.8	11.6	9.85	31.2	13.7 (1.49)	41.2
t_{max} (hr)	9	-	-	3.98	2.00	4.07	-	-
AUC_{0-24} (hr*ng/mL)	9	919 (447)	48.6	722	534	1930	845 (1.51)	43.1
AUC_{0-48} (hr*ng/mL)	9	1180 (593)	50.3	915	665	2530	1080 (1.54)	45.0
AUC_{0-72} (hr*ng/mL)	9	1350 (694)	51.5	1040	760	2940	1230 (1.55)	46.0
AUC_{0-D7} (hr*ng/mL)	9	1750 (945)	53.9	1320	991	3950	1580 (1.57)	47.8
AUC_{0-D14} (hr*ng/mL)	9	2140 (1200)	56.1	1600	1100	4970	1920 (1.60)	49.7
AUC_{0-D28} (hr*ng/mL)	9	2520 (1510)	59.9	1890	1190	6160	2230 (1.63)	52.3
AUC_{0-W12} (hr*ng/mL)	9	3020 (1980)	65.4	2260	1260	7910	2630 (1.69)	56.0
$AUC_{0-\infty}$ (hr*ng/mL)	9	2920 (2120)	72.5	2340	1220	8270	2490 (1.73)	59.2
Dn-$AUC_{0-\infty}$ ((hr*ng/mL)/mg)	9	487 (353)	72.5	391	204	1380	416 (1.73)	59.1
$t_{1/2}$ (hr)	9	280 (134)	47.8	224	181	521	258 (1.50)	41.9
CL/F (mL/hr)	9	2690 (1200)	44.6	2560	726	4900	2410 (1.73)	59.0
V_z/F (L)	9	991 (463)	46.8	1010	496	1870	897 (1.61)	50.6

Supplementary information to:

Opoku et al. Identification of a moxidectin dose for 4-to 11-year-old children to support registration and potential use for onchocerciasis elimination: Results of an open-label pharmacokinetic and safety study in an onchocerciasis endemic area in Ghana

	Statistics							
**Cohort, Dose, Age, N Parameter (unit)**	**n**	**Mean (SD)**	**CV (%)**	**Median**	**Minimum**	**Maximum**	**GM (GSD)**	**GCV (%)**
**Cohort III: 4 mg, 4 to 7 yr (N=9)**								
Cmax (ng/mL)	9	89.4 (24.1)	26.9	89.9	55.9	131	86.4 (1.32)	28.3
Dn-Cmax ((ng/mL)/mg)	9	22.3 (6.01)	26.9	22.5	14.0	32.8	21.6 (1.32)	28.3
t_{max} (hr)	9	-	-	3.95	1.87	4.12	-	-
AUC_{0-24} (hr*ng/mL)	9	863 (179)	20.8	922	645	1110	846 (1.23)	21.3
AUC_{0-48} (hr*ng/mL)	9	1050 (224)	21.3	1070	800	1400	1030 (1.23)	21.2
AUC_{0-72} (hr*ng/mL)	9	1160 (254)	21.8	1160	883	1580	1140 (1.24)	21.5
AUC_{0-D7} (hr*ng/mL)	9	1440 (327)	22.7	1380	1060	1990	1410 (1.25)	22.3
AUC_{0-D14} (hr*ng/mL)	9	1700 (406)	23.9	1690	1210	2380	1660 (1.27)	23.8
AUC_{0-D28} (hr*ng/mL)	9	1940 (504)	26.0	1880	1310	2750	1880 (1.30)	26.5
AUC_{0-W12} (hr*ng/mL)	9	2200 (596)	27.1	2150	1410	3110	2130 (1.32)	28.5
$AUC_{0-\infty}$ (hr*ng/mL)	9	2070 (587)	28.3	1950	1330	2980	2000 (1.33)	29.3
Dn-$AUC_{0-\infty}$ ((hr*ng/mL)/mg)	9	518 (147)	28.3	487	332	745	500 (1.33)	29.3
$t_{1/2}$ (hr)	9	205 (43.3)	21.2	211	137	254	201 (1.25)	22.6
CL/F (mL/hr)	9	2070 (591)	28.5	2050	1340	3010	2000 (1.33)	29.3
V_z/F (L)	9	584 (78.0)	13.4	594	462	724	579 (1.14)	13.5

N: Number of individuals treated, For other abbreviations, see Table S 6
Results for $t_{1/2}$, $AUC_{0-\infty}$, CL and Vz were excluded from the summary statistics if R2adj <0.8
For t_{max} only medians and ranges were displayed

Table S 6: Abbreviations of plasma moxidectin pharmacokinetic parameters

Abbreviation	Description
AUC_{0-24}	Area under the concentration-time curve (AUC) from time zero to 24 h post-dose
AUC_{0-48}	AUC from time zero to 48 h post-dose
AUC_{0-72}	AUC from time zero to 72 h post-dose
AUC_{0-D7}	AUC from time zero to D7 post-dose
AUC_{0-D14}	AUC from time zero to D14 post-dose
AUC_{0-D28}	AUC from time zero to D28 post-dose
AUC_{0-W12}	AUC from time zero to W12 post-dose
$AUC_{0-\infty}$	AUC from time zero to infinity, calculated using the formula AUC_{0-last} + (C_{last} / λz), where Clast is the last quantifiable concentration and λz is the first-order terminal rate constant, calculated by log-linear regression analysis over the terminal log-linear segment of the plasma concentration-time curve. $AUC_{0-\infty}$ is calculated using the linear-up, log down trapezoid rule.
dn-$AUC_{0-\infty}$	Dose-normalized (dn-) $AUC_{0-\infty}$ was calculated as $AUC_{0-\infty}$/dose (mg)
C_{max}	Maximum observed plasma concentration
dn-C_{max}	Dose-normalized (dn-) C_{max} was calculated as C_{max} /dose (mg)
T_{max}	Time of maximum plasma concentration
λz (*)	Apparent terminal elimination rate constant
$t_{½}$	Elimination half-life associated with the terminal slope (λz) of the semi logarithmic drug concentration-time curve, calculated as 0.693/λz
CL/F	Apparent clearance
Vz/F	Apparent volume of distribution
R2adj	R-squared adjusted, Derived to determine $t_{1/2}$
No. of Points	No. points for determination of λz, Derived to determine $t_{1/2}$
$\%AUC_{ext\ (\%)}$	Percentage of the area under the plasma concentration-time curve extrapolated from the time of the last measurable concentration to time infinity as a percentage of AUC_{0-t}, derived to determine $t_{1/2}$

12. Additional information on C_{max} and $AUC_{0-\infty}$ in *O. volvulus* infected adults relative to effect on *O. volvulus* skin microfilariae

In the Phase 2 study, efficacy was assessed by measuring the skin microfilariae density (SmfD) before and 8 days, 1, 2, 3, 6, 12 and 18 months after treatment with a single dose of 2 mg, 4 mg or 8 mg moxidectin or 150 µg/kg ivermectin (7).

The efficacy measures displayed in Fig S 4 for the 74 participants with at least 5 microfilariae/mg skin pre-treatment for whom moxidectin plasma concentrations were obtained were selected based on the following considerations:

1. Day 8 SmfD provides information on the initial SmfD reduction due to the effect of moxidectin on the microfilariae, 21 participants had undetectable SmfD (0 SmfD) at Day 8
2. At Month 1, virtually all skin microfilariae present pre-treatment had been eliminated from the skin: an additional 47 participants had 0 SmfD and SmfD had decreased by between 96% and 99% of pre-treatment SmfD in the remaining 6 participants (who had 0 SmfD at Month 2 or 3).
3. Month 12 is the time at which a next dose would be administered during MDA if MDA with moxidectin follows the most frequent dosing schedule used in Africa for MDA with ivermectin.
4. The change from Month 1 to Month 12 reflects overwhelmingly (see 2. Above) the effect of moxidectin on the reproductive capacity of the macrofilariae.

Supplementary information to:

Opoku et al. Identification of a moxidectin dose for 4-to 11-year-old children to support registration and potential use for onchocerciasis elimination: Results of an open-label pharmacokinetic and safety study in an onchocerciasis endemic area in Ghana

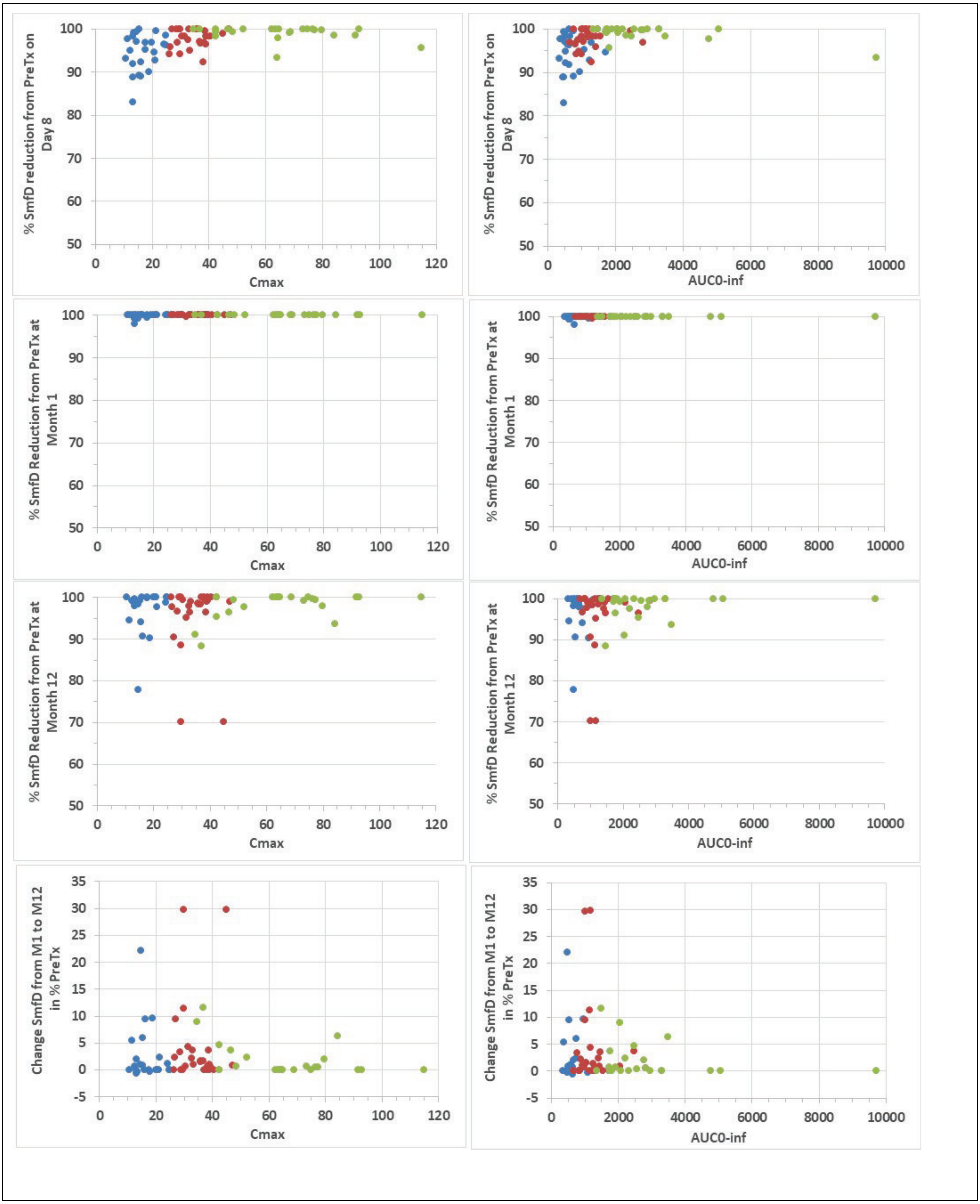

Fig S 4: Measures of moxidectin efficacy vs. C_{max} and $AUC_{0-\infty}$ in *O. volvulus* infected adults with ≥5 mf/mg before administration of 2 mg (blue), 4 mg (red) or 8 mg (green).

13. References

1. Food and Drug Administration of the United States of America Center for Drug Evaluation and Research, Food and Drug Administration of the United States of America Center for Biologics Evaluation and Research,,. Population pharmacokinetics (Series: Guidance for industry). In: United States Department of Health and Human Services FaDAotUSoAUF, Center for Drug Evaluation and Research (U.S.), Center for Biologics Evaluation and Research (U.S.),, editor. Silver Spring, MD: Center for Drug Evaluation and Research,; February 2022.
2. Guideline on reporting the results of population pharmacokinetic analyses, (2007).
3. Korth-Bradley JM, Parks V, Patat A, Matschke K, Mayer P, Fleckenstein L. Relative bioavailability of liquid and tablet formulations of the antiparasitic moxidectin. Clinical Pharmacology in Drug Development. 2012;1:32-7.
4. Korth-Bradley JM, Parks V, Chalon S, Gourley I, Matschke K, cailleux K, et al. The effect of a high-fat breakfast on the pharmacokinetics of moxidectin in healthy male subjects: a randomized phase I trial. Am J Trop Med Hyg. 2012;86(1):122-5.
5. Kinrade SA, Mason JW, Sanabria CR, Rayner CR, Bullock JM, Stanworth SH, et al. Evaluation of the Cardiac Safety of Long-Acting Endectocide Moxidectin in a Randomized Concentration-QT Study. Clin Transl Sci. 2018.
6. Tan B, Opoku N, Attah SK, Awadzi K, Kuesel AC, Lazdins-Helds J, et al. Pharmacokinetics of oral moxidectin in individuals with Onchocerca volvulus infection. PLoS Negl Trop Dis. 2022;16(3):e0010005.
7. Awadzi K, Opoku NO, Attah SK, Lazdins-Helds J, Kuesel AC. A Randomized, Single-Ascending-Dose, Ivermectin-Controlled, Double-Blind Study of Moxidectin in Onchocerca volvulus Infection. PLoS Negl Trop Dis. 2014;8(6):e2953.
